# Predicting microplastic dynamics in coral reefs: presence, distribution, and bioavailability through field data and numerical simulation analysis

**DOI:** 10.1007/s11356-025-36234-5

**Published:** 2025-03-26

**Authors:** Marina F. M. Santana, Hemerson Tonin, George Vamvounis, Lynne van Herwerden, Cherie A. Motti, Frederieke J. Kroon

**Affiliations:** 1 Australian Institute of Marine Science (AIMS), Cape Cleveland Road, Cape Cleveland 4810, Townsville, QLD 4810 Australia; 2 College of Science and Engineering, James Cook University (JCU), Townsville, QLD 4811 Australia; 3 AIMS@JCU, Division of Research and Innovation, James Cook University, Townsville, QLD 4811 Australia

**Keywords:** Marine debris, Abundance, Numerical model, Marine organisms, Consumption

## Abstract

Understanding distribution and bioavailability of microplastics is vital for conducting ecological risk assessments (ERA) and developing mitigation strategies in marine environments. This study couples in situ data from Lizard Island (Great Barrier Reef) and numerical modelling and simulations to determine microplastic abundances in abiotic (water and sediment) and biotic (planktivorous fish, sea squirts, sponges, corals, and sea cucumbers) compartments and predict their trajectories within this ecosystem. Results show microplastics predominantly (75%) originate from beached plastics from nearby islands and coastal areas, dispersing northward without local entrapment and settlement likely occurring on northern beaches (> 50%), including Papua New Guinea. Concentrations increased by three orders of magnitude with depth, with distinct profiles: surface waters contained more fragments and low-density polymers at concentrations of < 1 microplastics m^−3^, and deeper layers more fibres and high-density polymers, with concentrations peaking at the seafloor at > 100 microplastics m^−3^. Reflecting ecological and physiological traits of each taxon, fish exhibited microplastic contamination levels nearly twice that observed in invertebrates, and while polymers and colours had no stronger evidences on influencing bioavailability, shape and size did, with fish more susceptible to contamination by microplastic fibres and all taxa to smaller-sized microplastic particles.

**Supplementary Information:**

The online version contains supplementary material available at 10.1007/s11356-025-36234-5.

## Introduction

Contamination of marine ecosystems by plastics and microplastics (1 µm to 5 mm in size) is considered an environmental priority of global importance (United Nations Environment Programme 2021). While there is a plethora of knowledge regarding larger plastics (United Nations Environment Programme 2021), there is less known about microplastics and empirical data is urgently needed to establish effective microplastic-specific ecological risk assessments (ERA) (Environmental Protection Agency 1992) to support environmental management and mitigation strategies. ERAs are a vital tool to evaluate the likelihood and consequences of exposure to a stressor, and requires the systematic examination of the scientific literature to establish (1) which risk factors are important and (2) the relationship(s) between stressor exposure and observed (i.e. measured) ecological impacts (Environmental Protection Agency 1992, GESAMP 2016). In essence, ERAs are used to describe how, when, and where exposure occurs and at what point the risk is realised. As such, the framework for an ERA includes two key phases: detailed characterisation and profiling of the exposure and evaluation of the ecological effects resulting from said exposure.

For microplastics, comprehensive and robust exposure characterisation data that links stressor exposure to ecological impacts is challenging on a global scale given that, locally, microplastic contamination across abiotic matrices (i.e. surface waters, mid-water columns, and seafloor sediments) can exhibit considerable variation in concentration and characteristics due to the heterogeneity and diversity of polymers (and their additives) that enter the marine environment (Rochman et al. 2019) as well as the environmental factors (Amenabar et al. 2024; Gunaalan et al. 2024; Manullang et al. 2024) and biological interactions (Kvale et al. 2020; Miller et al. 2022a; Sacco et al. 2024). Thus, to define the global threat, it is first necessary to conduct comprehensive local-scale assessments to determine presence and distribution of microplastics and reveal microplastic transport pathways and environmental fates. Combining local field data with hydrodynamic numerical simulations thereafter enhances the sensitivity and accuracy of these assessments, providing critical insights into microplastic spatial and temporal dynamics at both regional (Jensen et al. 2019; Vega-Moreno et al. 2024) and global (Everaert et al. 2020; Ourmieres et al. 2023; van Sebille et al. 2015) scales.

Establishing the link between exposure and ecological impacts (Environmental Protection Agency 1992) requires assessment of microplastic contamination in biotic matrices to understand the extent and pathways of contact and confirm field bioavailability. Comparative analyses between abiotic and biotic contamination locally can refine our understanding of bioavailability, classify microplastic characteristics according to the specific risks they pose to organisms, and help identify effective bioindicator species (Pastorino and Barceló 2024). Differences among biotic matrices can also reveal species-specific contamination patterns related to an organism’s ecological traits and physiology, with many studies having already highlighted the complexity of microplastic contamination in marine organisms (Sacco et al. 2024). Yet, despite global interest, the full extent and level of the ecological risk remain uncertain, mostly due to inconsistent and incomplete data at local scales (Jung et al. 2021; Wootton et al. 2024).

Due to the deficit of microplastics data, current ERAs rely on studies that are focused on isolated compartments (e.g. abiotic or biotic compartments) (Everaert et al. 2020; Fraissinet et al. 2024; Gao et al. 2024; Lefebvre et al. 2024) and which lack comprehensive coverage across different compartments and organisms. Furthermore, works integrating field-derived microplastics data with numerical simulations are limited, even though this information is crucial to validate and refine modelling exercises (Moodley et al. 2024). Similarly scarce, although now a point of focus for the microplastics field, is the application of standardised methods for sample collection, processing, and data reporting (Hartmann et al. 2019; Lusher et al. 2020; Prata et al. 2020; Wootton et al. 2024). Thus, intense research effort is needed to address these deficiencies, with holistic localised studies key to understanding and predicting what is occurring at the regional and global scales.

Given their extraordinary diversity, ecological importance, and high sensitivity to anthropogenic stressors (Sobha et al. 2023), tropical coral reefs are an exemplar ecosystem for understanding the status of ocean and planet health. In this context, and the fact that microplastics have been identified globally as a contaminant of concern, understanding their impacts on coral reefs is considered of critical importance (Biswas et al. 2024; Huang et al. 2021; Rahman et al. 2023). Existing studies have documented the presence of microplastics in coral reef waters (Nie et al. 2019; Tan et al. 2020), sediments (Lin et al. 2024; Patti et al. 2020; Portz et al. 2020), and biota (La Beur et al. 2019; Rotjan et al. 2019), yet these are not representative of the global spatial coverage of this ecosystem type (approximate 284,300 km^2^ across more than 20 countries, Global Coral Reef Monitoring Network, GCRMN), nor of its entire biodiversity (being the most biodiverse ecosystem in the world). Even so, coral reef ecosystems are now considered microplastic sinks (Martin et al. 2019; Reichert et al. 2022; Soares et al. 2023) and therefore at risk. In recent critical literature reviews (Huang et al. 2021; Rahman et al. 2023), less than 30 field studies on microplastics in coral reef ecosystems were considered for analysis, with more recent reviews identifying similar limitations (Lin et al. 2024; Shaw et al. 2024). The scant knowledge of microplastic trajectories in coral reef systems (Critchell et al. 2015; Jensen et al. 2019) similarly reflects the paucity of hydrodynamical modelling studies. This scarcity of literature regarding microplastic abundance and distribution in coral reefs (Huang et al. 2021; Rahman et al. 2023), and the knowledge that microplastics distribution can be highly variable in coral reefs systems due to local reef dynamics (Rahman et al. 2023), continues to hamper the ecological analyses required to establish microplastic distribution patterns and exposure thresholds above which adverse effects intensify (Environmental Protection Agency 1992).

This study addresses fundamental questions related to the ecological risks that microplastics pose to coral reef ecosystems by testing the hypothesis that microplastics accumulate within coral reef ecosystems, increasing ecological risks through ingestion by biota. To investigate this, a comprehensive field-based dataset was generated, and used to develop numerical models to assess microplastics presence, distribution and (dis-)similarities among abiotic and biotic compartments of a tropical coral reef. Abiotic (surface and mid-column waters and seafloor sediment) and biotic (planktivorous fish, sea squirt, sponge, hard coral, and sea cucumber) matrices were sampled from two unique coral reefs at Lizard Island, within the Great Barrier Reef World Heritage Area (GBRWHA), Australia, to establish a baseline of microplastic contamination at Lizard Island. Thereafter, data was used in numerical hindcast and forecast simulations to predict the trajectories of microplastics within a 2-week period of the sampling time. Bayesian regression modeling was used to analyse the relationship between microplastic characteristics and levels of contamination across taxa and their surrounding abiotic compartment, and determine the extent of exposure to and bioavailability of microplastics. Notably, this is the first study to investigate the full vertical profile of microplastic contamination, from the seasurface to the mid-column and seafloor sediment, within a coral reef ecosystem (Huang et al. 2021; Rahman et al. 2023) while also contextualising the risk for inhabiting species.

## Methods

### Lizard Island group

**Fig. 1:**
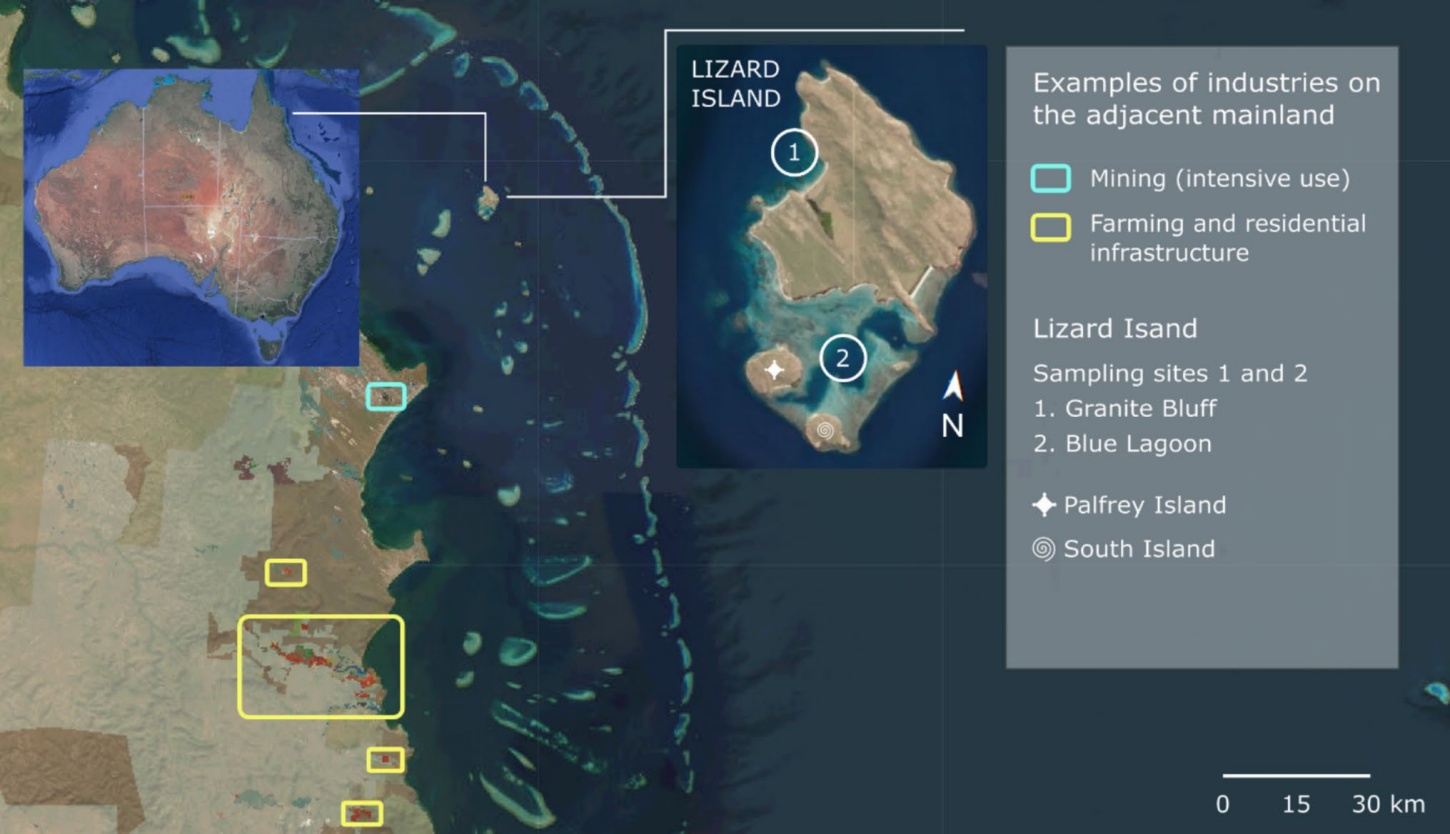
Location of the Lizard Island group, and sampling sites Granite Bluff (1) and Blue Lagoon (2), in the northern Great Barrier Reef World Heritage Area, Australia with areas of mining industries (blue box) and farming and residential infrastructure (yellow boxes) shown on the adjacent mainland. This figure was adapted from a map extracted from the Queensland State-Wide Land Use Map (www.qld.gov.au) on November 30, 2022

The Lizard Island group (14°40′08″S 145°27′34″E) consists of three islands: Lizard, Palfrey, and South Islands, which are located in the northern GBRWHA, approximately 30 km northeast of the Australian continent (Fig. 1). Despite its relatively remote location, previous studies have reported microplastic contamination in resident fish (Santana et al. 2021) and regionally (Kroon et al. 2018b). Yet there exists no direct evidence linking the surrounding anthropogenic activities, whether from the islands or the mainland, to microplastic pollution levels in the abiotic and biotic compartments of the Lizard Island group. The Lizard Island group is 250 km north of the largest city in the region (Cairns; population ∼167,000) and located within a designated shipping area within the GBR Marine Park (amsa.gov.au). Industries on the adjacent mainland are classified as “residential and farm infrastructure” and “intensive uses”, which includes mining (qld.gov.au). Most of the surrounding marine environment of Lizard Island is zoned as Marine National Park restricting extractive and recreational uses, with Lizard Island itself hosting a tourist resort, a research station, and a camp site. Beach clean-up surveys have identified substantial marine debris contaminating the Lizard Island group coastlines (tangaroablue.org), with plastic remnants representing more than 70% of the debris collected on the beaches.

The coral reef ecosystem of the Lizard Island group comprises narrow fringing reefs surrounding most of the main island. Samples were obtained from two distinct locations: the more exposed Blue Lagoon and the sheltered Granite Bluff. This sampling approach captures coral reef systems under contrasting hydrodynamic conditions, providing a more comprehensive understanding of microplastics dynamics. The Blue Lagoon reef (~ 2 km × 2 km × 10 m deep) is positioned between Palfrey and South Islands, with a 3-m tidal range and fast-flowing currents (Hamylton et al. 2014). Granite Bluff (~ 1 km × 1 km × 10 m deep) is a sheltered northwest facing reef adjacent to Watson’s Bay (Fig. 1). The hydrodynamics of the Lizard Island group are strongly influenced by winds (Frith et al. 1986) predominantly emanating from the southeast (referred to as the Southeast Trade wind) between March and September. These trade winds reach speeds of up to 30 m s^−1^ (Hamylton et al. 2014) and circulate to the northwest (Frith et al. 1986). From October to February, the wind direction is unpredictable, as are circulation patterns which present more frequent current reversals and cross-shelf motion (Frith et al. 1986; Philipps and Bellwood 2024). Northwest winds are the second most common Lizard Island wind feature, with maximum speeds of 20 m s^−1^ (Hamylton et al. 2014). The Lizard Island group is also susceptible to tropical cyclones during the summer months, and can experience increased wind speeds and disturbance of shallow coral reefs (Lassig 1983; Madin et al. 2018).

### Sample collection

Samples were collected from the Lizard Island group from 1 to 12 October 2018, a period that coincides with the onset of the monsoon winds. Sampling of biota was in accordance with animal ethics (James Cook University permit A2506) and collection regulations (Great Barrier Reef Marine Park Authority permit G12/35236.1). For each abiotic sample (surface and mid-column waters, and seafloor sediment), five replicates were collected per site (*N* = 10 samples per abiotic sample). For each biotic taxon (planktivorous fish, hard coral and sea cucumber, and, for the first-time, sea squirt and sponge, ten individuals were collected per site (*N* = 20 per taxon). Collections were conducted by neuston tows (surface water), a submersed pump sampler (mid-column water), and SCUBA (seafloor sediment and organisms); specific collection details are described in the Supplementary information. Wherever possible, sampling locations were evenly distributed across each site, with the caveat that organismal sampling was also guided by the availability of target species. Species sampled were *Pomacentrus amboinensis* (fish — pelagic planktivore), *Polycarpa pigmentata* (sedentary benthic sea squirt — filter feeder), *Neopetrosia chaliniformis* (sedentary benthic sponge — filter feeder), *Dipsastraea lizardensis* (sedentary benthic hard coral — suspension feeder), and *Holothuria edulis* (sea cucumber — mobile benthic deposit feeder). Relevant weather conditions at the time of field sampling were obtained from the Australian Bureau of Meteorology (BOM; http://www.bom.gov.au/, Cape Flattery weather station). Information on wind (i.e. speed and direction) was sourced from the Lizard Island weather station, which forms part of Australia’s Integrated Marine Observing System (IMOS, www.imos.org.au). Environmental conditions were used to contextualise the sampling period, with wind incorporated into numerical simulations to model microplastic trajectories.

### Sample processing

At LIRS, surface tow samples were filtered into stacked 263- and 37-µm mesh sieves. The retained items on each sieve were transferred to 50-mL jars (polypropylene (PP) container, high-density polyethylene (HDPE) screw cap; Sarstedt) and preserved in 70% EtOH (to a final volume of 50 mL) for processing and analysis at the Australian Institute of Marine Science (AIMS) in Townsville. Similarly, contents of mid-column samples retained on stacked 263- and 37-µm mesh sieves (i.e. already concentrated during field collection) were also transferred into 50-mL jars as per above. Sediment samples were frozen at − 20 °C for transportation and processing at AIMS. All individual organisms were measured (0.1 cm, Kincrome calliper, 1/1000 in) and weighed (0.01 g, AND EK Balance—410i), with dry weight of organisms used for estimations of microplastic concentration and data analysis (Table S2). Individual coral and sponge samples were transferred into 600-mL PP jars. The gastrointestinal tracts (GIT) of fish and sea cucumbers and the tunic and pharynx of sea squirts were removed by dissection, and individual GIT contents and sea squirt innards were transferred to 50-mL PP jars and sealed with HDPE lids. All biological material was preserved in 70% EtOH for transport and storage prior to processing and analysis at AIMS laboratories.

#### Water samples

Surface water samples were processed using a density separation method adapted from Kroon et al. (2018a). Briefly, samples were transferred to 400-mL glass beakers and 300 mL of 1.2 g cm^−3^ sodium chloride (NaCl; AR, Fisher Chemical, CAS No. 7647–14-5) solution was added. Using a handheld glass stirring rod, the solution was gently stirred for 3 min to achieve uniform contact of particulates with the brine solution, thereby enhancing microplastic recovery while also minimising the potential for further fragmentation especially of weathered plastics (Pramanik et al. 2021). The sample was then left overnight (~ 18 h) to settle. Approximately 100 mL of the surface supernatant was then decanted into a second glass beaker and an additional 100 mL NaCl solution added to the remaining 200 mL of sample. The sample was again stirred for 3 min, left to settle for 1 h, and a second 100 mL of surface supernatant syphoned into the aforementioned 2nd glass beaker. This process was repeated once more. The combined decanted supernatant (total ~ 3 × 100 mL) was filtered through a customised filtration apparatus consisting of tiered 263- and 26-µm stainless steel filters (Schlawinsky et al. 2022).

To process mid-column water samples, a similar protocol was used as described above, replacing NaCl with 1.7 g cm^−3^ potassium iodide (KI; AR, Univar, CAS No. 7681–11-0) (Santana et al. 2022b) based on the assumption that denser microplastics would likely be present in this abiotic matrix (Uddin et al. 2021; Woodall et al. 2014). In addition, to avoid disturbing settled material, the top 100 mL of supernatant was syphoned rather than decanted into a second beaker using a silicone tube.

#### Seafloor sediment samples

Frozen sediment samples were lyophilised (~ 2 days; Dynavac Freeze-drier) and processed using 1.7 g cm^−3^ KI density separation as per mid-column water samples. An additional 547-µm stainless-steel filter was added to the top of the z-stacked filtration apparatus to capture larger items, e.g. shell fragments, fine sand particles, and organic matter.

#### Organisms

A total of ten organisms per species were used for analysis. Each fish GIT was cut open using dissecting scissors and contents scraped into a Bogorov counting chamber for exhaustive visual inspection under stereomicroscope (Leica M165C, × 0.73– × 12.0 magnification) (Jensen et al. 2019; Kroon et al. 2018b). Putative microplastics identified in each fish GIT were picked from the chamber using a glass pipette and transferred to 26-µm stainless steel filters for further physical and chemical characterisation. Each fish GIT wall was inspected in a petri dish containing Milli-Q water. All invertebrates were processed following Santana et al. (2022b)) with some slight modification, noting that the processing methods had only minimal impact of the gross characteristics of spiked microplastics (Santana et al. (2022b)— also see “Recovery rates”). Briefly, corals were digested in 70% nitric acid (HNO_3_; AR, Univar, CAS No. 7697–37-2) for 6 h. Sponges were digested following a stepwise protocol, in which samples were first digested in 70% HNO_3_ for 6 h and residual material density separated using an overnight 1.7 g cm^−3^ KI procedure. Sea squirt innards were digested in 70% HNO_3_ for 24 h. Lastly, each dissected sea cucumber GIT was excised, and contents removed and processed using 1.7 g cm^−3^ KI. All clarified invertebrate samples were subsequently filtered through the stainless steel filtration apparatus onto stainless steel filters, as described above (Schlawinsky et al. 2022).

### Microplastic identification and characterisation

Microplastic physical and chemical characterisation was done following (Kroon et al. 2018a) with some modifications. First, all putative microplastics were visually identified using stereomicroscopy (Leica MZ16A) and microphotographed (Leica DFC 500, Leica Application Suite LAS 4.4.0). All putative microplastics were then characterised based on size, shape, and colour (Hidalgo-Ruz et al. 2012, Santana et al. 2022a). Size (maximum length) was measured using Fiji (Image J software), and items were grouped into one of five size classes (class 1, > 5 mm; class 2, < 5 mm and>2.5 mm; class 3, < 2.5 mm and > 1 mm; class 4, < 1 mm and > 500 µm; and class 5, < 500 µm). Class 1 was included to capture any plastics larger than the micro scale. Shape (irregular fragment, or fragment, and fibre) and colour (black, blue, brown, green, grey, orange, pink, purple, red, transparent, white, yellow, and mix (i.e. mixture of more than one colour) of all examined items were adapted from Santana et al. (2022a). Polymer composition of every putative microplastic was chemically confirmed by Fourier-transform infrared spectroscopy (FTIR), with spectral quality confirmed using the PerkinElmer polystyrene (PS) reference sample. Spectrum collection and treatment are described in Supplementary information. Collected spectra were searched against NICDOCOM IR spectral libraries (Polymers and Additives, Coatings, Fibers, Dyes and Pigments, Petrochemicals; NICODOM Ltd., Czechia, excluding CO_2_ and H_2_O ranges) for final chemical assignment. A similarity threshold of 70% to a reference spectrum was required to establish the plastic nature of an item (Kroon et al. 2018a). Items with similarity rates between 60 and 70% were further assessed and if spectral quality was deemed good, these items were retained in the dataset (Kroon et al. 2018a).

All items were subsequently categorised based on Kroon et al. (2018b) (Table S3) with some modifications as either (i) synthetic (i.e. manufactured by chemical synthesis) or semi-synthetic items (i.e. composite synthetic-natural items, or synthetically modified natural materials, e.g. rayon, cellophane), (ii) naturally derived (i.e. items manufactured from natural materials, e.g. cotton), and (iii) natural (i.e. not manufactured). Items categorised as semi-synthetic were assigned based on their synthetic component. Synthetic items comprising more than one synthetic polymer were grouped based on their primary synthetic polymer type.

### Quality assurance and quality control

#### Recovery rates

All separation methods applied were validated using spike-recovery tests prior to use. Spike-recovery tests consisted of three replicates each of surface and mid-column water, sediment (~ 3 g, d.w.) and organism (~ 3 g of tissue, d.w.). All samples were spiked with 15 environmentally and ecologically relevant microplastic particles globally and regionally (Jensen et al. 2019; Kuhn et al. 2018; Miller et al. 2022a, 2022b, Santana et al. 2022b), including irregular shaped particles (< 1.0 mm) of yellow PE (*N* = 5); transparent polystyrene (PS, *N* = 5), and monofilament fibres (approx. 2 mm) of black rayon (*N* = 5). Spiked samples were then processed as described above for field samples and recovered microplastics were visually identified and counted using a stereomicroscope (Leica MZ16A).

#### Microplastic contamination control

Prevention and monitoring of extraneous microplastic contamination followed protocols described in Kroon et al. (2018a) and Santana et al. (2021). Plastic-based field and laboratory gears were avoided where possible and cleaning procedures and airborne blanks were applied to record extraneous contamination (details provided in Supplementary information).

The unavoidable use of plastic-based utensils during sample collection and processing was acknowledged, and a representative sample of each utensil was collected and added into a project-specific plastic contaminant library (Table S4) (Kroon et al. 2018a). In addition, any microplastics captured during monitoring for airborne contamination were added to the customised contaminant library. To establish and correct for unintentional contamination, all microplastics found in samples were compared against those in the contaminant library (Kroon et al. 2018a). Specifically, if an item returned a ≥ 90% spectral match to the contaminant library, and also matched in shape and colour, it was considered to be extraneous contamination and excluded from further analysis (Dawson et al. 2023; Kroon et al. 2018a).

### Microplastic tracking through numerical modelling

Predicting the pathways of plastic debris in general and microplastic specifically can be challenging (Griffin et al. 2016, Potemra 2012). Here, numerical simulations were used to hindcast and forecast the distribution of the microplastics collected around the Lizard Island group. Two-dimensional high-resolution hydrodynamic numerical simulations were conducted using the Delft3D integrated modelling suite (Deltares 2016) (with 500 × 500 m resolution) and the General NOAA Operational Modelling Environment (GNOME; with 4 × 4 km resolution) (Zelenke et al. 2012). Delft3D was centred at the Lizard Island group, covering a spatial domain approximately 600 km along the coast and 100 km cross-shore (Figure S1), and forced on its open boundaries by the eReefs output (Herzfeld et al. 2016; Schiller et al. 2014) to improve model stability. The model was then simulated for the period from September 2018 to October 2018. Trajectories of virtual particles, as proxies for floating microplastics, were computed by GNOME. Those trajectories extending beyond the pre-established 500 × 500 m Delft3D domain were modelled based on global currents (with 4 × 4 km resolution), applying the coarser spatial and temporal resolution used by GNOME. Wind input data in the models (sourced from the Lizard Island wind station, with IMOS data reduced to 10 m height) was collated hourly by calculating a moving average of 6 × 10 min measurements and found to have a 10% directional uncertainty (assigned in both Cartesian axes, *x* and *y*). In our modelling exercise, the influence of wind on the deflection (windage) of the virtual particles was modelled within a windage range from 0 to 4% (calculated in 1% increments), covering the windage range usually used in the literature (van Utenhove 2019, Zelenke et al. 2012) (Table S5) for non-extreme weather events. Using the numerical model, hydrodynamic currents were calculated at 1-h intervals and, as for wind, a 10% uncertainty for both Cartesian *x* and *y* components was considered in the particle tracking exercise.

The model parameters included sampling site (Granite Bluff and Blue Lagoon), event date (4–7 October 2018) and time (0900 h–1200 h) as the reference (*t*_0_). The relative abundances of microplastics found in surface and mid-column water samples per sampling site were combined, and the ratio of total microplastics per sampling site (63% for Granite Bluff and 37% for Blue Lagoon) was used to determine the number of virtual particles seeded for each simulation. To ensure robust outputs, for every 1 microplastic found, 10 virtual particles were seeded (Table S5), and uncertainties of 10% assumed, as described above. Numerical simulations were conducted over one lunar month, centred on the sampling event date, to hindcast (reverse tracking) and forecast (forward tracking) microplastic trajectories at both sampling sites. The predicted locations of microplastics 14 days prior to (i.e. hindcasting) and after (i.e. forecasting) collection were recorded, and categorised as follows: (1) beached – best guess, (2) beached – uncertain, (3) floating – best guess, and (4) floating – uncertain. “Best guess” categories are based on hydrodynamic and wind fields but do not consider the uncertainty associated with their vectorial fields (i.e. numerical simulation used current and wind fields as they were entered into the model). “Uncertain” categories considered the 10% of uncertainty associated with such fields (independently or simultaneously) (Williams and Esteves 2017).

### Statistical analyses

All statistical data analyses were conducted using RStudio version 2023.12.0. Differences in microplastic concentration within abiotic (microplastics m^−3^) (Eq. 1) or biotic (microplastics g^−1^ of tissue processed) (Eq. 2) matrices were investigated by linear models (LM, *p* < 0.05).

1 $$\text{concentration of microplastics}=\text{matrix}+\text{sampling site}+\text{constant}$$

2 $$\text{concentration of microplastics}=\text{matrix}+\text{constant}$$

where, matrix = surface water, mid-column water, or sediment (i.e. abiotic matrix, Eq. 1); or fish, sea squirt, sponge, hard coral, or sea cucumber (i.e. biotic matrix, Eq. 2), sampling site = Granite Bluff or Blue Lagoon (Eq. 1).

Best-fit models were chosen using an Akaike Information Criterion (AIC) test and validated using residual diagnostics (Figures S2 and S3). Based on that, the influence of sampling site was included when assessing differences in microplastic concentration within abiotic (Eq. 1) but not biotic matrices (Eq. 2). To account for skew and overdispersion of data, models were fitted using Poisson distribution for the logarithmic transformation of the abiotic matrices, while a square root transformation was used for the biotic matrices.

To assess (dis-)similarities in profile of microplastics (shape, size class, colour, and polymer type) contaminating the abiotic and biotic compartments, Bayesian regression analyses were conducted with categorial distribution and logit link (Eq. 3).

3 $$\text{microplastic characteristic }\sim 0+\text{Intercept}+\text{ matrix}$$

where, microplastic characteristic = shape, size class, colour, or polymer type, and matrix = mid-column water and fish, sea squirts, sponges, or seafloor sediment and sea cucumber. Microplastic characteristics from the surface waters were not included in this analysis as none of the organisms collected inhabits this abiotic compartment. Bayesian numerical simulations were conducted using four chains, one core, and 2000 iterations. Model validation and analyses were conducted using the brms package (version 2.16.3). Influence of priors in the posterior distribution, as well as chain convergence and residuals, was assessed for each model to validate whether these were an adequate fit to the data (data not shown). Posterior predictive checks were used to investigate how well the models replicate the overall distribution of the data (Figures S4–S7). Differences between abiotic and biotic matrixes were assessed using the R package “emmeans” (version 1.7.2) and posterior distribution of differences expressed as difference between abiotic and biotic samples. Thus, differences between both treatments were expected to be centred around 0, representing no difference between control and sample treatments. Yet, for this to be strong evidence, the 95% credible intervals were expected to overlap 0; otherwise, the difference observed was considered unreliable. Susceptibility of taxa to risks associated with environmental microplastics was inferred based on the (dis-)similarities of microplastics. For example, similar microplastic profiles in both biotic and abiotic compartments indicate high susceptibility, although other environmental factors can influence the presence of the microplastics in the environment in the first place. Conversely, differences in microplastic profiles suggest that selectivity or physiological processes play a more significant role for susceptibility than the presence of the microplastic in the environment.

## Results

### Environmental conditions

From 1 to 12 October 2018, the maximum air temperature at the Lizard Island group ranged from 29.2 to 31.1 °C (Table S6). No rain was recorded. As expected for this time of year (Frith et al. 1986), 81% of the winds were from the east-southeast- southeast (Figure S8, Table S5). Overall, wind intensity varied between 4 and 10 m s^−1^ for 65% of the time and was < 4 m s^−1^ for the remaining time; Granite Bluff and Blue Lagoon wind speeds ranged from 0 to 5 knots and 0 to 20 knots, respectively. Both wind direction and speed influenced microplastic distributions at each site (refer below). Swell at both sites varied little, from 0 to 0.5 m.

### Quality assurance and quality control (QA/QC)

Overall, microplastic recovery rates of > 70% were achieved from spiked abiotic and biotic matrices treated with the relevant separation method, i.e. NaCl and KI density separation and 70% HNO_3_ digestion. Rayon monofilament fibres were the exception, having generally lower recovery rates, especially from sponge tissue (66. 7 ± 9.4%) (mean ± standard deviation (SD)) (Table S7). For this reason and given there remains no consensus as to whether rayon and other modified natural fibres should be classified as microplastics (Hartmann et al. 2019; Rathinamoorthy and Balasaraswathi 2024; Stark 2019), all natural-based/derived anthropogenic items (e.g. textile cotton, including rayon, or wool) were excluded from the final dataset. Blended items having natural-based polymers as a co-component with a synthetic plastic polymer (e.g. fibre blend of cotton with nylon) were identified based on the synthetic polymer (e.g. nylon) (Table S3). Recovery rates for the yellow irregular particles of PE were 100% from all matrices, except sponges (93.3 ± 9.4%). Recovery rates of transparent PS fragments ranged from 80.0 ± 16.3% (sea cucumber) to 93.3 ± 9.4% (for surface and mid-column waters and sponge samples).

In total, 2137 putative microplastics were isolated across all abiotic and biotic samples (Table S8). Of these, 324 were excluded because the chemical composition could not be confirmed (i.e. poor quality of acquired FTIR spectra, *N* = 103 or 4.8% of the total number of items analysed), or because an item matched physically and chemically with an item in the contaminant library (*N* = 221 or 10.4%). Another 1277 items were further excluded based on being assigned as either natural debris or an anthropogenic item derived from a natural polymer (e.g. cellulose and wool), including rayon. The remaining 536 (25.1%) putative microplastics were confirmed by FTIR to be synthetic (*N* = 481, 90%) and semi-synthetic (*N* = 55, 10%) polymers (Table S8).

### Microplastic presence and abundance in abiotic and biotic matrices

**Fig. 2:**
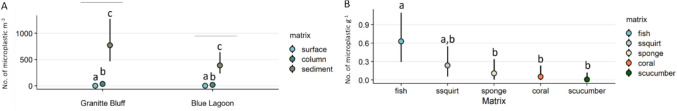
Microplastic counts m^−3^ (**A**) in surface and mid-column waters, and seafloor sediment at the two sampling sites (Granite Bluff and Blue Lagoon, displayed separately); and total microplastic counts g^−1^ (**B**) in fish, sea squirt, sponge, coral, and sea cucumber (two sampling sites combined). Colour key insets for (A) and (B) represent individual matrices. Circles and vertical lines represent estimated marginal means and 95% confidence intervals, respectively. Lowercase letters (a, b, c) represent significant differences (*p* < 0.05) among the (A) three abiotic and (B) five biotic matrices, respectively. Horizontal bars represent differences (*p* < 0.05) in microplastic contamination between the two sampling sites for the abiotic compartment specifically

Microplastic contamination was prevalent at the two Lizard Island sampling locations, with all abiotic (i.e. surface and mid-column waters and sediment) and biotic (i.e. fish, sea squirt, coral, sponge, and sea cucumber) matrices containing microplastics. At each sampling site, mean concentrations (±SD) in abiotic compartments increased by three orders of magnitude with depth, i.e. surface < mid-column < seafloor sediment (all *p* < 0.05, Fig. 2A, Tables S9, S10a). Furthermore, the abiotic compartments of Granite Bluff were significantly more contaminated with microplastics compared to Blue Lagoon (*p* < 0.05). For the biotic matrices, the model exploratory analysis (AIC) indicated best fit if sampling site was not considered a factor in the analysis; therefore, differences between sites were not investigated here. Microplastics were detected in 70 to 80% of all taxa, except for sea squirts, in which 50% of individuals showed contamination. Overall, contamination levels in fish were 1.6 to 2 times higher than sponges, corals, and sea cucumbers (*p* < 0.05, Fig. 2B, Tables S9, S10b), but were not significantly higher than those in sea squirt. Levels of contamination amongst different invertebrate taxa were not statistically different.

### Modelled microplastic trajectories based on the hydrodynamics of Lizard Island

**Fig. 3:**
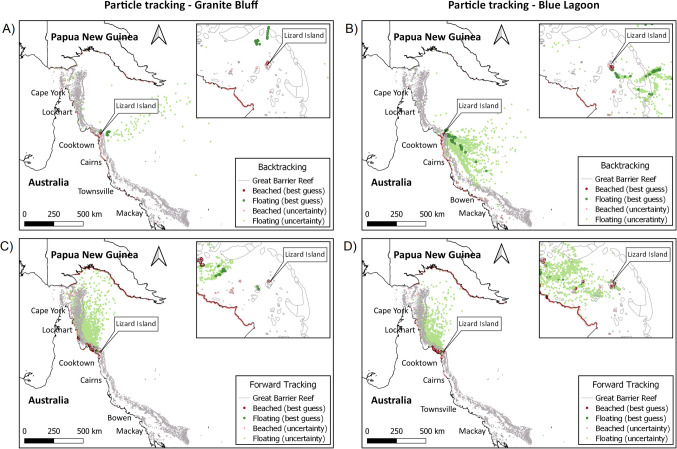
Results of the numerical simulation for the virtual particles seeded at Granite Bluff (A, C) and Blue Lagoon (B, D). (**A** and **B**) Hindcasting trajectories 14 days prior to collection. (**C** and **D**) Forecasting trajectories 14 days after collection. Panel insets show the respective result centred on Lizard Island. Shades of red represent beached particles and shades of green floating particles. The “best guess” results are represented by the more intense tones of red and green, the “uncertain” results being pale in tone

The outputs from the numerical hindcasting simulations (reverse tracking) show that at least 75% of microplastics found in surface and mid-column waters were likely to have originated from nearby (or local) beaches. Under the best-guess scenario, 39% of microplastics collected in the waters of Granite Bluff were deemed to have come from the coastal embayment (Fig. 3A, Table 1), while up to 34% of those at the Blue Lagoon had trajectories identifying the south coast of Lizard Island and nearby southern islets as sources (Fig. 3B, Table 1). When considering only the uncertainties in the numerical simulations, > 30% of microplastics found at both sampling sites could also have a continental source. The model predicted these microplastics originated from the coastal region of northeast Australia, spanning approximately 1000 km of coastline, mostly between Mackay and Bowen (south) and from between Cape Melville and Cooktown (north). Regardless of the scenario modelled, the trajectories of the virtual particles indicated sampled microplastics were unlikely to have been at sea 14 days prior to collection with only 22% and 35% floating in waters adjacent to Granite Bluff and Blue Lagoon, respectively. For Granite Bluff, these floating virtual particles are predicted to originate from the eastern regions of Lizard Island. Conversely, for Blue Lagoon, they are predicted to originate from the region southeast of Lizard Island, at the edge of the Coral Sea.

**Table 1:** Percentage of virtual particles beached and floating at the end of the 28-day numerical simulation, including hindcast (*T*_0_ minus 14 days) and forecast (*T*_0_ plus 14 days) modelling, for Granite Bluff and Blue Lagoon sites

	Granite Bluff		Blue Lagoon	
	Hindcast	Forecast	Hindcast	Forecast
% Beached (best guess)	39	34	34	40
% Beached (uncertainty)	39	25	31	29
Total % beached	78	59	65	69
% Floating (best guess)	11	16	16	10
% Floating (uncertainty)	11	25	19	21
Total % floating	22	41	35	31

The outputs for the numerical forecasting simulations (forward tracking) indicated a general west-northwest directional pattern for the movement of microplastics collected at Lizard Island, aligning with the prevailing winds during this period (Fig. 3C and D, Table 1). In the best guess scenario, a portion of microplastics (34% and 40% from Granite Bluff and Blue Lagoon, respectively) dispersed from Lizard Island and beached on islands and coastal areas of the Australian mainland, such as Cape Melville National Park and Lockhart. Although less likely, the beached uncertainty simulations predicted microplastics could also travel as far as Cape York and eventually Papua New Guinea (PNG). When considering both the best guesses and the uncertainties, the likelihood of microplastics ending up on shore, including as far as PNG, are 59% and 69% for Granite Bluff and Blue Lagoon, respectively.

### Profile of microplastic contamination in abiotic matrices

**Fig. 4:**
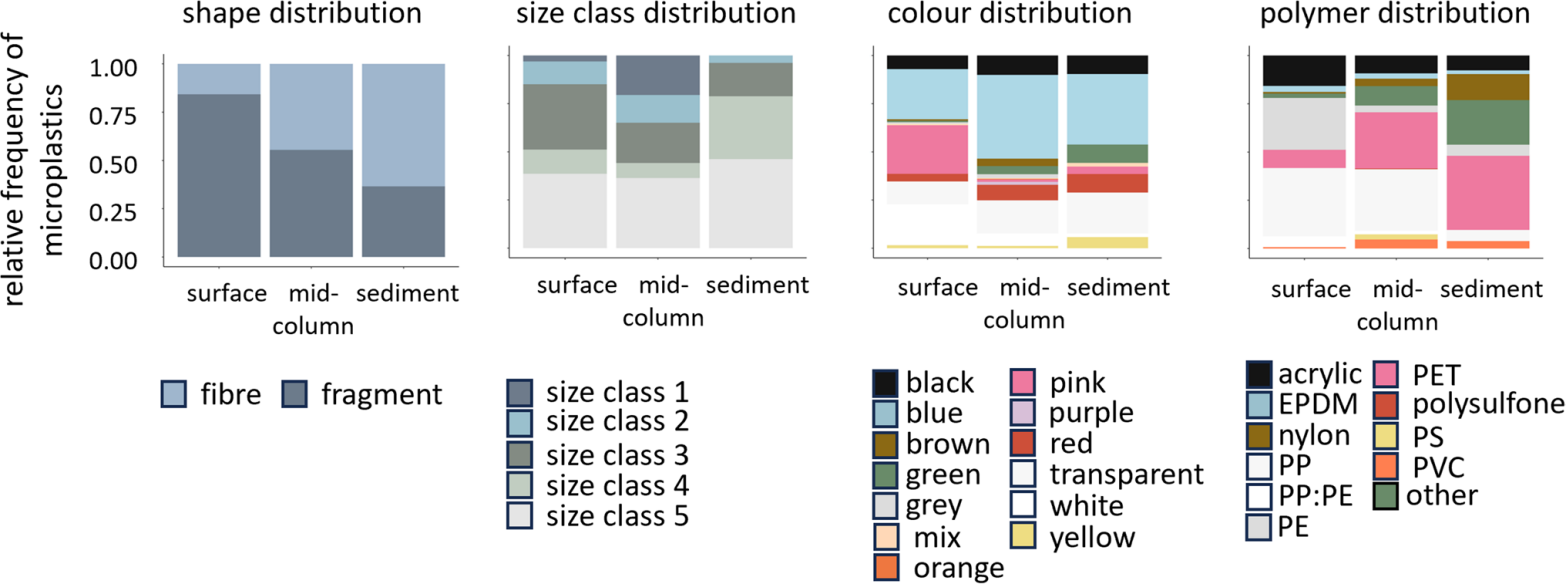
Relative number of microplastics found in the abiotic sea surface and mid-column waters and seafloor sediment compartments at Lizard Island, in relation to shape, size class, colour, and polymer distribution

Within the abiotic matrices, the proportion of microplastic shapes differed, with fragments being more common in the surface waters (84% vs 16%), fragments and fibres approximately equally common in mid-column waters (55% vs 45%), and fibres most common in the seafloor sediment (37% vs 63%) (Fig. 4, Table S11).

Microplastic size distribution was comparable between surface and mid-column waters, with plastics of all size ranges detected in both compartments. Microplastics in size classes 3 (34% surface, 21% mid-column) and 5 (38% surface, 36% mid-column) were highest in abundance. Average (± SD) microplastic sizes were 1.23 ± 1.08 mm in surface waters and 1.21 ± 1.23 mm in mid-column waters, although, of note, plastics > 5 mm in length (size class 1) were also found, mostly in the mid-column. In contrast, the average microplastic size in sediment samples was smaller, at 0.72 ± 0.61 mm. No plastics were found belonging to size class 1, with most belonging to size classes 4 and 5 (33% and 46%, respectively).

Across all three matrices, the most abundant microplastic colour was blue, followed by pink and white in surface waters (26%, 25%, and 21%), transparent, black, and red in mid-column waters (43%, 17%, 10%, and 8%), and transparent and green in the sediment (37%, 21%, 10%). Other colours such as brown and orange were less common and usually represented less than 8% of colours within a matrix.

The occurrence of microplastic polymer type differed somewhat, with the less dense PP (35%) and PE (27%) particles most abundant in surface waters, PP (32%) and PET (29%) particles in mid-column waters, and the denser PET (38%) and nylon (13%) particles in the sediment. Other polymers such as acrylic, PS, and PVC were also recovered, but in much smaller quantities and not necessarily in all three abiotic matrices.

### Profile of microplastic contamination in biotic matrices and relationship to the environment

**Fig. 5:**
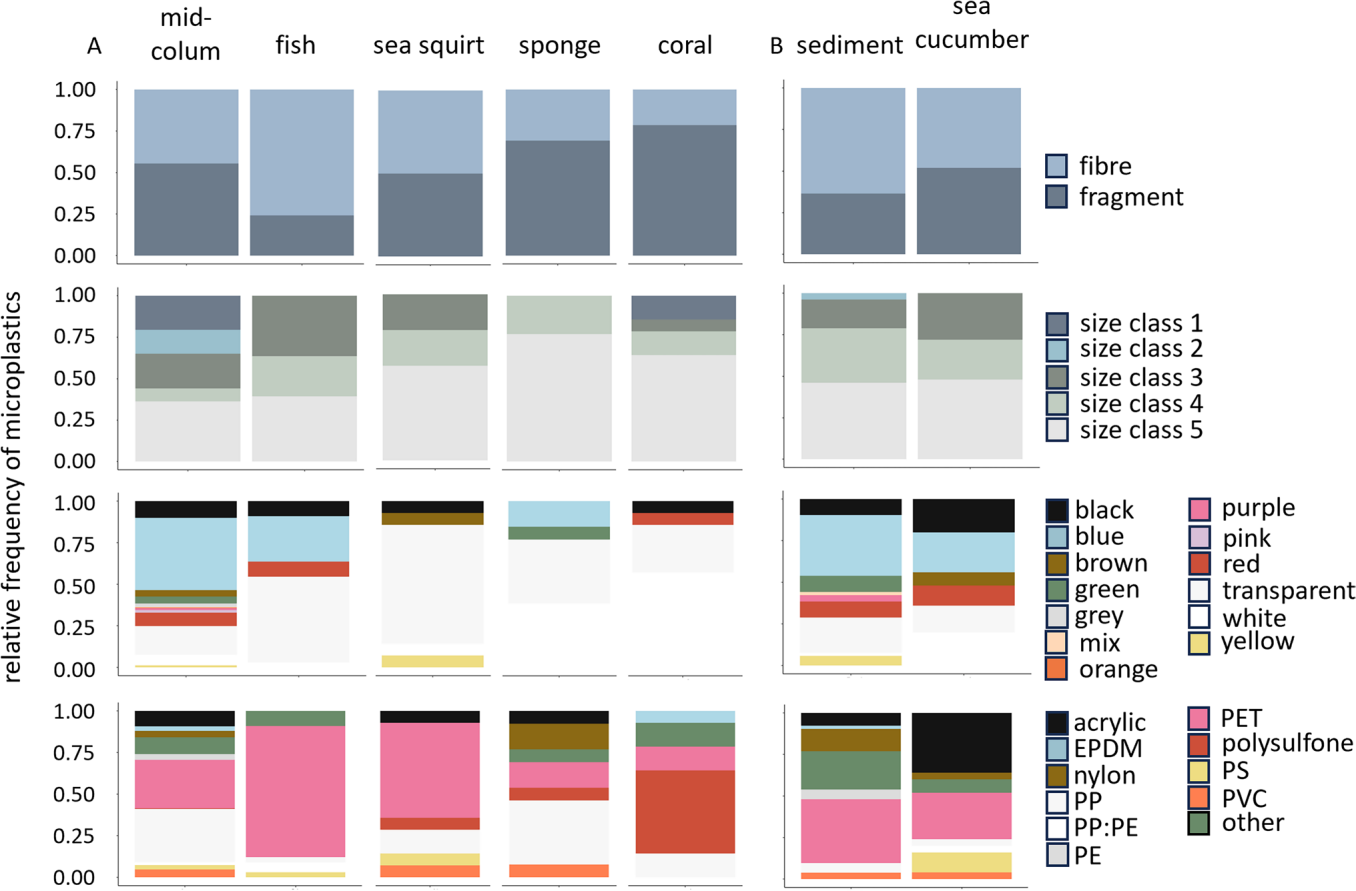
Relative number of microplastics found in biota from Lizard Island in comparison to their habitat. (**A**) The microplastic profiles of fish, sea squirt, sponge, and coral in relation to their local mid-column environment. (**B**) The microplastic profiles of sea cucumber in relation to their local sediment environment. Each row represents the distribution of a microplastic characteristic (shape, size, colour, and polymer) within each abiotic matrix and associated taxa

Overall, for all taxa, microplastic profiles in organisms reflected the abiotic compartment they inhabit (Fig. 5, Table S11). Bayesian logistic regression models exploring the relationship between microplastic characteristics and abiotic/biotic matrices did, however, reveal exceptions to this. Of note, convergence diagnostics indicated satisfactory mixing and chain convergence for all models, ensuring reliable inference with adequate effective sample sizes.

**Fig. 6:**
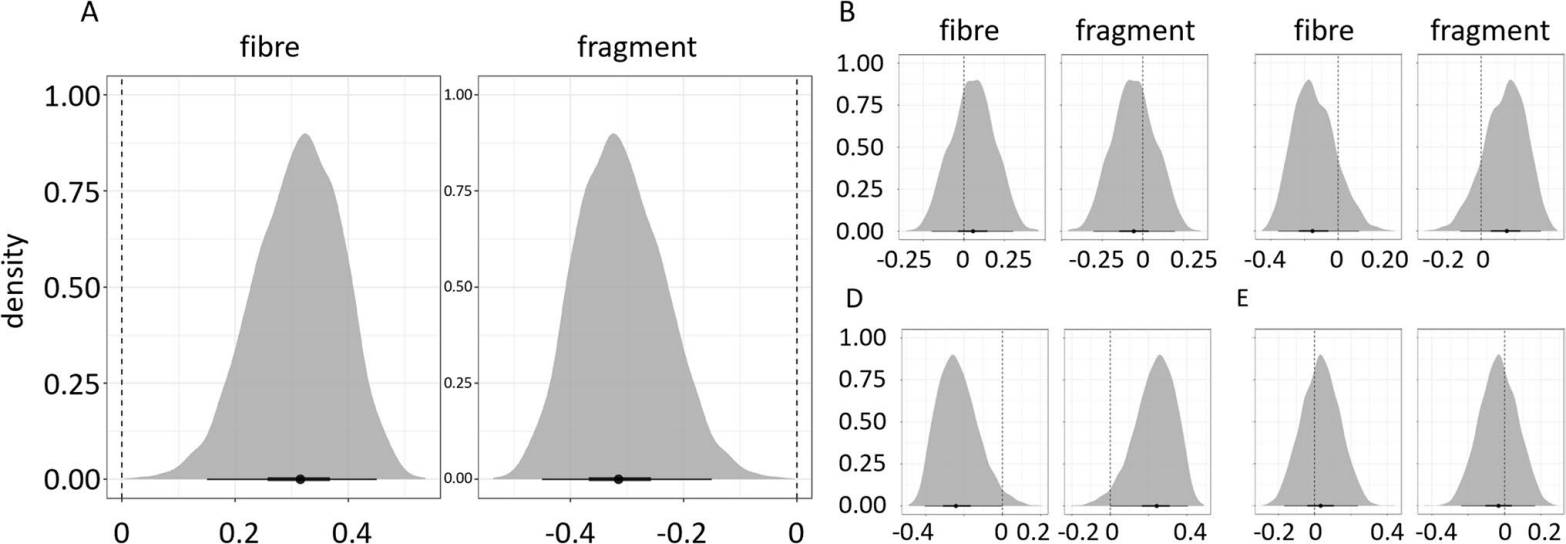
Bayesian posterior distributions illustrate the relationships between microplastic shape (fibre and fragment) in the mid-column water and fish (**A**), sea squirt (**B**), sponge (**C**), and coral (**D**), and in the seafloor sediment and sea cucumber (**E**). Black point represents the mean difference. Horizontal error bars are Bayesian 50% (thick) and 90% (thin) credible intervals. Absolute differences between abiotic and biotic compartments were expected to be centred around 0, which is indicated by the vertical dashed line

Bayesian analysis revealed fish had a higher ratio of fibres (76%) to fragments (24%) in comparison to the mid-column water ratios of fibres and fragments (Fig. 5, Table S11), with posterior mean differences of − 0.31 for fibres and 0.31 for fragments (95% HDCI, − 0.46 to − 0.16 and 0.16 to 0.46, respectively) (Fig. 6, Table S12). For sea squirt, sponge, and coral, considering Bayesian 50% credible intervals, the mean difference of the posterior distributions indicated different shape ratios between these organisms and the mid-column water (Tables S13a, S14a, S15a). However, the overlapping credible intervals including 0 suggested that these differences observed are not at 95% confidence; and therefore, there is no evidence to support differences between profiles found in sponge, coral, and sea squirt and their mid-column environment (e.g. 95% HDCI for sea squirt − 0.20 to 0.30 for fibres and − 0.30 to 0.20 for fragments, Tables S13b, S14b, S15b). Similarly, no differences were observed between microplastic shapes found in sea cucumbers and the seafloor sediment (Table S16).

The average microplastic size recovered from the examined organisms varied across all taxa, but items were generally small (fish 0.76 ± 0.48 mm, sea squirt 0.67 ± 0.60 mm, sponge 0.29 ± 0.25 mm, coral 0.41 ± 0.29 mm, sea cucumber 0.70 ± 0.48 mm). Plastics > 5 mm (size class 1) and large microplastics (5 – 2.5 mm, size class 2) were absent from all taxa except for corals (class 1, 15%) (Fig. 5, Table S11) despite being present in the water column (class 1, 21%; class 2, 14%). In contrast, microplastics < 500 µm were abundant in all species (fish 40%, sea squirt 58%, sponge 77%, coral 64%, sea cucumber 48%) while representing 36% and 46% of what were found in the mid-column water and sediment, respectively. Bayesian logistic regression models revealed the size distribution in organisms differed from the abiotic compartment they inhabit, with organisms having fewer microplastics from size classes 1 (> 5 mm) and 2 (5 – 2.5 mm), but similar (i.e. mean difference of the posterior distributions centred at 0 and/or overlapping credible intervals that included 0) or higher microplastic contents from size classes 3, 4, and 5 (Figure S9, Tables S17–S21).

Most microplastic colours were similarly represented in both abiotic and biotic compartments; however, there were exceptions. Fish and sea squirts contained more transparent microplastics (52% and 72%, respectively) compared to their mid-column environment (Fig. 5, Table S11), with posterior mean differences of − 0.35 for fish and − 0.54 for sea squirts (95% HDCI − 0.52 to − 0.17 and − 0.75 to − 0.28, respectively, Figure S10, Tables S22–23). Sponges and corals showed predominance of white items (38% and 57%, respectively) relative to their availability (7% in the mid-column) (Tables S24–25). There was a notable difference between the profiles of these four taxa and the mid-column, with respect to blue microplastics, with all four taxa containing fewer or no blue microplastics (fish 27%, sea squirt none, sponge 16%, coral none), despite a prevalence of blue items in the mid-column water (43%). Likewise, sponges did not contain any black or red items, yet these were present in the mid-column (10% and 8%, respectively). A higher percentage of white microplastics was present in sea cucumbers compared to the seafloor sediment (20% vs 2%), yet no green microplastics were found despite these being fivefold more abundant than white microplastics in the seafloor sediment (10%) (Table S26).

At least 50% of the polymer distribution in the abiotic matrices was similarly represented across the biotic compartments (Fig. 5, Table S11). Nonetheless, compared to their mid-column environment, higher levels of PET were recovered from fish (79% vs 29%) and sea squirt (57% vs 29%), PSU in corals (50% vs < 1%), and nylon in sponges (15% vs 3%) (Figure S11, Tables S27–30). Furthermore, the most prevalent polymers in the mid-column PP (32%) and PET (29%) were recovered in lesser quantities in three taxa (PP 3% in fish, 15% in sea squirts; PET 15% in sponges, 14% in corals), while other polymers were absent altogether (no acrylic in fish and coral). Conversely, sea cucumbers presented with higher levels of acrylic (36%) and PS (12%) than the seafloor sediment (8% and none, respectively) (acrylic posterior mean difference, − 0.29 and 95% HDCI, − 0.49 to − 0.10; PS posterior mean difference, − 0.12 and 95% HDCI, − 0.28 to − 0.02) (Table S31).

## Discussion

### Microplastic contamination of a coral reef ecosystem

The presence of plastics, including microplastics, in coral reef environments is of widespread concern although under-investigated (Biswas et al. 2024; Huang et al. 2021). Using published methodologies for sample processing and microplastic identification and characterisation, microplastics were found in all three abiotic matrices (i.e. surface and mid-column waters, and seafloor sediment) and in all five taxa (i.e. fish, sea squirt, sponge, coral, and sea cucumber) sampled from two fringing coral reefs at Lizard Island in Australia. This study extends the findings of previous microplastic studies conducted in the Great Barrier Reef World Heritage Area (GBRWHA) (Hall et al. 2015; Jensen et al. 2019; Kroon et al. 2018b; Miller et al. 2022a), and not only corroborates the ubiquitous nature of this suite of contaminants in marine environments but also reveals that microplastic contamination is prevalent and widespread in one of the more remote areas of the largest coral reef system in the world.

Microplastic concentrations in surface waters at Lizard Island (average of 0.21 microplastics m^−3^ at Granite Bluff and 0.06 microplastics m^−3^ at Blue Lagoon) were similar to those reported in other areas within the GBRWHA (Table S32) and at the Faafu atoll in the Indian Ocean (0.03 to 0.65 microplastics m^−3^) (Saliu et al. 2018). Globally, these concentrations are considered low as higher numbers (up to five orders of magnitude greater) of microplastics are found in places such as the Tuticorin and Vembar Island groups in the Gulf of Mannar, India (60,000 to 126,600 microplastics m^−3^) (Patterson et al. 2020), and in the Nansha Islands in the South China Sea (1250 to 3200 microplastics m^−3^) (Nie et al. 2019). This discrepancy in concentrations is likely due to differences in local factors, with Tuticorin and Vembar Island groups and the Nansha Islands being closer to higher levels of continental industrialisation, urbanisation, and other anthropogenic activities such as fishing (Nie et al. 2019; Patterson et al. 2020). Furthermore, environmental variables at the time of collection (e.g. local hydrodynamics, wind patterns, and weather events such as monsoons) could have also influenced the results (Cheung and Not 2023; Critchell et al. 2015; Mendrik et al. 2024; Nie et al. 2019; Zhang 2017); however, the studies did not report sufficient information to validate this influence. In contrast, the few studies that do report on microplastic contamination in mid-column waters and sediments in coral reefs as reviewed by Huang et al. (2021) vary significantly in the methodology applied, limiting any inter-study comparisons. For example, comparison with Gao et al. (2023) is not feasible due to differences in the mid-column collection method (this study: pump filtering at specific depths vs Gao et al. 2023: vertical tow net filtering along the water column), neither with Patti et al. (2020) as reporting units differ (this study: microplastics m^−3^ vs Patti et al. 2020: microplastics kg^−1^ sediment) and limited supporting information is available to allow for data transformation. Direct comparison was possible, however, with a Central GBRWHA study (Miller et al. 2022a) as similar collection, processing, and comprehensive data reporting methods were used. This comparison revealed higher concentrations in the mid-column water at Lizard Island (1.47 to 12.74 microplastics m^−3^ in central GBRWHA vs 34.67 microplastics m^−3^ at Granite Bluff and 22.67 at Blue Lagoon) but similar levels in the seafloor sediment, likely as a function of local hydrodynamics (Critchell et al. 2015; Mendrik et al. 2024) and vertical transport of particulate matter, which is often coupled with biological processes such as biofouling and marine snow (Kaiser et al. 2017; Li et al. 2023a; Meng et al. 2024). Lizard Island region might have stronger mid-column water currents, more sediment resuspension, and lower sinking rates for microplastics than the coral reefs of the Central GBRWHA examined by (Miller et al. 2022a). However, environmental data to confirm these hypotheses is currently lacking for this region. To gain a deeper understanding of the factors influencing vertical microplastic distribution in coral reef systems, including Lizard Island, the GBRWHA, and coral reefs globally, additional baseline studies focusing on mid-column waters and seafloor sediments are essential. These studies should include detailed investigations of local oceanographic conditions in the water column (e.g. currents and water stratification), sediment resuspension dynamics, and microplastic sinking rates to substantiate findings on vertical distribution.

The level of microplastic contamination detected in fish from Lizard Island (ranging from 1.47 microplastics g^–1^ at Granite Bluff to 0.44 at Blue Lagoon, with an average of 3.3 microplastics individual^−1^) was comparable to global levels (3.5 microplastics individual^−1^) (Wootton et al. 2021) and specifically that reported in other planktivorous fish (e.g. 1.22 microplastics individual^−1^) (Kalaiselvan et al. 2022), including for the same species from the central GBRWHA (4 microplastics individual^−1^) (Jensen et al. 2019). On the other hand, microplastic levels found in coral and sea cucumber (the first to be reported for the GBRWHA) differed from global levels. In corals, microplastic concentrations (0.03 to 0.11 microplastics g^–1^) were within the lower levels of those reported at Xisha Island (South China Sea, 0.02–1.3 microplastics g^–1^, Ding et al. 2019), while sea cucumbers had significantly lower contamination levels (0.01 to 0.02 microplastics g^–1^) than previously reported from Rambut Island (Indonesia, 2.34 microplastics g^−1^) (Wicaksono et al. 2021). The microplastic levels found in sea squirt and sponge provide the one of the first confirmed baseline information of microplastic contamination in these coral reef species; considering most studies that previously reported microplastic contamination in coral reef sponges did not use chemical techniques to validate microplastic assignment (Fallon and Freeman 2021; Girard et al. 2021; Krikech et al. 2023), apart from one (Soares et al. 2022). To our knowledge, no studies confirming wild microplastic contamination in sea squirts have been published. As filter feeders, sea squirts and sponges are highly susceptible to incidental ingestion of particulates present in the water, including microplastics, yet it is unclear as to the long-term health effects. The scarcity of studies on microplastic contamination in the four invertebrate taxa examined here, combined with their ubiquitous presence in coral reef systems and prevalence of microplastics in all of them, underscores the need for further research to uncover potential biological effects on these organisms and predict broader impacts on the coral reef ecosystems, both locally and globally. Findings here establish an important baseline for corals, sponges, sea squirts, and sea cucumbers of the GBRWHA and will inform and support comparisons with future studies.

Overall, these findings highlight the ubiquitous presence of microplastics in both abiotic and biotic components of the GBRWHA and provide a baseline for further studies addressing this environmental issue. However, there remains limited knowledge about the factors that influence microplastic distribution in reef waters and sediments, as well as their ecological impacts on reef organisms. Addressing these knowledge gaps is critical for informing future conservation strategies and environmental policies aimed at mitigating plastic pollution (Beaumont et al. 2019) and its impact on the provision of marine ecosystem services, particularly in globally significant coral reef ecosystems like the GBRWHA.

### Source, fate, and distribution of microplastics in the reef ecosystem

The 14-day hind- and forecast numerical simulations generated credible trajectories for the microplastics found at Lizard Island. Within the 100 × 600 km^2^ spatial domain modelled herein, microplastics’ sources and fates were primarily associated with northeast Australian marine and coastal environments, suggesting a temporary retention of microplastics in Lizard Island waters with minimal accumulation. However, more long-term field monitoring is needed to assess input rates of microplastics to Lizard Island and to identify whether the observed contamination is sporadic or chronically present in the region.

Hindcast models suggest that over 50% of microplastics at Lizard Island originate from nearby islands and the Australian mainland, with the Coral Sea contributing only 15%. These results align with previous findings indicating landmasses (e.g. islands) and complex hydrodynamics partially retain microplastics in coral reef ecosystems (Mantovanelli and Heron 2012; Roman et al. 2021). Furthermore, predicted microplastic origins from northeastern coastal regions of Australia imply wind patterns also drive these trajectories, particularly from October to March when the south-easterly trade winds relax and monsoon winds become active, increasing the likelihood of cross-shelf water displacement in the GBRWHA (Benthuysen et al. 2016; Schiller et al. 2015). This could explain why the Coral Sea and Pacific Ocean, major input sources of microplastics into the mid-shelf GBRWHA between April and July (Jensen et al. 2019), contribute minimally to Lizard Island’s microplastic load in October. Yet further seasonal sampling at Lizard Island and other regions of the GBRWHA is needed to quantify the impact of wind-driven water movements on microplastic distribution, including when extreme weather events such as storms occur, and the influence of local and diffuse sources. Regardless, microplastics originating from oceanic waters could also be derived from the South Equatorial Current and its jets, another transport pathway for floating items to the eastern and south-eastern regions of the Lizard Island group. Overall, microplastic contamination in the region seems to arise from local and international sources, highlighting the need for broader spatial and temporal monitoring coupled with environmental parameter correlations (Miller et al. 2022b) to comprehensively understand contamination sources.

The 14-day forecast models predicted west-northwest directional trajectories for microplastics sampled at Lizard Island, with > 50% of the items stranding on islands and coastal areas of the Australian mainland or (although less likely) PNG, rather than persisting in the Lizard Island region. This finding revealed that in the GBRWHA there is a strong influence of wind superimposed on the local hydrodynamic recirculation (Critchell et al. 2015; Critchell and Lambrechts 2016), lending further support to the hypothesis that wind conditions have a major influence on the transport (Critchell et al. 2019) and abundance (Miller et al. 2022b) of microplastics throughout this coral reef ecosystem. The forecast modelling also corroborates previous studies conducted in the southern and central GBRWHA that suggest microplastics predominately travel northwards (Jensen et al. 2019; Mantovanelli and Heron 2012). Results here further highlight the importance of monitoring microplastics alongside physicochemical parameters (Miller et al. 2022b), which in combination are both crucial to understanding contamination patterns and improving the accuracy of model predictions.

Here, higher levels of microplastics were found contaminating abiotic compartments at Granite Bluff and reinforce previous reports of high variability in microplastic contamination between coral reefs (Huang et al. 2021), even at localised scales. Granite Bluff is sheltered from the Southeast Trade wind between March and September, and this buffer might have contributed to the microplastic concentrations observed in October 2018, further corroborating the notion that wind conditions have a strong influence on the distribution of microplastics in marine environments. Other localised oceanographic processes such as the formation of island wakes (Wolanski and Spagnol 2000) or slicks (Gallardo et al. 2021) could also be influencing the results. These oceanographic features can entrap particles that will only be transported out and dispersed when at the edge of the system (Sandulescu et al. 2006) or when the feature dissipates. However, as wind is also a driving factor of such processes, this further reinforces the extent of influence wind has on the distribution of microplastics at each site. Although less likely, input from local human activities may have also contributed to the differences found between the two sites (Li et al. 2024b; Lin et al. 2024). Granite Bluff is located adjacent to Watson’s Bay which hosts a resort, a camp site, and anchorages for numerous recreational and commercial vessels, while Blue Lagoon is largely sanctioned for scientific research with minimal tourism. However, as beaches surrounding Granite Bluff generally contained lower amounts of plastic debris compared to those surrounding Blue Lagoon (http://amdi.tangaroablue.org/), and numerical simulations predicting only a temporary retention, Lizard Island itself is unlikely to be the primary source of the microplastics detected at these two sites.

Vertical profiling of microplastic contamination in the field is under-investigated despite being essential for a comprehensive understanding of microplastic distribution, fate, and ecological risks. At both Lizard Island sites, microplastic contamination followed a depth gradient with levels increasing with greater depth, i.e. surface < mid-column < sediment. This points to sediments on the seafloor acting as a sink for denser microplastics that do not disperse northward as predicted by the hydrodynamic model. Regardless, the observed vertical distribution is consistent with other shallow marine systems (Liu et al. 2020; Song et al. 2018), including in the GBRWHA (Miller et al. 2022a), albeit not always (Zobkov et al. 2019). Differences in vertical distribution of microplastics are primarily influenced by oceanographic processes (e.g. vertical mixing) (Chevalier et al. 2023; Song et al. 2018; Zhang et al. 2023), microplastic features (e.g. shape and polymer density) (Liu et al. 2020), and/or biological mechanisms (e.g. biofouling) (Liu et al. 2020; Song et al. 2018). Thus, to better understand patterns and drivers of microplastic depth distribution and the associated ecological risks to inhabiting organisms, more comprehensive and compartmentalised field sampling across the depth gradient is essential (Lenaker et al. 2019), ideally alongside other environmental parameters.

### Bioavailability of microplastics to coral reef organisms

In this study, despite the lack of evidence for the accumulation of microplastics in reef waters, all five taxa (fish, sea squirt, sponge, coral, and sea cucumber) were found to be contaminated with microplastics. This suggests widespread biological contamination might not exclusively occur in accumulation zones. Of the five taxa analysed, the level of microplastic contamination in fish was significantly higher than in most invertebrates which, compared to each other, were similarly contaminated. This difference is likely to be influenced by the feeding habits and strategies of the different taxa and the associated mode of microplastics intake (either inadvertently and unintentionally or intentionally). *Pomacentrus amboinensis* is a highly mobile omnivorous and opportunistic feeder (Bray 2018), consuming a diverse range of food items in the mid-column water, primarily not only plankton, algae, and small invertebrates, but also detritus. This omnivorous diet allows them to exploit a variety of food sources available in reef ecosystems, potentially contributing to the relatively high diversity (e.g. wider size range) of microplastics types they ingest (Ceylan et al. 2024). In contrast, the four invertebrate species have more specialised diets, tailored to their respective ecological niches, feeding mechanisms, and anatomies (Fallon and Freeman 2021; Iwalaye et al. 2020; Reichert et al. 2024; Vered et al. 2019), i.e. they are either filter feeders or rely on tentacles to capture passing prey. Furthermore, organism mobility positively impacts feeding rates as mobile taxa (e.g. fish) can move towards food and potentially microplastics (Li et al. 2024a; Savoca et al. 2021) while sedentary or sessile feeders (e.g. invertebrates tested here) are highly dependent on what is available in their immediate surroundings. Unfortunately, the scarcity of experimental and field-based studies that explicitly test these hypotheses means there is a critical need to validate these assumptions and establish the relationship between feeding behaviour and ingestion of microplastics. Assumptions should be also made with caution as the physical and chemical characteristics of the microplastic contamination can influence intake, i.e. some microplastics are actively ingested or avoided (Ory et al. 2017; Reichert et al. 2024; Roch et al. 2020), and depuration (Santana et al. 2021, refs). Thus, when assessing bioavailability, it is critical to report on all aspects of the contaminating microplastics.

The shape profile of microplastics ingested by fish did not mirror that of their surrounding environment. Even though both microplastic fibres and fragments were more or less equally present (45% vs 55%, respectively) in the mid-column, fish gastrointestinal tracts (GIT) contained more fibres (76%) and fewer fragments (24%), potentially indicating selective ingestion. Invertebrates, on the other hand, had a similar profile to the abiotic compartments they inhabited, indicative of random or accidental intake. The prevalence of fibre contamination in fish has been previously reported in other field studies (Filgueiras et al. 2020; Jensen et al. 2019; Kroon et al. 2018b; Lim et al. 2022), and specifically for *P. amboinensis* (Jensen et al. 2019) where > 99% of items found were fibres. Jensen et al. (2019) postulated fish were actively (i.e. selectively) ingesting microplastic fibres based on their similar appearance to natural food sources such as filamentous algae (Peters et al. 2017; Roch et al. 2020). However, recent studies (Santana et al. 2021; Xiong et al. 2019) have shown fish, including *P. amboinensis* (Santana et al. 2021), take longer to depurate microplastic fibres compared to fragments, and this may lead to fibre retention and a higher fibre loading. Establishing whether ingestion is selective, or if depuration is the major factor determining the nature of the microplastic contamination in fish, is therefore critical for accurate microplastics bioavailability and risk assessments.

Microplastics < 500 µm were highly abundant across all taxa while large microplastics (class 2, 2.5–5 mm) and plastics (class 1, > 5 mm) were almost absent. In fact, corals were the only taxon found contaminated with plastics > 5 mm and no organism was found to contain microplastics between 5 and 2.5 mm in length. This suggests smaller microplastics are highly bioavailable to these taxa, possibly mimicking their preferred food size and ability to be readily ingested by different anatomical feeding structures and modes. The prevalence of microplastics from size class 3 (2.5–1 mm) to 5 (< 500 µm) in the GIT of fish, and especially microplastics < 1 mm (64%), is supported by previous field (Avio et al. 2015; Su et al. 2019) and laboratory assessments (Critchell and Hoogenboom 2018; Xiong et al. 2019), with small-sized microplastics being more readily swallowed (Xiong et al. 2019) or having slower depuration rates (Liu et al. 2021), or both. For sea squirts, sponges, and corals, the majority of microplastics ingested were < 500 µm (58%, 77% and 64%, respectively). Sea squirts actively take in food and also microplastics < 400 µm (Vered et al. 2019) and selectively expel larger undesirable items > 1.3 mm (Tatian et al. 2004), while sponges are known to preferentially intake items < 100 µm via oscula (Reiswig 1971; Simpson 2012), although larger particles, including of microplastics, can be sporadically captured and engulfed via endocytosis (Fallon and Freeman 2021). Microplastics < 500 µm also fall within the size range of coral prey, which is usually captured via tentacles, and while this size range of microplastics is often reported contaminating corals (Hall et al. 2015; Hankins et al. 2018, 2022), they were also found to contain large microplastics and plastics. These could have been assimilated through tissue overgrowth mechanisms (Martin et al. 2019; Reichert et al. 2018). Lastly, and as previously reported, the microplastic size range in sea cucumbers most closely mimicked that of their surroundings and mainly comprised items up to 2.5 mm (Graham and Thompson 2009; Mohsen et al. 2019). Yet, microplastic size is likely to be dictated by the diameter of the mouth opening of these organisms (Mohsen et al. 2019). In agreement with the literature, our results emphasise the increased risk of contamination in biota with decreasing microplastics size, likely related to organismal food size, anatomy, and physiology.

The impact of colour on the bioavailability of microplastics is challenging to accurately assess given the limited research conducted in this area. *Pomacentrus amboinensis* is the only taxon examined that relies on vision (luminance UV) to discriminate form and texture, including when hunting (Siebeck 2016) and preference for certain microplastic colours has been suggested (Jensen et al. 2019), in line with that known for other fish species (Carlos de Sa et al. 2015; Ory et al. 2017; Santos et al. 2016). The high levels of transparent microplastics found in *P. amboinensis* also suggest a preference for translucent or transparent planktonic prey; however, this is contradictory to Jensen et al. (2019) who observed a higher intake of blue microplastics. Controlled laboratory exposures are needed to validate field observations and enable robust inferences on the role, if any, of *P. amboinensis* vision in microplastics intake, especially considering the influence of UV wavelengths on damselfish vision and food perception (among other things) (Siebeck 2016). Among the invertebrate taxa, sea squirts, sponges, and corals contained high levels of transparent and/or white microplastics compared to the mid-column and sea cucumber contained more white items than the sediment. These trends are likely due to characteristics other than colour given the lack of colour perception in these organisms (Picciani et al. 2021; Rivera et al. 2012).

The influence of polymer type on microplastics bioavailability is a topic of current debate (Li et al. 2023b) with only incipient and often conflicting information available. Because some organisms, such as fish and sea cucumber, are capable of selective ingestion based on olfaction (or chemosensation) (Kasumyan and Marusov 2016; van Dam-Bates et al. 2016), it is suspected that this could reduce the unintentional ingestion of microplastics (Roch et al. 2020). Yet, studies to confirm the influence of polymer chemistry on microplastic bioavailability face significant challenges, more so for field studies, due to the difficulty in controlling for the other characteristics (e.g. shape, size, colour, and density) and environmental factors influencing exposure (e.g. biofouling). For instance, while previous correlation between plastic abundance in water and contamination in fish implied incidental intake as a primary ingestion route (Savoca et al. 2021), here it fails to account for disparities observed, i.e. the higher prevalence of PET microplastics in Lizard Island fish compared to mid-column water (79% vs. 29%) and lower levels of PP (3% vs 32%, respectively). As for colour, it is likely that microplastic characteristics other than polymer type play a major role in the intake of microplastics. Here, the predominant shape of the PET found in fish was fibre (Figure S12), noting that fibres overall were more prevalent in fish in comparison to fragments. Similarly, sea squirt, coral, and sponges do not rely on vision or chemosensation for feeding indicating that shape and size are likely more important factors. Still, in field studies, environmental factors such as the formation of biofilms on microplastics can also influence the chemical signature of debris, and the chemistry emitted from their surface, with biofilm composition and rate of formation being a function of polymer type and time immersed (Agostini et al. 2021; Feng et al. 2020; Kirstein et al. 2019). Unfortunately, the sampling and processing methodologies used here (and commonly applied across similar studies) (Monteiro and Pinto da Costa 2022, Santana et al. 2022b, Sharma et al. 2024) are unsuited to preserving biofilms; hence, it is not appropriate to infer any influence. Furthermore, the absence of data on how polymer type affects retention and degradation rates of microplastics in digestive gut systems complicates the separation of these effects from physiological factors that may also affect the observed results. This lack of knowledge underscores the necessity for further research to elucidate the role of polymer type in microplastic-biological interactions.

## Conclusion

As global concerns about environmental microplastic contamination grow, establishing comprehensive baseline data on microplastic abundance and characteristics in abiotic and biotic matrices, coupled with numerical simulations to assess distribution and ecological risks, is essential for supporting environmental risk assessments (ERAs). This study provides valuable insights into microplastic contamination in the coral reefs of Lizard Island, GBRWHA. By revealing contamination across all examined matrices, it establishes key data to guide ERA development for this region.

Numerical hindcast modelling indicated that microplastics in Lizard Island predominantly originated locally, from plastics beached on nearby islands and the continent, influenced by seasonal winds. Numerical forecasting indicated northward transport, not only reaching the Australian coastline back but also travelling as far as Papua New Guinea. This suggests that the waters of Lizard Island are not accumulation zones but rather areas of temporary microplastic retention with continuous influx. To determine if this pattern is seasonal or persistent, further hydrodynamical modelling using field data across different seasons is recommended. Understanding these transport pathways is crucial not only for local contamination management but also for regional and global contaminant dynamics. In contrast, increased microplastic concentrations with depth suggest seafloor of coral reefs as a potential sink for microplastics, warranting targeted research on sinking rates, mid-column hydrodynamics, and vertical transport. Techniques such as sediment traps and particle tracking models in different reef systems globally could facilitate this investigation.

Statistical simulations revealed higher microplastic contamination in fish compared to invertebrates, reflecting taxa variability, and, potentially, influences of ecological traits such as feeding habits and mechanisms. Of the microplastics ingested, items in the smaller size range (< 500 µm) were particularly abundant for all taxa and fibres (~ 75%) for fish, reflecting their high ingestion potential or potential retention in organisms. These findings underline the need for urgent assessments of fish-microplastic interactions particularly, including the ecological risks of ingested microplastics of environmental relevance. Additionally, dissimilarities in shape, colour, and polymer type between abiotic and biotic compartments warrant laboratory-based studies to elucidate bioavailability mechanisms, such as visual and chemical ingestion cues, in both vertebrates and invertebrates.

Overall, this study has significantly expanded the limited in-field and numerical modelling research on microplastics in coral reef ecosystems, particularly in the GBRWHA. By integrating baseline data, transport dynamics, and taxa-specific vulnerabilities, this research advances microplastic-specific ERAs for coral reef ecosystems and highlights key directions for future investigations.

## Supplementary Information

Below is the link to the electronic supplementary material.

Supplementary file1 (DOCX 4598 KB)

## Data Availability

The authors declare that the data supporting the findings of this study are available within the paper and its Supplementary information files. Should any raw data files be needed in another format, they are available from the corresponding author upon request.

## References

[CR1] Agostini L, Moreira JCF, Bendia AG, Kmit MCP, Waters LG, Santana MFM, Sumida PYG, Turra A, Pellizari VH (2021) Deep-sea plastisphere: long-term colonization by plastic-associated bacterial and archaeal communities in the Southwest Atlantic Ocean. Sci Total Environ 793:148335 34174607 10.1016/j.scitotenv.2021.148335

[CR2] Amenabar M, Aguilera MA, Gallardo C, Moore C, De Vine R, Lattin G, Gamba A, Luna-Acosta A, Thiel M (2024) Spatial distribution of microplastics in a coastal upwelling region: offshore dispersal from urban sources in the Humboldt Current System. Environ Pollut 343:123157 38142808 10.1016/j.envpol.2023.123157

[CR3] Avio CG, Gorbi S, Regoli F (2015) Experimental development of a new protocol for extraction and characterization of microplastics in fish tissues: first observations in commercial species from Adriatic Sea. Mar Environ Res 111:18–26 26210759 10.1016/j.marenvres.2015.06.014

[CR4] Beaumont NJ, Aanesen M, Austen MC, Borger T, Clark JR, Cole M, Hooper T, Lindeque PK, Pascoe C, Wyles KJ (2019) Global ecological, social and economic impacts of marine plastic. Mar Pollut Bull 142:189–195 31232294 10.1016/j.marpolbul.2019.03.022

[CR5] Benthuysen JA, Tonin H, Brinkman R, Herzfeld M, Steinberg C (2016) Intrusive upwelling in the Central Great Barrier Reef. J Geophys Res: Oceans 121:8395–8416

[CR6] Biswas T, Pal SC, Saha A, Ruidas D, Shit M, Islam ARMT, Malafaia G (2024) Microplastics in the coral ecosystems: a threat which needs more global attention. Ocean Coast Manag 249:107012

[CR7] Bray DJ (2018) Pomacentrus amboinensis in fishes of Australia

[CR8] Carlos de Sa L, Luis LG, Guilhermino L (2015) Effects of microplastics on juveniles of the common goby (Pomatoschistus microps): confusion with prey, reduction of the predatory performance and efficiency, and possible influence of developmental conditions. Environ Pollut 196:359–362 25463733 10.1016/j.envpol.2014.10.026

[CR9] Ceylan L, Ari H, Erdogan S (2024) The role of habitat preference and feeding strategy on exposure to microplastic pollution in freshwater fish species. Chemosphere 370:143921 39653191 10.1016/j.chemosphere.2024.143921

[CR10] Cheung CKH, Not C (2023) Impacts of extreme weather events on microplastic distribution in coastal environments. Sci Total Environ 904:166723 37659554 10.1016/j.scitotenv.2023.166723

[CR11] Chevalier C, Vandenberghe M, Pagano M, Pellet I, Pinazo C, Tesan Onrubia JA, Guilloux L, Carlotti F (2023) Investigation of dynamic change in microplastics vertical distribution patterns: the seasonal effect on vertical distribution. Mar Pollut Bull 189:114674 36933288 10.1016/j.marpolbul.2023.114674

[CR12] Critchell K, Hoogenboom MO (2018) Effects of microplastic exposure on the body condition and behaviour of planktivorous reef fish (Acanthochromis polyacanthus). PLoS One 13:e0193308 29494635 10.1371/journal.pone.0193308 PMC5832226

[CR13] Critchell K, Lambrechts J (2016) Modelling accumulation of marine plastics in the coastal zone; what are the dominant physical processes? Estuar Coast Shelf Sci 171:111–122

[CR14] Critchell K, Grech A, Schlaefer J, Andutta FP, Lambrechts J, Wolanski E, Hamann M (2015) Modelling the fate of marine debris along a complex shoreline: lessons from the Great Barrier Reef. Estuar Coast Shelf Sci 167:414–426

[CR15] Critchell K, Hamann M, Wildermann N, Grech A (2019) Predicting the exposure of coastal species to plastic pollution in a complex island archipelago. Environ Pollut 252:982–991 31252137 10.1016/j.envpol.2019.06.031

[CR16] Dawson AL, Santana MFM, Nelis JLD, Motti CA (2023) Taking control of microplastics data: a comparison of control and blank data correction methods. J Hazard Mater 443:130218 36367473 10.1016/j.jhazmat.2022.130218

[CR17] Deltares DD-F (2016) Simulation of multi-dimensional hydrodynamic flows and transport phenomena, including sediments. In: User manual D, Delft (Hrsg.), The Netherlands

[CR18] Ding J, Jiang F, Li J, Wang Z, Sun C, Wang Z, Fu L, Ding NX, He C (2019) Microplastics in the coral reef systems from xisha islands of South China Sea. Environ Sci Technol 53:8036–8046 10.1021/acs.est.9b01452 31204475

[CR19] Environmental Protection Agency U (1992) Framework for ecological risk assessment, Washington, DC

[CR20] Everaert G, De Rijcke M, Lonneville B, Janssen CR, Backhaus T, Mees J, van Sebille E, Koelmans AA, Catarino AI, Vandegehuchte MB (2020) Risks of floating microplastic in the global ocean. Environ Pollut 267:115499 33254632 10.1016/j.envpol.2020.115499

[CR21] Fallon BR, Freeman CJ (2021) Plastics in Porifera: the occurrence of potential microplastics in marine sponges and seawater from Bocas del Toro. Panama Peerj 9:e11638 34285830 10.7717/peerj.11638 PMC8272925

[CR22] Feng L, He L, Jiang S, Chen J, Zhou C, Qian ZJ, Hong P, Sun S, Li C (2020) Investigating the composition and distribution of microplastics surface biofilms in coral areas. Chemosphere 252:126565 32220722 10.1016/j.chemosphere.2020.126565

[CR23] Filgueiras AV, Preciado I, Carton A, Gago J (2020) Microplastic ingestion by pelagic and benthic fish and diet composition: a case study in the NW Iberian shelf. Mar Pollut Bull 160:111623 32896713 10.1016/j.marpolbul.2020.111623

[CR24] Fraissinet S, Arduini D, Martines A, De Benedetto GE, Malitesta C, Giangrande A, Rossi S (2024) Seasonal occurrence and distribution of microplastics in four different benthic suspension feeders from an Integrated Multi-Trophic Aquaculture (IMTA) facility: a bioremediation perspective. Mar Pollut Bull 207:116811 39121801 10.1016/j.marpolbul.2024.116811

[CR25] Frith CA, Leis JM, Goldman B (1986) Currents in the Lizard Island region of the Great Barrier Reef Lagoon and their relevance to potential movements of larvae. Coral Reefs 5:81–92

[CR26] Gallardo C, Ory NC, Gallardo MdlÁ, Ramos M, Bravo L, Thiel M (2021) Sea-surface slicks and their effect on the concentration of plastics and zooplankton in the coastal waters of Rapa Nui (Easter Island). Front Mar Sci 8:688224

[CR27] Gao S, Yan K, Liang B, Shu R, Wang N, Zhang S (2023) The different ways microplastics from the water column and sediment accumulate in fish in Haizhou Bay. Sci Total Environ 854:158575 36075424 10.1016/j.scitotenv.2022.158575

[CR28] Gao C, Liang B, Zhang S (2024) Accumulation characteristics and ecological risk evaluation of microplastics in sediment cores from the artificial reef area and surrounding seas of Haizhou Bay, north China. Sci Total Environ 925:171789 38508275 10.1016/j.scitotenv.2024.171789

[CR29] GESAMP (2016) Sources, fate and effects of microplastics in the marine environment: part two of a global assessment

[CR30] Girard EB, Fuchs A, Kaliwoda M, Lasut M, Ploetz E, Schmahl WW, Worheide G (2021) Sponges as bioindicators for microparticulate pollutants? Environ Pollut 268:115851 33126031 10.1016/j.envpol.2020.115851

[CR31] Graham ER, Thompson JT (2009) Deposit- and suspension-feeding sea cucumbers (Echinodermata) ingest plastic fragments. J Exp Mar Biol Ecol 368:22–29

[CR32] Griffin D, Oke P, Jones E (2016) The search for MH370 and ocean surface drift. , CSIRO Australia

[CR33] Gunaalan K, Almeda R, Vianello A, Lorenz C, Iordachescu L, Papacharalampos K, Nielsen TG, Vollertsen J (2024) Does water column stratification influence the vertical distribution of microplastics? Environ Pollut 340:122865 37926412 10.1016/j.envpol.2023.122865

[CR34] Hall NM, Berry KLE, Rintoul L, Hoogenboom MO (2015) Microplastic ingestion by scleractinian corals. Mar Biol 162:725–732

[CR35] Hamylton SM, Leon JX, Saunders MI, Woodroffe CD (2014) Simulating reef response to sea-level rise at Lizard Island: a geospatial approach. Geomorphology 222:151–161

[CR36] Hankins C, Duffy A, Drisco K (2018) Scleractinian coral microplastic ingestion: potential calcification effects, size limits, and retention. Mar Pollut Bull 135:587–593 30301077 10.1016/j.marpolbul.2018.07.067 PMC6261434

[CR37] Hankins C, Raimondo S, Lasseigne D (2022) Microplastic ingestion by coral as a function of the interaction between calyx and microplastic size. Sci Total Environ 810:152333 34910947 10.1016/j.scitotenv.2021.152333 PMC8788577

[CR38] Hartmann NB, Huffer T, Thompson RC, Hassellov M, Verschoor A, Daugaard AE, Rist S, Karlsson T, Brennholt N, Cole M, Herrling MP, Hess MC, Ivleva NP, Lusher AL, Wagner M (2019) Are we speaking the same language? Recommendations for a definition and categorization framework for plastic debris. Environ Sci Technol 53:1039–1047 30608663 10.1021/acs.est.8b05297

[CR39] Herzfeld M, Andrewartha J, Baird M, Brinkman R, Furnas M, Gillibrand P, Hemer M, Joehnk K, Jones E, McKinnon D (2016) eReefs marine modelling

[CR40] Hidalgo-Ruz V, Gutow L, Thompson RC, Thiel M (2012) Microplastics in the marine environment: a review of the methods used for identification and quantification. Environ Sci Technol 46:3060–3075 22321064 10.1021/es2031505

[CR41] Huang W, Chen M, Song B, Deng J, Shen M, Chen Q, Zeng G, Liang J (2021) Microplastics in the coral reefs and their potential impacts on corals: a mini-review. Sci Total Environ 762:143112 33172634 10.1016/j.scitotenv.2020.143112

[CR42] Iwalaye OA, Moodley GK, Robertson-Andersson DV (2020) The possible routes of microplastics uptake in sea cucumber Holothuria cinerascens (Brandt, 1835). Environ Pollut 264:114644 32559857 10.1016/j.envpol.2020.114644

[CR43] Jensen LH, Motti CA, Garm AL, Tonin H, Kroon FJ (2019) Sources, distribution and fate of microfibres on the Great Barrier Reef, Australia. Sci Rep 9:9021 31227771 10.1038/s41598-019-45340-7 PMC6588688

[CR44] Jung JW, Park JW, Eo S, Choi J, Song YK, Cho Y, Hong SH, Shim WJ (2021) Ecological risk assessment of microplastics in coastal, shelf, and deep sea waters with a consideration of environmentally relevant size and shape. Environ Pollut 270:116217 33359873 10.1016/j.envpol.2020.116217

[CR45] Kaiser D, Kowalski N, Waniek JJ (2017) Effects of biofouling on the sinking behavior of microplastics. Environ Res Lett 12:124003

[CR46] Kalaiselvan K, Pandurangan P, Velu R, Robinson J (2022) Occurrence of microplastics in gastrointestinal tracts of planktivorous fish from the Thoothukudi region. Environ Sci Pollut Res Int 29:44723–44731 35137319 10.1007/s11356-022-19033-0

[CR47] Kasumyan AO, Marusov EA (2016) Selective feeding in fish: effect of feeding and defensive motivations evoked by natural odors. Biol Bull Rev 6:70–83 26201217

[CR48] Kirstein IV, Wichels A, Gullans E, Krohne G, Gerdts G (2019) The plastisphere - uncovering tightly attached plastic “specific” microorganisms. PLoS One 14:e0215859 31013334 10.1371/journal.pone.0215859 PMC6478340

[CR49] Krikech I, Oliveri Conti G, Pulvirenti E, Rapisarda P, Castrogiovanni M, Maisano M, Le Pennec G, Leermakers M, Ferrante M, Cappello T, Ezziyyani M (2023) Microplastics (</= 10 mum) bioaccumulation in marine sponges along the Moroccan Mediterranean coast: Insights into species-specific distribution and potential bioindication. Environ Res 235:116608 37429403 10.1016/j.envres.2023.116608

[CR50] Kroon F, Motti C, Talbot S, Sobral P, Puotinen M (2018a) A workflow for improving estimates of microplastic contamination in marine waters: a case study from North-Western Australia. Environ Pollut 238:26–38 29533881 10.1016/j.envpol.2018.03.010

[CR51] Kroon FJ, Motti CE, Jensen LH, Berry KLE (2018b) Classification of marine microdebris: a review and case study on fish from the Great Barrier Reef, Australia. Sci Rep 8:16422 30401888 10.1038/s41598-018-34590-6 PMC6219575

[CR52] Kuhn S, van Oyen A, Booth AM, Meijboom A, van Franeker JA (2018) Marine microplastic: preparation of relevant test materials for laboratory assessment of ecosystem impacts. Chemosphere 213:103–113 30216811 10.1016/j.chemosphere.2018.09.032

[CR53] Kvale K, Prowe AEF, Chien CT, Landolfi A, Oschlies A (2020) The global biological microplastic particle sink. Sci Rep 10:16670 33028852 10.1038/s41598-020-72898-4 PMC7542466

[CR54] La Beur L, Henry L-A, Kazanidis G, Hennige S, McDonald A, Shaver MP, Roberts JM (2019): Baseline assessment of marine litter and microplastic ingestion by cold-water coral reef benthos at the East Mingulay marine protected area (Sea of the Hebrides, Western Scotland). Front Mar Sci 6. 10.3389/fmars.2019.00080

[CR55] Lassig BR (1983) The effects of a cyclonic storm on coral reef fish assemblages. Environ Biol Fishes 9:55–63

[CR56] Lefebvre C, Cormier B, Le Bihanic F, Rampazzo Magalhaes G, Morin B, Lecomte S, Cachot J (2024) Temporal distribution of microplastics and other anthropogenic particles in four marine species from the Atlantic coast (France). Environ Pollut 357:124440 38936792 10.1016/j.envpol.2024.124440

[CR57] Lenaker PL, Baldwin AK, Corsi SR, Mason SA, Reneau PC, Scott JW (2019) Vertical distribution of microplastics in the water column and surficial sediment from the Milwaukee River basin to Lake Michigan. Environ Sci Technol 53:12227–12237 31618011 10.1021/acs.est.9b03850

[CR58] Li J, Shan E, Zhao J, Teng J, Wang Q (2023a) The factors influencing the vertical transport of microplastics in marine environment: a review. Sci Total Environ 870:161893 36731545 10.1016/j.scitotenv.2023.161893

[CR59] Li X, Kong Y, Juhasz AL, Zhou P, Zhang Q, Cui X (2023b) Effect of microplastic types on the in vivo bioavailability of polychlorinated biphenyls. Environ Sci Technol 57:12838–12846 37587565 10.1021/acs.est.3c04068

[CR60] Li B, Liang W, Fu S, Fu C, Cai Z, Munson A, Shi H (2024a) Swimming behavior affects ingestion of microplastics by fish. Aquat Toxicol 266:106798 38104508 10.1016/j.aquatox.2023.106798

[CR61] Li N, Wang X, Li X, Yi S, Guo Y, Wu N, Lin H, Zhong B, Wu WM, He Y (2024b) Anthropogenic and biological activities elevate microplastics pollution in headwater ecosystem of Yangtze tributaries in Hindu Kush-Himalayan region. J Hazard Mater 471:134395 38663293 10.1016/j.jhazmat.2024.134395

[CR62] Lim KP, Lim PE, Yusoff S, Sun C, Ding J, Loh KH (2022) A meta-analysis of the characterisations of plastic ingested by fish globally. Toxics 10:186 35448447 10.3390/toxics10040186 PMC9027263

[CR63] Lin J, Zhao Y-M, Zhan Z-G, Zheng J-Y, Zhou Q-Z, Peng J, Li Y, Xiao X, Wang J-H (2024) Microplastics in remote coral reef environments of the Xisha Islands in the South China Sea: source, accumulation and potential risk. J Hazard Mater 469:133872 38447364 10.1016/j.jhazmat.2024.133872

[CR64] Liu K, Courtene-Jones W, Wang X, Song Z, Wei N, Li D (2020) Elucidating the vertical transport of microplastics in the water column: a review of sampling methodologies and distributions. Water Res 186:116403 32932095 10.1016/j.watres.2020.116403

[CR65] Liu Y, Qiu X, Xu X, Takai Y, Ogawa H, Shimasaki Y, Oshima Y (2021) Uptake and depuration kinetics of microplastics with different polymer types and particle sizes in Japanese medaka (Oryzias latipes). Ecotoxicol Environ Saf 212:112007 33540337 10.1016/j.ecoenv.2021.112007

[CR66] Lusher AL, Munno K, Hermabessiere L, Carr S (2020) Isolation and extraction of microplastics from environmental samples: an evaluation of practical approaches and recommendations for further harmonization. Appl Spectrosc 74:1049–1065 32893667 10.1177/0003702820938993

[CR67] Madin JS, Baird AH, Bridge TCL, Connolly SR, Zawada KJA, Dornelas M (2018) Cumulative effects of cyclones and bleaching on coral cover and species richness at Lizard Island. Mar Ecol Prog Ser 604:263–268

[CR68] Mantovanelli A, Heron M (2012) Radar-based tracking of pollutants/larvae in the Coral Sea, 12th International Coral Reef Symposium, Cairns, Australia, 9–13 July 2012, Cairns, Australia, pp 5

[CR69] Manullang CY, Patria MP, Haryono A, Anuar ST, Fadli M, Susanto RD, Wei Z (2024) Vertical distribution of microplastic along the main gate of Indonesian Throughflow pathways. Mar Pollut Bull 199:115954 38176160 10.1016/j.marpolbul.2023.115954

[CR70] Martin C, Corona E, Mahadik GA, Duarte CM (2019) Adhesion to coral surface as a potential sink for marine microplastics. Environ Pollut 255:113281 31600700 10.1016/j.envpol.2019.113281

[CR71] Mendrik F, Houseago RC, Hackney CR, Parsons DR (2024) Microplastic trapping efficiency and hydrodynamics in model coral reefs: a physical experimental investigation. Environ Pollut 342:123094 38072017 10.1016/j.envpol.2023.123094

[CR72] Meng L, Sun X, Li Q, Zheng S, Liang J, Zhao C (2024) Quantification of the vertical transport of microplastics by biodeposition of typical mariculture filter-feeding organisms. Sci Total Environ 908:168226 37923264 10.1016/j.scitotenv.2023.168226

[CR73] Miller ME, Motti CA, Hamann M, Kroon FJ (2022a) Assessment of microplastic bioconcentration, bioaccumulation and biomagnification in a simple coral reef food web. Sci Total Environ 858:159615 36309288 10.1016/j.scitotenv.2022.159615

[CR74] Miller ME, Santana MFM, Carsique M, Motti CA, Hamann M, Kroon FJ (2022b) Temporal patterns of plastic contamination in surface waters at the SS Yongala shipwreck, Great Barrier Reef, Australia. Environ Pollut 307:119545 35643289 10.1016/j.envpol.2022.119545

[CR75] Mohsen M, Wang Q, Zhang L, Sun L, Lin C, Yang H (2019) Microplastic ingestion by the farmed sea cucumber Apostichopus japonicus in China. Environ Pollut 245:1071–1078 30682741 10.1016/j.envpol.2018.11.083

[CR76] Monteiro SS, Pinto da Costa J (2022) Methods for the extraction of microplastics in complex solid, water and biota samples. Trends Environ Anal Chem 33:e00151

[CR77] Moodley T, Abunama T, Kumari S, Amoah D, Seyam M (2024) Applications of mathematical modelling for assessing microplastic transport and fate in water environments: a comparative review. Environ Monit Assess 196:667 38935176 10.1007/s10661-024-12731-x PMC11211188

[CR78] Nie H, Wang J, Xu K, Huang Y, Yan M (2019) Microplastic pollution in water and fish samples around Nanxun Reef in Nansha Islands, South China Sea. Sci Total Environ 696:134022 31470325 10.1016/j.scitotenv.2019.134022

[CR79] Ory NC, Sobral P, Ferreira JL, Thiel M (2017) Amberstripe scad Decapterus muroadsi (Carangidae) fish ingest blue microplastics resembling their copepod prey along the coast of Rapa Nui (Easter Island) in the South Pacific subtropical gyre. Sci Total Environ 586:430–437 28196756 10.1016/j.scitotenv.2017.01.175

[CR80] Ourmieres Y, Arnaud M, Deixonne P, Ghiglione JF, Albignac M, Poulain-Zarcos M, Mercier M, Ter Halle A (2023) Inferring microplastics origins in the Mediterranean Sea by coupling modelling and in-situ measurements. Mar Pollut Bull 195:115333 37659382 10.1016/j.marpolbul.2023.115333

[CR81] Pastorino P, Barceló D (2024) Bioindicators selection in the strategies for monitoring microplastic pollution. Ecol Indic 166:112337

[CR82] Patterson J, Jeyasanta KI, Sathish N, Edward JKP, Booth AM (2020) Microplastic and heavy metal distributions in an Indian coral reef ecosystem. Sci Total Environ 744:140706 32711304 10.1016/j.scitotenv.2020.140706

[CR83] Patti TB, Fobert EK, Reeves SE, Burke da Silva K (2020) Spatial distribution of microplastics around an inhabited coral island in the Maldives, Indian Ocean. Sci Total Environ 748:141263 32814286 10.1016/j.scitotenv.2020.141263

[CR84] Peters CA, Thomas PA, Rieper KB, Bratton SP (2017) Foraging preferences influence microplastic ingestion by six marine fish species from the Texas Gulf Coast. Mar Pollut Bull 124:82–88 28705629 10.1016/j.marpolbul.2017.06.080

[CR85] Philipps CJ, Bellwood DR (2024) The hydrodynamics of Lizard Island lagoon, Great Barrier Reef. Coral Reefs 43:881–897

[CR86] Picciani N, Kerlin JR, Jindrich K, Hensley NM, Gold DA, Oakley TH (2021) Light modulated cnidocyte discharge predates the origins of eyes in Cnidaria. Ecol Evol 11:3933–3940 33976785 10.1002/ece3.7280 PMC8093662

[CR87] Portz L, Manzolli RP, Herrera GV, Garcia LL, Villate DA, Ivar do Sul JA (2020) Marine litter arrived: distribution and potential sources on an unpopulated atoll in the Seaflower Biosphere Reserve, Caribbean Sea. Mar Pollut Bull 157:111323 32658688 10.1016/j.marpolbul.2020.111323

[CR88] Potemra JT (2012) Numerical modeling with application to tracking marine debris. Mar Pollut Bull 65:42–50 21783212 10.1016/j.marpolbul.2011.06.026

[CR89] Pramanik BK, Pramanik SK, Monira S (2021) Understanding the fragmentation of microplastics into nano-plastics and removal of nano/microplastics from wastewater using membrane, air flotation and nano-ferrofluid processes. Chemosphere 282:131053 34098311 10.1016/j.chemosphere.2021.131053

[CR90] Prata JC, Castro JL, da Costa JP, Duarte AC, Rocha-Santos T, Cerqueira M (2020) The importance of contamination control in airborne fibers and microplastic sampling: experiences from indoor and outdoor air sampling in Aveiro, Portugal. Mar Pollut Bull 159:111522 32771665 10.1016/j.marpolbul.2020.111522

[CR91] Rahman MN, Shozib SH, Akter MY, Islam A, Islam MS, Sohel MS, Kamaraj C, Rakib MRJ, Idris AM, Sarker A, Malafaia G (2023) Microplastic as an invisible threat to the coral reefs: sources, toxicity mechanisms, policy intervention, and the way forward. J Hazard Mater 454:131522 37146332 10.1016/j.jhazmat.2023.131522

[CR92] Rathinamoorthy R, Balasaraswathi SR (2024) Microfibre pollution from textiles. microfibre pollution from textiles: research advances and mitigation strategies. The Textile Institute, pp 285

[CR93] Reichert J, Schellenberg J, Schubert P, Wilke T (2018) Responses of reef building corals to microplastic exposure. Environ Pollut 237:955–960 29146203 10.1016/j.envpol.2017.11.006

[CR94] Reichert J, Arnold AL, Hammer N, Miller IB, Rades M, Schubert P, Ziegler M, Wilke T (2022) Reef-building corals act as long-term sink for microplastic. Glob Chang Biol 28:33–45 34710272 10.1111/gcb.15920

[CR95] Reichert J, Tirpitz V, Oponczewski M, Lin C, Franke N, Ziegler M, Wilke T (2024) Feeding responses of reef-building corals provide species- and concentration-dependent risk assessment of microplastic. Sci Total Environ 913:169485 38143004 10.1016/j.scitotenv.2023.169485

[CR96] Reiswig HM (1971) In situ pumping activities of tropical Demospongiae. Mar Biol 9:38–50

[CR97] Rivera AS, Ozturk N, Fahey B, Plachetzki DC, Degnan BM, Sancar A, Oakley TH (2012) Blue-light-receptive cryptochrome is expressed in a sponge eye lacking neurons and opsin. J Exp Biol 215:1278–1286 22442365 10.1242/jeb.067140 PMC3309880

[CR98] Roch S, Friedrich C, Brinker A (2020) Uptake routes of microplastics in fishes: practical and theoretical approaches to test existing theories. Sci Rep 10:3896 32127589 10.1038/s41598-020-60630-1 PMC7054251

[CR99] Rochman CM, Brookson C, Bikker J, Djuric N, Earn A, Bucci K, Athey S, Huntington A, McIlwraith H, Munno K, De Frond H, Kolomijeca A, Erdle L, Grbic J, Bayoumi M, Borrelle SB, Wu T, Santoro S, Werbowski LM, Zhu X, Giles RK, Hamilton BM, Thaysen C, Kaura A, Klasios N, Ead L, Kim J, Sherlock C, Ho A, Hung C (2019) Rethinking microplastics as a diverse contaminant suite. Environ Toxicol Chem 38:703–711 30909321 10.1002/etc.4371

[CR100] Roman L, Warmbrunn A, Lawson TJ, Willis K, Wilcox C, Hardesty BD (2021) Comparing marine anthropogenic debris on inhabited mainland beaches, coastal islands, and uninhabited offshore islands: a case study from Queensland and the Coral Sea, Australia. Mar Pollut Bull 172:112919 34706475 10.1016/j.marpolbul.2021.112919

[CR101] Rotjan RD, Sharp KH, Gauthier AE, Yelton R, Lopez EMB, Carilli J, Kagan JC, Urban-Rich J (2019) Patterns, dynamics and consequences of microplastic ingestion by the temperate coral, Astrangia poculata. Proc Biol Sci 286:20190726 31238843 10.1098/rspb.2019.0726 PMC6599985

[CR102] Sacco VA, Zuanazzi NR, Selinger A, Alliprandini da Costa JH, Spanhol Lemunie E, Comelli CL, Abilhoa V, Sousa FC, Favaro LF, Rios Mendoza LM, de Castilhos GN, Delariva RL (2024) What are the global patterns of microplastic ingestion by fish? A scientometric review. Environ Pollut 350:123972 38642794 10.1016/j.envpol.2024.123972

[CR103] Saliu F, Montano S, Garavaglia MG, Lasagni M, Seveso D, Galli P (2018) Microplastic and charred microplastic in the Faafu Atoll, Maldives. Mar Pollut Bull 136:464–471 30509830 10.1016/j.marpolbul.2018.09.023

[CR104] Sandulescu M, Hernández-García E, López C, Feudel U (2006) Kinematic studies of transport across an island wake, with application to the Canary Islands. Tellus A: Dyn Meteorol Oceanogr 58:605–615

[CR105] Santana MFM, Dawson AL, Motti CA, van Herwerden L, Lefevre C, Kroon FJ (2021) Ingestion and depuration of microplastics by a planktivorous coral reef fish, Pomacentrus amboinensis. Front Environm Sci 9:641135

[CR106] Santana MFM, Kroon FJ, van Herwerden L, Vamvounis G, Motti CA (2022b) An assessment workflow to recover microplastics from complex biological matrices. Mar Pollut Bull 179:113676 35500374 10.1016/j.marpolbul.2022.113676

[CR107] Santana MFM, Blair L, Jaworski S, Motti CA (2022a) A validated method to quantify microplastic contamination in subsurface seawater: a case study sampling the Sydney nearshore under sail. Aust Inst Mar Sci

[CR108] Santos RG, Andrades R, Fardim LM, Martins AS (2016) Marine debris ingestion and Thayer’s law - the importance of plastic color. Environ Pollut 214:585–588 27131818 10.1016/j.envpol.2016.04.024

[CR109] Savoca MS, McInturf AG, Hazen EL (2021) Plastic ingestion by marine fish is widespread and increasing. Glob Chang Biol 27:2188–2199 33561314 10.1111/gcb.15533 PMC8247990

[CR110] Schiller A, Herzfeld M, Brinkman R, Stuart G (2014) Monitoring, predicting, and managing one of the seven natural wonders of the world. Bull Am Meteor Soc 95:23–30

[CR111] Schiller A, Herzfeld M, Brinkman R, Rizwi F, Andrewartha J (2015) Cross-shelf exchanges between the Coral Sea and the Great Barrier Reef lagoon determined from a regional-scale numerical model. Cont Shelf Res 109:150–163

[CR112] Schlawinsky M, Santana MFM, Motti CA, Martins AB, Thomas-Hall P, Miller ME, Lefèvre C, Kroon FJ (2022) Improved microplastic processing from complex biological samples using a customized vacuum filtration apparatus. Limnol Oceanogr Methods 20:553–567

[CR113] Sharma P, Sharma P, Abhishek K (2024) Sampling, separation, and characterization methodology for quantification of microplastic from the environment. J Hazard Mater Adv 14:100416

[CR114] Shaw KR, Whitney JL, Nalley EM, Schmidbauer MC, Donahue MJ, Black J, Corniuk RN, Teague K, Sandquist R, Pirkle C, Dacks R, Sudnovsky M, Lynch JM (2024) Microplastics absent from reef fish in the Marshall Islands: multistage screening methods reduced false positives. Mar Pollut Bull 198:115820 38029668 10.1016/j.marpolbul.2023.115820

[CR115] Siebeck UE (2016) Vision and colour diversity in damselfishes. In: Frederich B, Parmentier E (eds) Biology of damselfishes. Taylor & Francis Group, Boca Raton, p 28

[CR116] Simpson TL (2012) Functional morphology and morphological variation. In: Simpson TL (ed) The cell biology of sponges. Springer, New York, pp 1–41

[CR117] Soares GM, Barros F, Lanna E, da Silva MVS, Cavalcanti FF (2022) Sponges as libraries: increase in microplastics in Cinachyrella alloclada after 36 years. Mar Pollut Bull 185:114339 36395712 10.1016/j.marpolbul.2022.114339

[CR118] Soares MO, Rizzo L, Ximenes Neto AR, Barros Y, Martinelli Filho JE, Giarrizzo T, Rabelo EF (2023) Do coral reefs act as sinks for microplastics? Environ Pollut 337:122509 37690465 10.1016/j.envpol.2023.122509

[CR119] Sobha TR, Vibija CP, Fahima P (2023): Coral reef: a hot spot of marine biodiversity. In: Springer (Editor), Conservation and sustainable utilization of bioresources. Sustainable Development and Biodiversity, Singapore

[CR120] Song YK, Hong SH, Eo S, Jang M, Han GM, Isobe A, Shim WJ (2018) Horizontal and vertical distribution of microplastics in Korean coastal waters. Environ Sci Technol 52:12188–12197 30295469 10.1021/acs.est.8b04032

[CR121] Stark M (2019) Letter to the editor regarding “Are we speaking the same language? Recommendations for a definition and categorization framework for plastic debris.” Environ Sci Technol 53:4677 31021604 10.1021/acs.est.9b01360

[CR122] Su L, Nan B, Hassell KL, Craig NJ, Pettigrove V (2019) Microplastics biomonitoring in Australian urban wetlands using a common noxious fish (Gambusia holbrooki). Chemosphere 228:65–74 31022621 10.1016/j.chemosphere.2019.04.114

[CR123] Tan F, Yang H, Xu X, Fang Z, Xu H, Shi Q, Zhang X, Wang G, Lin L, Zhou S, Huang L, Li H (2020) Microplastic pollution around remote uninhabited coral reefs of Nansha Islands, South China Sea. Sci Total Environ 725:138383 32283309 10.1016/j.scitotenv.2020.138383

[CR124] Tatian M, Sahade R, Esnal GB (2004) Diet components in the food of Antarctic ascidians living at low levels of primary production. Antarct Sci 16:123–128

[CR125] Uddin S, Fowler SW, Uddin MF, Behbehani M, Naji A (2021) A review of microplastic distribution in sediment profiles. Mar Pollut Bull 163:111973 33484991 10.1016/j.marpolbul.2021.111973

[CR126] United Nations Environment Programme U (2021) From pollution to solution: a global assessment of marine litter and plastic pollution. , UNEP, Nairobi

[CR127] van Utenhove, EJF (2019) Modelling the transport and fate of buoyant macroplastics in coastal waters. Delft University of Technology. p 72

[CR128] van Dam-Bates P, Curtis DL, Cowen LLE, Cross SF, Pearce CM (2016) Assessing movement of the California sea cucumber Parastichopus californicus in response to organically enriched areas typical of aquaculture sites. Aquac Environ Interact 8:67–76

[CR129] van Sebille E, Wilcox C, Lebreton L, Maximenko N, Hardesty BD, van Franeker JA, Eriksen M, Siegel D, Galgani F, Law KL (2015) A global inventory of small floating plastic debris. Environ Res Lett 10:124006

[CR130] Vega-Moreno D, Sicilia-González S, Domínguez-Hernández C, Moreira-García E, Aguiar-González B, Hernández-Borges J, Fraile-Nuez E, Machín F (2024) Exploring the origin and fate of surface and sub-surface marine microplastics in the Canary Islands region. Front Mar Sci 11:1314754

[CR131] Vered G, Kaplan A, Avisar D, Shenkar N (2019) Using solitary ascidians to assess microplastic and phthalate plasticizers pollution among marine biota: a case study of the Eastern Mediterranean and Red Sea. Mar Pollut Bull 138:618–625 30660313 10.1016/j.marpolbul.2018.12.013

[CR132] Wicaksono KB, Patria MP, Suryanda A (2021) Microplastic ingestion in the black sea cucumber Holothuria leucospilota (Brandt, 1835) collected from Rambut Island, Seribu Islands, Jakarta, Indonesia. IOP Conf Ser: Mater Sci Eng 1098:052049

[CR133] Williams JJ, Esteves LS (2017) Guidance on setup, calibration, and validation of hydrodynamic, wave, and sediment models for shelf seas and estuaries. Adv Civ Eng 2017:1–25

[CR134] Wolanski E, Spagnol S (2000) Sticky waters in the Great Barrier Reef. Estuar Coast Shelf Sci 50:27–32

[CR135] Woodall LC, Sanchez-Vidal A, Canals M, Paterson GL, Coppock R, Sleight V, Calafat A, Rogers AD, Narayanaswamy BE, Thompson RC (2014) The deep sea is a major sink for microplastic debris. R Soc Open Sci 1:140317 26064573 10.1098/rsos.140317 PMC4448771

[CR136] Wootton N, Ferreira M, Reis-Santos P, Gillanders BM (2021) A comparison of microplastic in fish from Australia and Fiji. Front Mar Sci 8:690991

[CR137] Wootton N, Gillanders BM, Leterme S, Noble W, Wilson SP, Blewitt M, Swearer SE, Reis-Santos P (2024) Research priorities on microplastics in marine and coastal environments: an Australian perspective to advance global action. Mar Pollut Bull 205:116660 38981192 10.1016/j.marpolbul.2024.116660

[CR138] Xiong X, Tu Y, Chen X, Jiang X, Shi H, Wu C, Elser JJ (2019) Ingestion and egestion of polyethylene microplastics by goldfish (Carassius auratus): influence of color and morphological features. Heliyon 5:e03063 32083206 10.1016/j.heliyon.2019.e03063 PMC7019107

[CR139] Zelenke B, O'Connor CC, Barker CJ, Beegle-Krause, Eclipse L (2012) General NOAA Operational Modeling Environment (GNOME) technical documentation, NOS OR&R 40, NOAA’s Office of Response and Restoration (OR&R), Seattle, Washington

[CR140] Zhang H (2017) Transport of microplastics in coastal seas. Estuar Coast Shelf Sci 199:74–86

[CR141] Zhang M, Xu D, Liu L, Wei Y, Gao B (2023) Vertical differentiation of microplastics influenced by thermal stratification in a deep reservoir. Environ Sci Technol 57:6999–7008 37083351 10.1021/acs.est.2c09448

[CR142] Zobkov MB, Esiukova EE, Zyubin AY, Samusev IG (2019) Microplastic content variation in water column: the observations employing a novel sampling tool in stratified Baltic Sea. Mar Pollut Bull 138:193–205 30660263 10.1016/j.marpolbul.2018.11.047

