# Supplementary material for: Predicting microplastic dynamics in coral reefs: presence, distribution, and bioavailability through field data and numerical simulation analysis
Source: Environ Sci Pollut Res Int. 2025 Mar 26;32(15):9655–75. doi: 10.1007/s11356-025-36234-5 (PMC11991954; doi:10.1007/s11356-025-36234-5)
Supplement: Supplementary file 1 — Supplementary file1 (DOCX 4598 KB) [file 11356_2025_36234_MOESM1_ESM.docx]

**Predicting Microplastic Dynamics in Coral Reefs: presence, distribution and bioavailability through field data and numerical simulation analysis**

Marina F. M. Santana ^a,b,c^; Tonin, H. ^b^; Cherie A. Motti ^b,c^; Lynne van Herwerden ^a,c^; George Vamvounis ^a,c^; Frederieke J. Kroon ^b,c^.

Supplementary Methods

Sample Collection

Water samples

At both sites, surface tows (10 min) and mid-column pumping (20 min) were conducted in waters immediately adjacent to the fringing coral reefs (Table S1). Sea surface tows were conducted following standardized methods (Kroon et al. 2018a, Wootton et al. 2024). A 355 µm aperture plankton net (254 cm length) attached to a neuston frame (74.5 diameter x 30.0 cm height) was deployed port side and towed at ~4 knots to ensure it was half-immersed in the water along its long axis. Boat speed and start and end GPS coordinates (Garmin GPS76) were recorded, and the volume of water filtered calculated by multiplying the tow distance by half the area of the neuston frame (Kroon et al. 2018a). The 355 µm net aperture size defines the lower size category of collected microplastics, with smaller items considered as being opportunistically sampled (Hidalgo-Ruz et al. 2012, Wootton et al. 2024). After each tow, captured solids were rinsed into a 750 mL cod end which was sealed with a lid and stored in a nally bin for transport to Lizard Island Research Station (LIRS).

Mid-column water sampling was conducted using a protocol adapted from previous environmental studies (Liu et al. 2020). A custom-built submersible pump sampler consisting of a battery driven impeller pump (MEI standard 2.2 kW motor, 3phase @ 400 V) connected to a polyvinyl hose (PVC, 18 mm internal diameter x 7 m length) was used. Diving weights (4 kg total) were attached to ensure the pump and hose remained vertical at each sampling depth. Prior to each sampling event, the pump was operated in situ for 2 min at a flowrate of 45 L min^-1^ to flush the device and avoid cross contamination between samples (i.e., water was not sampled at this time). Mid-column sampling was conducted at depth, ranging from 4 to 6 m dependent on local seafloor bathymetry (Table S1). Sampling involved pumping mid-column water at a rate of 45 L min^-1^ over stacked 263 and 37 µm mesh sieves. After each sampling event, the pair of stacked mesh sieves was fully covered with aluminium foil and stored upright in nally bins for transport to LIRS. As the deck of the boat is exposed to potential airborne contamination during sampling, Milli-Q water blanks were placed adjacent to the sampling sieves (see “microplastic contamination control” for more information) while sampling was underway.

Seafloor sediment samples

Sediment samples were collected by SCUBA. Quadrats (white PVC pipes; 0.5 m^2^) were positioned on relatively flat areas of the seafloor (i.e., absence of sand banks or ripples to ensure homogenous volume of sample collected per quadrat area) in the vicinity of sea cucumber sightings to further facilitate the ecological relevance of the abiotic-biotic (dis-)similarities comparisons and the foraging behaviour of sea cucumbers. Using a stainless-steel trowel, the top 3 cm of surficial sediment was collected representing the surface layer of the seafloor where microplastics deposit (Bakir et al. 2023, Zhang et al. 2022) and sea cucumbers foraging occurs (Graham &Thompson 2009, Uthicke 1999). Samples were carefully excavated and transferred into a 15 L polyethylene (PE) plastic bag positioned flat against the quadrat edge. The bag opening was unfurled immediately before the transfer of sediment; after transfer the bag was lifted upright, and sediment allowed to settle to the bottom over ~1 min. The bag was gently squeezed to push out excess water and the opening sealed with a knot and rubber band. Sediment samples were taken to the surface and transferred to nally bins on the boat for transport to LIRS.

Organisms

All sampled organisms were collected from various locations along the fringing reefs of Granite Bluff and Blue Lagoon using SCUBA. Fish were exposed to diluted natural clove oil (1:3:3 parts clove oil:ethanol:seawater), corralled into a fencing net, then captured by green nylon hand net (Kroon 2015). Individual fish were subsequently placed into clear PE ziplock bags, and immediately and humanely euthanized by adding a lethal overdose of clove oil (100% clove oil and > 80% ethanol (EtOH) in a final ratio of 1:3.5) to the bag (Kroon 2015). Fragments of coral and sponge colonies, and whole sea squirt individuals were collected using hammer and chisel. Sea cucumbers were collected by hand. Each invertebrate sample was immediately placed in its own ziplock bag, sealed, and transferred to the boat for euthanasia on ice. Storage in individual bags prevented microplastic cross-contamination between samples and preserved any faeces or regurgitated material for subsequent microplastic analysis if and when these events occurred. All sampled organisms were stored on ice in a cooler box and transported to LIRS where they were immediately pre-processed and preserved.

Table S1: Latitude and longitude (Degrees Decimal Minutes) of surface water, mid-column water and sediment sampling. For surface water tows, coordinates include the start and end points of the 10 min tow. For mid-column water pumping, as sampling was conducted with the boat anchored only one GPS coordinate was recorded.

| Sample type | Sampling site | Replicate | Initial (1) and final (2) position (surface water only) | Latitude | Longitude | Depth (m) |
| --- | --- | --- | --- | --- | --- | --- |
| Surface | Granite Bluff | 1 | 1 | 14˚39.222’S | 145˚26.907’E | 17.8 |
|  |  |  | 2 | 14˚38.773'S | 145˚26.980'E | 21.2 |
|  |  | 2 | 1 | 14˚38.801'S | 145˚27.031'E | 7.7 |
|  |  |  | 2 | 14˚39.265'S | 145˚27.015'E | 8.8 |
|  |  | 3 | 1 | 14˚38.854'S | 145˚27.006'E | 11.7 |
|  |  |  | 2 | 14˚39.327'S | 145˚27.040'E | 3.4 |
|  |  | 4 | 1 | 14˚39.352'S | 145˚27.024'E | 4.2 |
|  |  |  | 2 | 14˚38.916'S | 145˚27.004'E | 19.2 |
|  |  | 5 | 1 | 14˚38.792'S | 145˚27.026'E | 14.2 |
|  |  |  | 2 | 14˚39.328'S | 145˚27.036'E | 3.8 |
|  | Blue Lagoon | 1 | 1 | 14˚41.739'S | 145˚27.128'E | 9.3 |
|  |  |  | 2 | 14˚41.420'S | 145˚27.312'E | 6.8 |
|  |  | 2 | 1 | 14˚41.401'S | 145˚27.430'E | 9.8 |
|  |  |  | 2 | 14˚41.679'S | 145˚27.241'E | 8.6 |
|  |  | 3 | 1 | 14˚41.744'S | 145˚27.130'E | 9.5 |
|  |  |  | 2 | 14˚41.480'S | 145˚27.278'E | 7.6 |
|  |  | 4 | 1 | 14˚41.330'S | 145˚27.357'E | 5.7 |
|  |  |  | 2 | 14˚41.680'S | 145˚27.224'E | 8.6 |
|  |  | 5 | 1 | 14˚41.730'S | 145˚27.150'E | 9.5 |
|  |  |  | 2 | 14˚41.374'S | 145˚27.373'E | 6.9 |
| Mid-column | Granite Bluff | 1 | n/a | 14˚39.290’S | 145˚27.029’E | 8.5 |
|  |  | 2 | n/a | 14˚38.907’S | 145˚27.003’E | 6.8 |
|  |  | 3 | n/a | 14˚39.370’S | 145˚27.017’E | 6.1 |
|  |  | 4 | n/a | 14˚38.984’S | 145˚26.994’E | 8.8 |
|  |  | 5 | n/a | 14˚39.136’S | 145˚27.028’E | 6.3 |
|  | Blue Lagoon | 1 | n/a | 14˚41.729’S | 145˚27.139’E | 10.8 |
|  |  | 2 | n/a | 14˚41.358’S | 145˚27.366’E | 6.6 |
|  |  | 3 | n/a | 14˚41.519’S | 145˚27.285’E | 8.2 |
|  |  | 4 | n/a | 14˚41.530’S | 145˚27.263’E | 8.4 |
|  |  | 5 | n/a | 14˚41.473’S | 145˚27.285’E | 7.8 |

Sample Processing

Table S2: Biometric information (dry weight, g) of collected organisms (replicates of fish, sea squirt, sponge, coral and sea cucumber)

| Sample type | Sampling site | Replicate | Weight (g) |
| --- | --- | --- | --- |
| Fish | Granite Bluff | 1 | 6.00 |
|  |  | 2 | 3.40 |
|  |  | 3 | 5.60 |
|  |  | 4 | 1.90 |
|  |  | 5 | 0.90 |
|  | Blue Lagoon | 1 | 7.40 |
|  |  | 2 | 6.80 |
|  |  | 3 | 8.00 |
|  |  | 4 | 3.60 |
|  |  | 5 | 2.20 |
| Sea squirt | Granite Bluff | 1 | 1.50 |
|  |  | 2 | 3.40 |
|  |  | 3 | 1.80 |
|  |  | 4 | 6.20 |
|  |  | 5 | 7.10 |
|  | Blue Lagoon | 1 | 4.30 |
|  |  | 2 | 6.60 |
|  |  | 3 | 7.30 |
|  |  | 4 | 2.70 |
|  |  | 5 | 3.50 |
| Sponge | Granite Bluff | 1 | 10.11 |
|  |  | 2 | 10.81 |
|  |  | 3 | 10.05 |
|  |  | 4 | 10.57 |
|  |  | 5 | 10.05 |
|  | Blue Lagoon | 1 | 10.67 |
|  |  | 2 | 9.88 |
|  |  | 3 | 10.04 |
|  |  | 4 | 9.99 |
|  |  | 5 | 9.98 |
| Coral | Granite Bluff | 1 | 18.49 |
|  |  | 2 | 19.86 |
|  |  | 3 | 15.70 |
|  |  | 4 | 20.90 |
|  |  | 5 | 19.38 |
|  | Blue Lagoon | 1 | 20.42 |
|  |  | 2 | 22.36 |
|  |  | 3 | 19.66 |
|  |  | 4 | 19.19 |
|  |  | 5 | 19.58 |
| Sea cucumber | Granite Bluff | 1 | 221.80 |
|  |  | 2 | 275.10 |
|  |  | 3 | 329.20 |
|  |  | 4 | 182.70 |
|  |  | 5 | 477.30 |
|  | Blue Lagoon | 1 | 141.30 |
|  |  | 2 | 306.70 |
|  |  | 3 | 127.90 |
|  |  | 4 | 152.50 |
|  |  | 5 | 254.80 |

Microplastic identification and characterization

FTIR spectra of items >350 µm were obtained using a PerkinElmer Spectrum 100 FTIR [1 mm ATR window; pressure gauge = 150; 16 scans at 4 cm-1 resolution; wavenumber range 4000 - 600 cm-1; automated atmospheric (CO2/H2O) suppression and vapor compensation; and background scans acquired after every tenth spectrum acquired]. Items < 350 µm were analysed on a PerkinElmer Spotlight 200i FTIR microscope configured with the Spectrum 100 system [100 μm ATR aperture; pressure gauge = 5%; 32 scans at 4 cm-1 resolution; wavenumber range 4000 - 600 cm-1; automated atmospheric (CO2/H2O) suppression and vapor compensation; and background scans acquired before every spectrum, affording a 10 µm size limit of detection]. Spectra were processed using the PerkinElmer Data Tune-up function. Polymer assignment was categorized as per Table S3.

Table S3: Final assignment (i.e., polymer type) of microplastics isolated from all samples across the eight abiotic (sea surface and mid-column waters and seafloor sediment) and biotic (fish, sea squirt, sponge, coral, and sea cucumber) matrices. Final assignment was based on infrared spectral fingerprinting and cross-referencing with the Contaminant Library, following Kroon et al. (2018a). Items were determined to be synthetic (i.e., plastic polymer) or semi-synthetic (combination of natural fibres and plastic polymers). “other” encompasses polymers found in low quantities in all matrices.

| Final assignment | Primary assignment | Synthetic or semi-synthetic nature |
| --- | --- | --- |
| acrylic | polyacrylate | synthetic |
|  | methyl_methacrylate |  |
|  | acrylic:alkyd_polymer |  |
| ethylene propylene diene (EPDM) | ethylene propylene diene | synthetic |
| nylon | nylon | synthetic |
|  | NFC:nylon | semi- synthetic |
|  | NFC:rayon:nylon |  |
|  | NFC:rayon:nylon:polyurethane |  |
|  | rayon:nylon |  |
|  | keratin:nylon |  |
| polyester/polyethyleneterephthalate (PET) | polyethyleneterephthalate | synthetic |
|  | polyester:nylon |  |
|  | polyester:polyalkyd |  |
|  | polyester:polypropylene:acrylic |  |
|  | rayon:nylon:polyester |  |
|  | NFC:polyester | semi- synthetic |
|  | NFC:rayon:polyester |  |
|  | rayon:polyester |  |
|  | keratin:polyester:nylon |  |
| polyethylene (PE) | polyethylene | synthetic |
| polypropylene (PP) | polypropylene | synthetic |
|  | NFC:polypropylene | semi- synthetic |
| PP:PE | polypropylene:polyethylene | synthetic |
| polystyrene (PS) | polystyrene | synthetic |
| polysulfone (PSU) | polysulfone | synthetic |
| polyvinylchloride (PVC) | polyvinylchloride | synthetic |
| other | epoxy | synthetic |
|  | polyalcohol |  |
|  | polyvinylalcohol |  |
|  | polyurethane:polyacrylate |  |
|  | polyvinylacetate:vinylchloride |  |
|  | melamine |  |
|  | polybutadiene_rubber |  |
|  | polyester:polypropylene:acrylic |  |
|  | polyvinylacetate:polyethylene |  |
|  | alkyd polymer |  |
|  | polyacrylonitrile:butadiene:styrene |  |
|  | soft copolymer |  |
|  | chlorinated polyolefin |  |
|  | epoxypolyester |  |
|  | polyacrylonitrile:butadiene |  |
|  | polyester:polyurethane |  |
|  | polyoxymethylene |  |
|  | polytetrafluoroethylene |  |
|  | polyurethane |  |
|  | polyurethane_rubber |  |
|  | polyvinylbenzyl chloride |  |
|  | rayon:polyurethane | semi- synthetic |
|  | keratin:polyester:nylon |  |
|  | NFC:polyester:polypropylene |  |
|  | NFC:polyurethane |  |

Quality assurance and quality control

Microplastic contamination control

Measures to reduce the likelihood of extraneous microplastic contamination were applied during laboratory-based processing, including wearing cotton clothes and de-linting laboratory coats prior to sample handling. Furthermore, equipment, tools and working area were cleaned prior to use with Milli-Q water and filtered 70% Ethanol (0.45 μm Millipore® HA filters) per Santana et al. (2021). For sample processing, both NaCl and KI solutions were filtered to 0.45 μm; 70% HNO3 was not filtered for safety reasons. Instead, to assess HNO3 as a potential source of extraneous microplastic contamination, 15 mL of the acid was neutralized using 0.45 μm pre-filtered 10% potassium hydroxide (KOH) solution and filtered onto 26 µm stainless steel filters for verification under microscopy and, if necessary, FTIR. No microplastics were found in HNO3 samples neutralized in this manner. Density separations and acid digestions were conducted in a fume hood.

Airborne contamination was monitored using Milli-Q water blanks for those collection and processing steps whereby samples were exposed to air (n = 4 for each day, placed equidistance and adjacent to the sample and opened whenever a sample was exposed to the air). For mid-column seawater sampling the stacked sieves were exposed to air throughout the process, therefore Milli-Q water blanks (n = 4 x 50 mm petri dishes) were collected concurrently (again equidistance and adjacent to the sieves). Milli-Q water blanks (n = 4 x 50 mL beakers) were collected during all laboratory procedures, including sample pre-processing at LIRS, and sample processing at AIMS (i.e., dissections, Bogorov sorting, sample clarification and filtration), again placed equidistance and adjacent to the activity. All Milli-Q water blanks were filtered onto 26 µm stainless steel filters (Schlawinsky et al. 2022) for microplastic identification and characterisation using microscopy and FTIR (Kroon et al. 2018a).

Representative sample of each utensil and airborne microplastics were added into a project-specific plastic contaminant library following Kroon et al. (2018a)) and detailed in Table S4.

Table S4: Contaminant library built for each abiotic and biotic matrix: (a) surface water, (b) mid-column water, (c) seafloor sediment, (d) fish gastrointestinal tract (GIT), (e) sea squirt innards, (f) sponge, (g) coral, and (h) sea cucumber GIT contents. Items from the contaminant library are described according to origin (blank or plastic item used), material, shape and colour.

(a) Contaminant library for surface water samples.

| Contaminant item | shape | colour |
| --- | --- | --- |
| Blank_putative microplastic_1 | transparent | fragment |
| Blank_putative microplastic_2 | transparent | fragment |
| Blank_putative microplastic_3 | transparent | fragment |
| Blank_putative microplastic_4 | white | fibre |
| Blank_putative microplastic_5 | white | fibre |
| Blank_putative microplastic_6 | white | fibre |
| Blank_putative microplastic_7 | black | fragment |
| Blank_putative microplastic_8 | white | fragment |
| Blank_putative microplastic_9 | transparent | fibre |
| Blank_putative microplastic_10 | blue | fibre |
| Blank_putative microplastic_11 | brown | fragment |
| Blank_putative microplastic_12 | white | fibre |
| Blank_putative microplastic_13 | brown | fibre |
| Blank_putative microplastic_14 | black | fibre |
| Blank_putative microplastic_15 | black | fibre |
| Blank_putative microplastic_16 | transparent | fibre |
| Blank_putative microplastic_17 | transparent | fibre |
| Blank_putative microplastic_18 | brown | fibre |
| Blank_putative microplastic_19 | brown | fibre |
| Blank_putative microplastic_20 | brown | fragment |
| Blank_putative microplastic_21 | brown | fibre |
| Blank_putative microplastic_22 | transparent | fibre |
| Blank_putative microplastic_23 | brown | fibre |
| Blank_putative microplastic_24 | transparent | fibre |
| Field_Charcoal | black | fragment |
| Field_Coal | black | fragment |
| Field_Clear_Flask_PP_sample container | transparent | fragment |
| Field_EtOH_Bottle Lid | blue | fragment |
| Field_Kapok Fibre | transparent | fibre |
| Field_Yellow_Lid_PE_sample container | yellow | fragment |
| Field_Yellow Paint | yellow | fragment |
| Field_40um Plankton Filter | transparent | fibre |
| Field_350um Plankton Filter | transparent | fibre |
| Field_Black Paint | black | fragment |
| Field_Carpet_Blue | transparent | fibre |
| Field_Carpet_Grey | transparent | fibre |
| Field_Filament of yellow and grey rope_grey filament | transparent | fibre |
| Field_Filament of yellow and grey rope_yellow filament | transparent | fibre |
| Field_Filament of clear rope | transparent | fibre |
| General_Green AIMS TShirt | green | fibre |
| General_Green Chile TShirt | green | fibre |
| General_Parafilm | transparent | fragment |
| General_Spray Bottle Lid_Red | red | fragment |
| General_White TShirt | transparent | fibre |
| General_Wine Shirt | red | fibre |
| Lab_BlueSilicone_O-ring | blue | fragment |
| Lab_Cotton Lab Coat | green | fibre |
| Lab_Gloves | blue | fragment |
| Lab_Grill Filter | transparent | fibre |
| Lab_Red_Stopper | red | fragment |
| Lab_Spray Bottle Lid_Teflon | white | fragment |
| Lab_White Stopper | white | fragment |

(b) Contaminant library for mid-column water samples.

| Contaminant item | shape | colour |
| --- | --- | --- |
| Blank_putative microplastic_1 | transparent | fibre |
| Blank_putative microplastic_2 | white | fragment |
| Blank_putative microplastic_3 | white | fragment |
| Blank_putative microplastic_4 | green | fibre |
| Blank_putative microplastic_5 | transparent | fibre |
| Blank_putative microplastic_6 | transparent | fibre |
| Blank_putative microplastic_7 | transparent | fibre |
| Blank_putative microplastic_8 | transparent | fibre |
| Blank_putative microplastic_9 | transparent | fibre |
| Blank_putative microplastic_10 | brown | fibre |
| Blank_putative microplastic_11 | red | fibre |
| Blank_putative microplastic_12 | transparent | fibre |
| Blank_putative microplastic_13 | black | fibre |
| Blank_putative microplastic_14 | yellow | fragment |
| Blank_putative microplastic_15 | brown | fragment |
| Blank_putative microplastic_16 | white | fragment |
| Blank_putative microplastic_17 | blue | fibre |
| Blank_putative microplastic_18 | transparent | fibre |
| Blank_putative microplastic_19 | transparent | fibre |
| Blank_putative microplastic_20 | transparent | fibre |
| Blank_putative microplastic_21 | transparent | fibre |
| Blank_putative microplastic_22 | brown | fibre |
| Blank_putative microplastic_23 | brown | fragment |
| Blank_putative microplastic_24 | brown | fibre |
| Blank_putative microplastic_25 | transparent | fibre |
| Blank_putative microplastic_26 | transparent | fibre |
| Blank_putative microplastic_27 | brown | fibre |
| Blank_putative microplastic_28 | transparent | fibre |
| Blank_putative microplastic_29 | transparent | fibre |
| Blank_putative microplastic_30 | white | fragment |
| Blank_putative microplastic_31 | white | fragment |
| Blank_putative microplastic_32 | white | fragment |
| Blank_putative microplastic_33 | white | fragment |
| Blank_putative microplastic_34 | white | fragment |
| Blank_putative microplastic_35 | white | fragment |
| Blank_putative microplastic_36 | white | fragment |
| Blank_putative microplastic_37 | white | fragment |
| Blank_putative microplastic_38 | white | fragment |
| Blank_putative microplastic_39 | white | fragment |
| Blank_putative microplastic_40 | white | fragment |
| Blank_putative microplastic_41 | white | fragment |
| Blank_putative microplastic_42 | white | fragment |
| Blank_putative microplastic_43 | white | fragment |
| Blank_putative microplastic_44 | white | fragment |
| Blank_putative microplastic_45 | transparent | fibre |
| Field_Charcoal | black | fragment |
| Field_Coal | black | fragment |
| Field_Clear_Flask_PP_sample container | transparent | fragment |
| Field_EtOH_Bottle Lid | blue | fragment |
| Field_Kapok Fibre | transparent | fibre |
| Field_Yellow_Lid_PE_sample container | yellow | fragment |
| Field_Yellow Paint | yellow | fragment |
| Field_40um Plankton Filter | transparent | fibre |
| Field_350um Plankton Filter | transparent | fibre |
| Field_Black Paint | black | fragment |
| Field_Carpet_Blue | blue | fibre |
| Field_Carpet_Grey | white | fibre |
| Field_Filament of yellow and grey rope_grey filament | white | fibre |
| Field_Filament of yellow and grey rope_yellow filament | yellow | fibre |
| Field_Filament of clear rope | transparent | fibre |
| Field_MPP plankton net canvas 17 03 2016 | white | fibre |
| Field_MPP plankton net codend bottle 17 03 2016 | transparent | fragment |
| Field_MPP plankton net nylon 17 03 2016 | transparent | fibre |
| General_Green AIMS TShirt | green | fibre |
| General_Green Chile TShirt | green | fibre |
| General_Parafilm | transparent | fragment |
| General_Spray Bottle Lid_Red | red | fragment |
| General_White TShirt | transparent | fibre |
| General_Wine Shirt | red | fibre |
| Lab_BlueSilicone_O-ring | blue | fragment |
| Lab_Cotton Lab Coat | green | fibre |
| Lab_Gloves | blue | fragment |
| Lab_Grill Filter | transparent | fibre |
| Lab_Red_Stopper | red | fragment |
| Lab_Spray Bottle Lid_Teflon | white | fragment |
| Lab_White Stopper | white | fragment |

(c) Contaminant library for seafloor sediment samples.

| Contaminant item | shape | colour |
| --- | --- | --- |
| Blank_putative microplastic_1 | white | fragment |
| Blank_putative microplastic_2 | white | fragment |
| Blank_putative microplastic_3 | white | fragment |
| Blank_putative microplastic_4 | white | fragment |
| Blank_putative microplastic_5 | white | fragment |
| Blank_putative microplastic_6 | white | fragment |
| Blank_putative microplastic_7 | white | fragment |
| Blank_putative microplastic_8 | white | fragment |
| Blank_putative microplastic_9 | white | fragment |
| Blank_putative microplastic_10 | white | fragment |
| Blank_putative microplastic_11 | white | fragment |
| Blank_putative microplastic_12 | white | fragment |
| Blank_putative microplastic_13 | transparent | fragment |
| Blank_putative microplastic_14 | white | fibre |
| Blank_putative microplastic_15 | transparent | fragment |
| Blank_putative microplastic_16 | transparent | fibre |
| Blank_putative microplastic_17 | transparent | fibre |
| Field_Charcoal | black | fragment |
| Field_Coal | black | fragment |
| Field_PVC Quadrat | white | fragment |
| Field_EtOH_Bottle Lid | blue | fragment |
| Field_Kapok Fibre | transparent | fibre |
| Field_Sediment Plastic Bag | transparent | fragment |
| General_Green AIMS TShirt | green | fibre |
| General_Green Chile TShirt | green | fibre |
| General_Parafilm | transparent | fragment |
| General_Spray Bottle Lid_Red | red | fragment |
| General_White TShirt | transparent | fibre |
| General_Wine Shirt | red | fibre |
| Lab_BlueSilicone_O-ring | blue | fragment |
| Lab_Cotton Lab Coat | green | fibre |
| Lab_Gloves | blue | fragment |
| Lab_Grill Filter | transparent | fibre |
| Lab_Red_Stopper | red | fragment |
| Lab_Spray Bottle Lid_Teflon | white | fragment |
| Lab_White Stopper | white | fragment |

(d) Contaminant library for fish samples.

| Contaminant item | shape | colour |
| --- | --- | --- |
| Blank_putative microplastic_1 | red | fragment |
| Blank_putative microplastic_2 | pink | fragment |
| Blank_putative microplastic_3 | transparent | fibre |
| Blank_putative microplastic_4 | black | fragment |
| Blank_putative microplastic_5 | brown | fibre |
| Blank_putative microplastic_6 | transparent | fibre |
| Blank_putative microplastic_7 | transparent | fibre |
| Blank_putative microplastic_8 | transparent | fibre |
| Blank_putative microplastic_9 | transparent | fragment |
| Blank_putative microplastic_10 | blue | fragment |
| Blank_putative microplastic_11 | transparent | fragment |
| Blank_putative microplastic_12 | transparent | fragment |
| Blank_putative microplastic_13 | transparent | fragment |
| Blank_putative microplastic_14 | orange | fibre |
| Blank_putative microplastic_15 | transparent | fragment |
| Blank_putative microplastic_16 | black | fibre |
| Field_Charcoal | black | fragment |
| Field_Coal | black | fragment |
| Field_Clear_Flask_PP_sample container | transparent | fragment |
| Field_EtOH_Bottle Lid | blue | fragment |
| Field_Kapok Fibre | transparent | fibre |
| Field_Yellow_Lid_PE_sample container | yellow | fragment |
| Field_Zip Lock bag | transparent | fragment |
| Field_Fishing Net | green | fibre |
| General_Green AIMS TShirt | green | fibre |
| General_Green Chile TShirt | green | fibre |
| General_Parafilm | transparent | fragment |
| General_Spray Bottle Lid_Red | red | fragment |
| General_White TShirt | transparent | fibre |
| General_Wine Shirt | red | fibre |
| Lab_BlueSilicone_O-ring | blue | fragment |
| Lab_Cotton Lab Coat | green | fibre |
| Lab_Gloves | blue | fragment |
| Lab_Grill Filter | transparent | fibre |
| Lab_Red_Stopper | red | fragment |
| Lab_Spray Bottle Lid_Teflon | white | fragment |
| Lab_White Stopper | white | fragment |
| Lab_Glass pipette head | red | fragment |

(e) Contaminant library for sea squirt samples.

| Contaminant item | shape | colour |
| --- | --- | --- |
| Blank_putative microplastic_1 | transparent | fragment |
| Blank_putative microplastic_2 | transparent | fragment |
| Blank_putative microplastic_3 | transparent | fragment |
| Blank_putative microplastic_4 | white | fibre |
| Blank_putative microplastic_5 | white | fibre |
| Blank_putative microplastic_6 | white | fibre |
| Blank_putative microplastic_7 | black | fragment |
| Blank_putative microplastic_8 | white | fragment |
| Blank_putative microplastic_9 | transparent | fibre |
| Blank_putative microplastic_10 | blue | fibre |
| Blank_putative microplastic_11 | brown | fragment |
| Blank_putative microplastic_12 | white | fibre |
| Blank_putative microplastic_13 | brown | fibre |
| Blank_putative microplastic_14 | black | fibre |
| Blank_putative microplastic_15 | brown | fibre |
| Field_Charcoal | black | fragment |
| Field_Coal | black | fragment |
| Field_Clear_Flask_PP_sample container | transparent | fragment |
| Field_EtOH_Bottle Lid | blue | fragment |
| Field_Kapok Fibre | transparent | fibre |
| Field_Yellow_Lid_PE_sample container | yellow | fragment |
| Field_Zip Lock bag | transparent | fragment |
| General_Green AIMS TShirt | green | fibre |
| General_Green Chile TShirt | green | fibre |
| General_Parafilm | transparent | fragment |
| General_Spray Bottle Lid_Red | red | fragment |
| General_White TShirt | transparent | fibre |
| General_Wine Shirt | red | fibre |
| Lab_BlueSilicone_O-ring | blue | fragment |
| Lab_Cotton Lab Coat | green | fibre |
| Lab_Gloves | blue | fragment |
| Lab_Grill Filter | transparent | fibre |
| Lab_Red_Stopper | red | fragment |
| Lab_Spray Bottle Lid_Teflon | white | fragment |
| Lab_White Stopper | white | fragment |

(f) Contaminant library for sponge samples.

| Contaminant item | shape | colour |
| --- | --- | --- |
| Blank_putative microplastic_1 | transparent | fragment |
| Blank_putative microplastic_2 | transparent | fragment |
| Blank_putative microplastic_3 | white | fragment |
| Blank_putative microplastic_4 | transparent | fragment |
| Blank_putative microplastic_5 | transparent | fibre |
| Blank_putative microplastic_6 | white | fragment |
| Blank_putative microplastic_7 | transparent | fibre |
| Blank_putative microplastic_8 | transparent | fragment |
| Blank_putative microplastic_9 | transparent | fibre |
| Blank_putative microplastic_10 | transparent | fragment |
| Blank_putative microplastic_11 | white | fragment |
| Blank_putative microplastic_12 | transparent | fibre |
| Blank_putative microplastic_13 | black | fragment |
| Blank_putative microplastic_14 | brown | fibre |
| Blank_putative microplastic_15 | transparent | fibre |
| Blank_putative microplastic_16 | transparent | fibre |
| Blank_putative microplastic_17 | transparent | fibre |
| Blank_putative microplastic_18 | transparent | fibre |
| Blank_putative microplastic_19 | transparent | fragment |
| Blank_putative microplastic_20 | transparent | fibre |
| Blank_putative microplastic_21 | transparent | fragment |
| Blank_putative microplastic_22 | black | fibre |
| Blank_putative microplastic_23 | red | fibre |
| Blank_putative microplastic_24 | white | fragment |
| Blank_putative microplastic_25 | white | fragment |
| Blank_putative microplastic_26 | white | fragment |
| Blank_putative microplastic_27 | white | fragment |
| Blank_putative microplastic_28 | white | fragment |
| Blank_putative microplastic_29 | white | fragment |
| Blank_putative microplastic_30 | white | fragment |
| Blank_putative microplastic_31 | white | fragment |
| Field_Charcoal | black | fragment |
| Field_Coal | black | fragment |
| Field_Clear_Flask_PP_sample container | transparent | fragment |
| Field_EtOH_Bottle Lid | blue | fragment |
| Field_Kapok Fibre | transparent | fibre |
| Field_Yellow_Lid_PE_sample container | yellow | fragment |
| Field_Zip Lock bag | transparent | fragment |
| General_Green AIMS TShirt | green | fibre |
| General_Green Chile TShirt | green | fibre |
| General_Parafilm | transparent | fragment |
| General_Spray Bottle Lid_Red | red | fragment |
| General_White TShirt | transparent | fibre |
| General_Wine Shirt | red | fibre |
| Lab_BlueSilicone_O-ring | blue | fragment |
| Lab_Cotton Lab Coat | green | fibre |
| Lab_Gloves | blue | fragment |
| Lab_Grill Filter | transparent | fibre |
| Lab_Red_Stopper | red | fragment |
| Lab_Spray Bottle Lid_Teflon | white | fragment |
| Lab_White Stopper | white | fragment |

(g) Contaminant library for coral samples.

| Contaminant item | shape | colour |
| --- | --- | --- |
| Blank_putative microplastic_1 | transparent | fragment |
| Blank_putative microplastic_2 | transparent | fragment |
| Blank_putative microplastic_3 | white | fragment |
| Blank_putative microplastic_4 | transparent | fragment |
| Blank_putative microplastic_5 | transparent | fibre |
| Blank_putative microplastic_6 | white | fragment |
| Blank_putative microplastic_7 | transparent | fibre |
| Blank_putative microplastic_8 | transparent | fragment |
| Blank_putative microplastic_9 | transparent | fibre |
| Blank_putative microplastic_10 | transparent | fragment |
| Blank_putative microplastic_11 | white | fragment |
| Blank_putative microplastic_12 | transparent | fibre |
| Blank_putative microplastic_13 | black | fragment |
| Blank_putative microplastic_14 | brown | fibre |
| Blank_putative microplastic_15 | transparent | fibre |
| Blank_putative microplastic_16 | transparent | fibre |
| Blank_putative microplastic_17 | transparent | fibre |
| Blank_putative microplastic_18 | white | fragment |
| Blank_putative microplastic_19 | transparent | fibre |
| Blank_putative microplastic_20 | transparent | fibre |
| Blank_putative microplastic_21 | transparent | fibre |
| Blank_putative microplastic_22 | transparent | fibre |
| Blank_putative microplastic_23 | transparent | fibre |
| Blank_putative microplastic_24 | transparent | fibre |
| Blank_putative microplastic_25 | transparent | fibre |
| Field_Charcoal | black | fragment |
| Field_Coal | black | fragment |
| Field_Clear_Flask_PP_sample container | transparent | fragment |
| Field_EtOH_Bottle Lid | blue | fragment |
| Field_Kapok Fibre | transparent | fibre |
| Field_Yellow_Lid_PE_sample container | yellow | fragment |
| Field_Zip Lock bag | transparent | fragment |
| General_Green AIMS TShirt | green | fibre |
| General_Green Chile TShirt | green | fibre |
| General_Parafilm | transparent | fragment |
| General_Spray Bottle Lid_Red | red | fragment |
| General_White TShirt | transparent | fibre |
| General_Wine Shirt | red | fibre |
| Lab_BlueSilicone_O-ring | blue | fragment |
| Lab_Cotton Lab Coat | green | fibre |
| Lab_Gloves | blue | fragment |
| Lab_Grill Filter | transparent | fibre |
| Lab_Red_Stopper | red | fragment |
| Lab_Spray Bottle Lid_Teflon | white | fragment |
| Lab_White Stopper | white | fragment |

(h) Contaminant library for sea cucumber samples.

| Contaminant item | shape | colour |
| --- | --- | --- |
| Blank_putative microplastic_1 | transparent | fibre |
| Blank_putative microplastic_2 | transparent | fragment |
| Blank_putative microplastic_3 | brown | fibre |
| Blank_putative microplastic_4 | red | fibre |
| Blank_putative microplastic_5 | transparent | fibre |
| Blank_putative microplastic_6 | black | fibre |
| Blank_putative microplastic_7 | transparent | fragment |
| Blank_putative microplastic_8 | brown | fragment |
| Blank_putative microplastic_9 | white | fragment |
| Blank_putative microplastic_10 | blue | fibre |
| Blank_putative microplastic_11 | transparent | fibre |
| Blank_putative microplastic_12 | transparent | fibre |
| Blank_putative microplastic_13 | transparent | fibre |
| Blank_putative microplastic_14 | transparent | fibre |
| Blank_putative microplastic_15 | red | fragment |
| Blank_putative microplastic_16 | pink | fragment |
| Blank_putative microplastic_17 | transparent | fragment |
| Blank_putative microplastic_18 | transparent | fragment |
| Blank_putative microplastic_19 | white | fragment |
| Blank_putative microplastic_20 | white | fragment |
| Blank_putative microplastic_21 | transparent | fibre |
| Field_Charcoal | black | fragment |
| Field_Coal | black | fragment |
| Field_Clear_Flask_PP_sample container | transparent | fragment |
| Field_EtOH_Bottle Lid | blue | fragment |
| Field_Kapok Fibre | transparent | fibre |
| Field_Yellow_Lid_PE_sample container | yellow | fragment |
| Field_Zip Lock bag | transparent | fragment |
| General_Green AIMS TShirt | green | fibre |
| General_Green Chile TShirt | green | fibre |
| General_Parafilm | transparent | fragment |
| General_Spray Bottle Lid_Red | red | fragment |
| General_White TShirt | transparent | fibre |
| General_Wine Shirt | red | fibre |
| Lab_BlueSilicone_O-ring | blue | fragment |
| Lab_Cotton Lab Coat | green | fibre |
| Lab_Gloves | blue | fragment |
| Lab_Grill Filter | transparent | fibre |
| Lab_Red_Stopper | red | fragment |
| Lab_Spray Bottle Lid_Teflon | white | fragment |
| Lab_White Stopper | white | fragment |

Microplastic tracking through numerical modelling

Spatial Domain


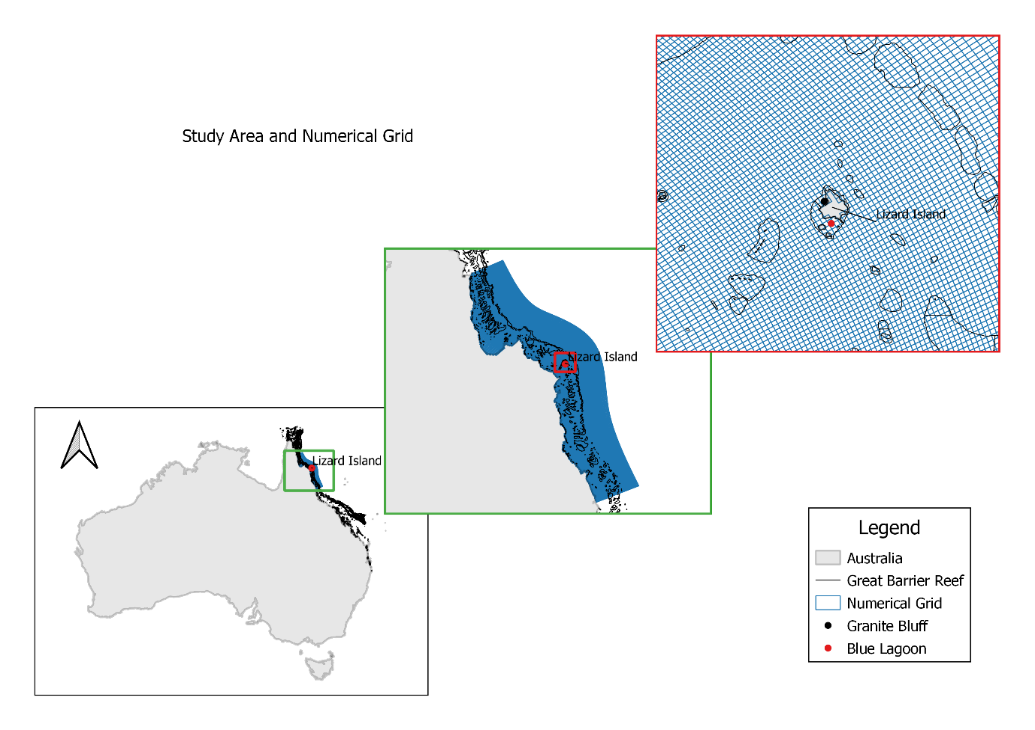


Figure S1: Spatial domain of the numerical simulations used to assess microplastics trajectories to and from Lizard Island. The highest spatial resolution inset (top right) shows the microplastic particles sampling sites, Granite Bluff (black) and Blue Lagoon (red) at Lizard Island, Australia. Numerical grid is shown in blue.

Windage

The influence of wind on particle deflection (windage) is also a function of the particle characteristics (Chubarenko et al. 2016), but essentially, the higher the windage (i.e., above 3%) the greater the potential for the wind to create water turbulence, causing buoyant microplastics to rise to the sea surface, and augment particle drift speed and hence spread (Hrycik et al. 2013).

Table S5: Key parameters used in the numerical simulations to assess microplastics trajectories to and from Granite Bluff (GB) and Blue Lagoon (BL) at Lizard Island, Australia.

| Tracking | Start time (local time) | Seeding (sampling) Site | Windage (%) | # particles |
| --- | --- | --- | --- | --- |
| Hindcast | 04/Oct/2018 11:00h | Granite Bluff | 0 | 1850 |
|  |  |  | 1 | 1850 |
|  |  |  | 2 | 1850 |
|  |  |  | 3 | 1850 |
|  |  |  | 4 | 1850 |
|  | 05/Oct/2018 09:00h | Blue Lagoon | 0 | 1330 |
|  |  |  | 1 | 1330 |
|  |  |  | 2 | 1330 |
|  |  |  | 3 | 1330 |
|  |  |  | 4 | 1330 |
|  | 06/Oct/2018 09:00h | Blue Lagoon | 0 | 520 |
|  |  |  | 1 | 520 |
|  |  |  | 2 | 520 |
|  |  |  | 3 | 520 |
|  |  |  | 4 | 520 |
|  | 06/Oct/2018 16:00h | Granite Bluff | 0 | 220 |
|  |  |  | 1 | 220 |
|  |  |  | 2 | 220 |
|  |  |  | 3 | 220 |
|  |  |  | 4 | 220 |
|  | 07/Oct/2018 09:00h | Granite Bluff | 0 | 1050 |
|  |  |  | 1 | 1050 |
|  |  |  | 2 | 1050 |
|  |  |  | 3 | 1050 |
|  |  |  | 4 | 1050 |
| Forecast | 04/Oct/2018 12:00h | Granite Bluff | 0 | 1850 |
|  |  |  | 1 | 1850 |
|  |  |  | 2 | 1850 |
|  |  |  | 3 | 1850 |
|  |  |  | 4 | 1850 |
|  | 05/Oct/2018 10:00h | Blue Lagoon | 0 | 1330 |
|  |  |  | 1 | 1330 |
|  |  |  | 2 | 1330 |
|  |  |  | 3 | 1330 |
|  |  |  | 4 | 1330 |
|  | 06/Oct/2018 10:00h | Blue Lagoon | 0 | 520 |
|  |  |  | 1 | 520 |
|  |  |  | 2 | 520 |
|  |  |  | 3 | 520 |
|  |  |  | 4 | 520 |
|  | 06/Oct/2018 17:00h | Granite Bluff | 0 | 220 |
|  |  |  | 1 | 220 |
|  |  |  | 2 | 220 |
|  |  |  | 3 | 220 |
|  |  |  | 4 | 220 |
|  | 07/Oct/2018 10:00h | Granite Bluff | 0 | 1050 |
|  |  |  | 1 | 1050 |
|  |  |  | 2 | 1050 |
|  |  |  | 3 | 1050 |
|  |  |  | 4 | 1050 |

Statistical analysis


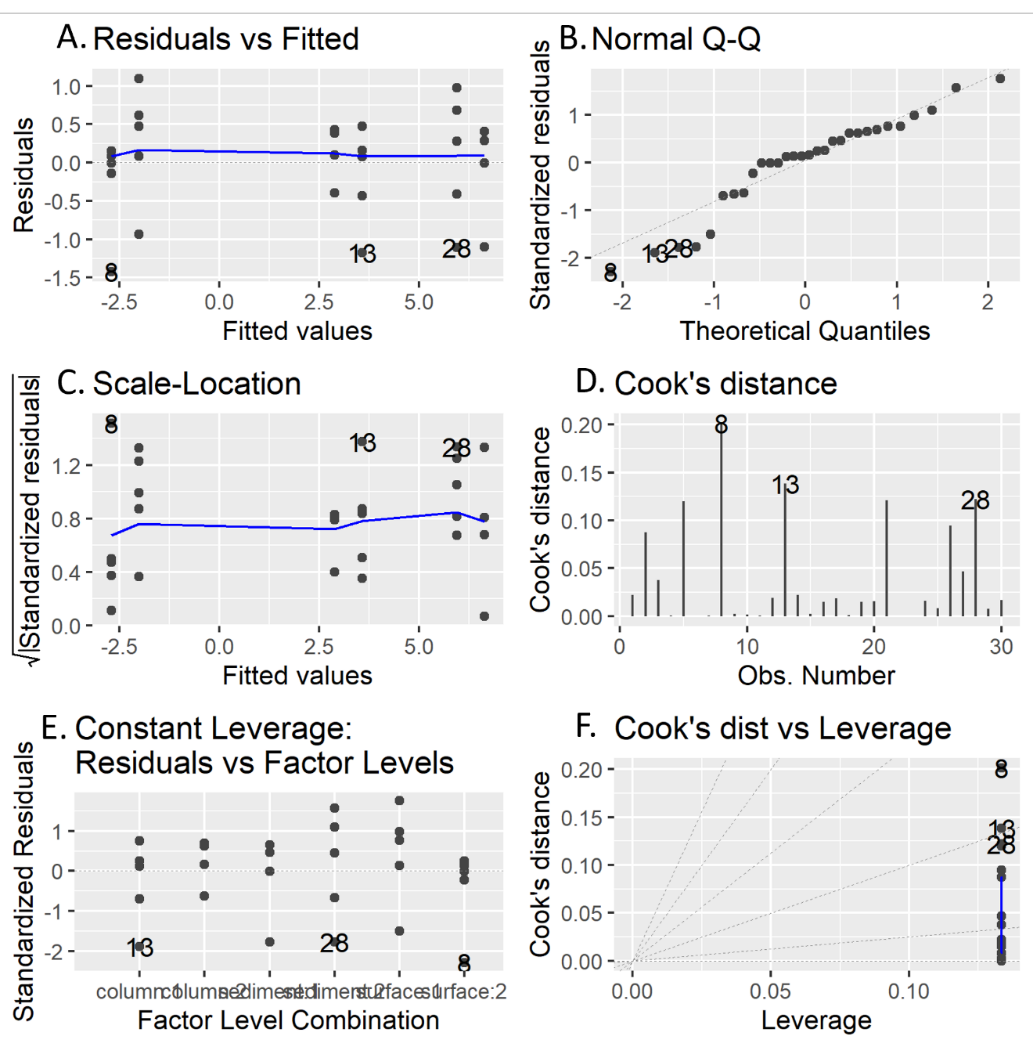


Figure S2: Diagnostic plots for the linear regression model assessing differences in microplastics abundance between abiotic compartments (sea surface and mid-column waters, and seafloor sediment) and between sampling sites (Granitte Bluff and Blue Lagoon): residuals vs. fitted values (A), Q-Q plot of standardized residuals (B), scale-location plot of square root of standardized residuals (C), Cook's distance plot (D), residuals vs. leverage plot (E), and Cook's distance vs. leverage plot (F).


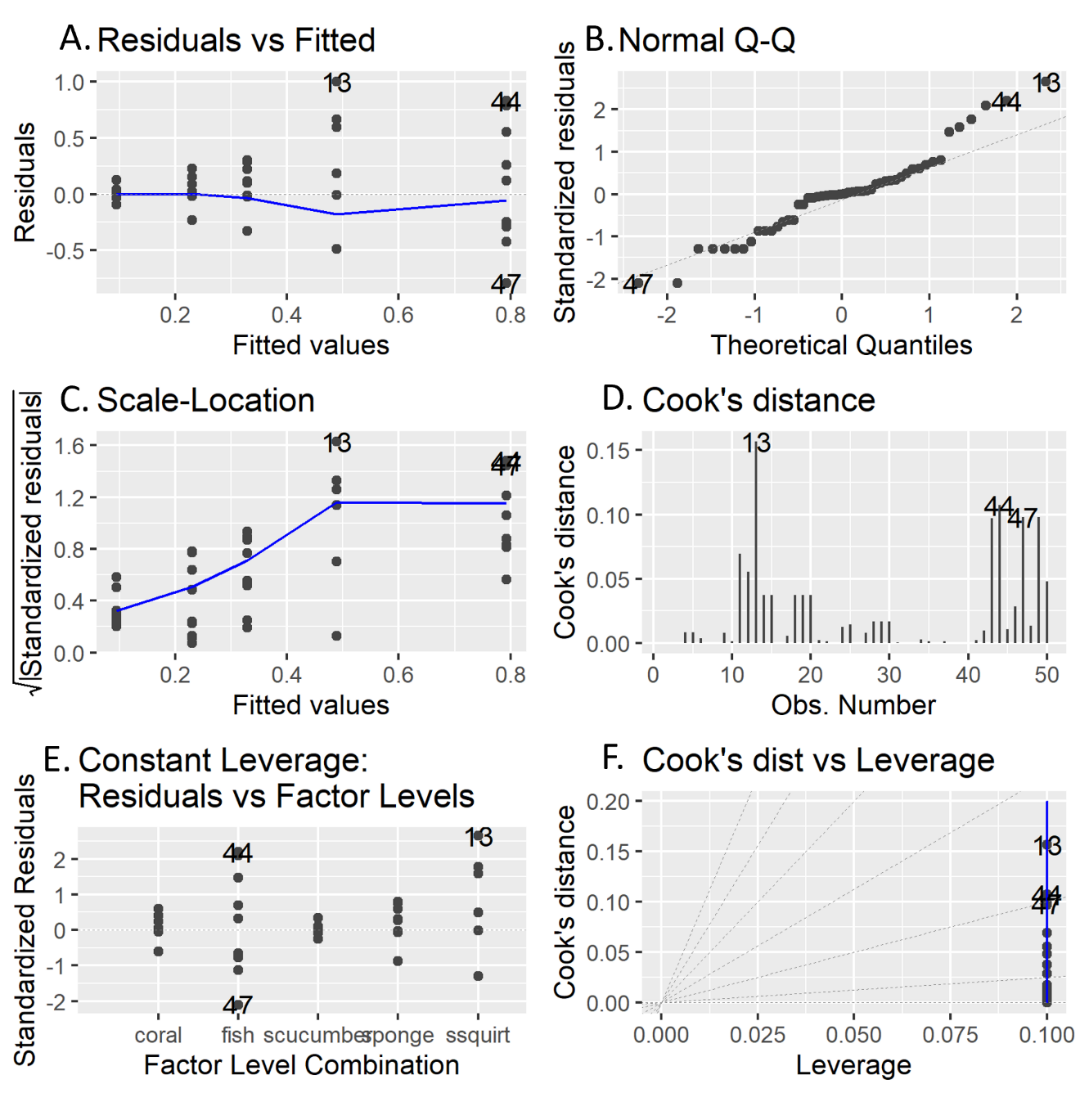


Figure S3: Diagnostic plots for the linear regression model assessing differences in microplastics abundance between biotic matrices (fish, sea squirt, sponge, coral, and sea cucumber): residuals vs. fitted values (A), Q-Q plot of standardized residuals (B), scale-location plot of square root of standardized residuals (C), residuals vs. leverage plot (D), Cook's distance plot (E), and Cook's distance vs. leverage plot (F).


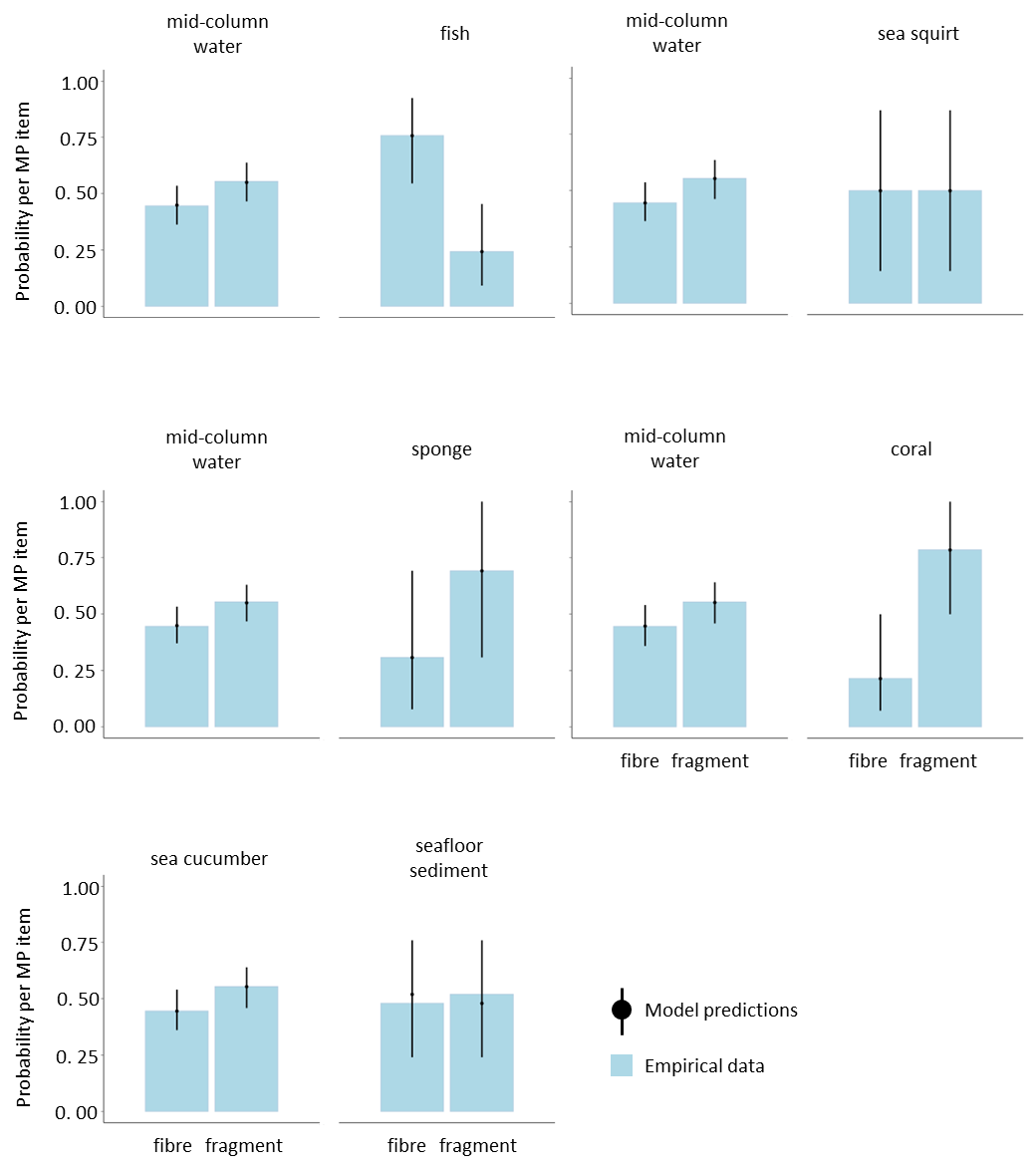


Figure S4: Bayesian regression model validation - Posterior predictive checks of proposed Eq. 3: $microplastic characteristic\sim0+Intercept+matrix$, with shape as microplastic characteristic. Relative frequency of occurrence of each microplastic colour in abiotic (left column) and biotic (right column) matrices. Blue bars represent the observed proportions, black point is the mean predicted proportion and horizontal error bars depicts the Bayesian 95% credible intervals. (A-D) illustrates posterior checks for mid-column water and (A) fish, (B) sea squirt, (C) sponge, (D) coral. (E) illustrates posterior checks for seafloor sediment and sea cucumber.


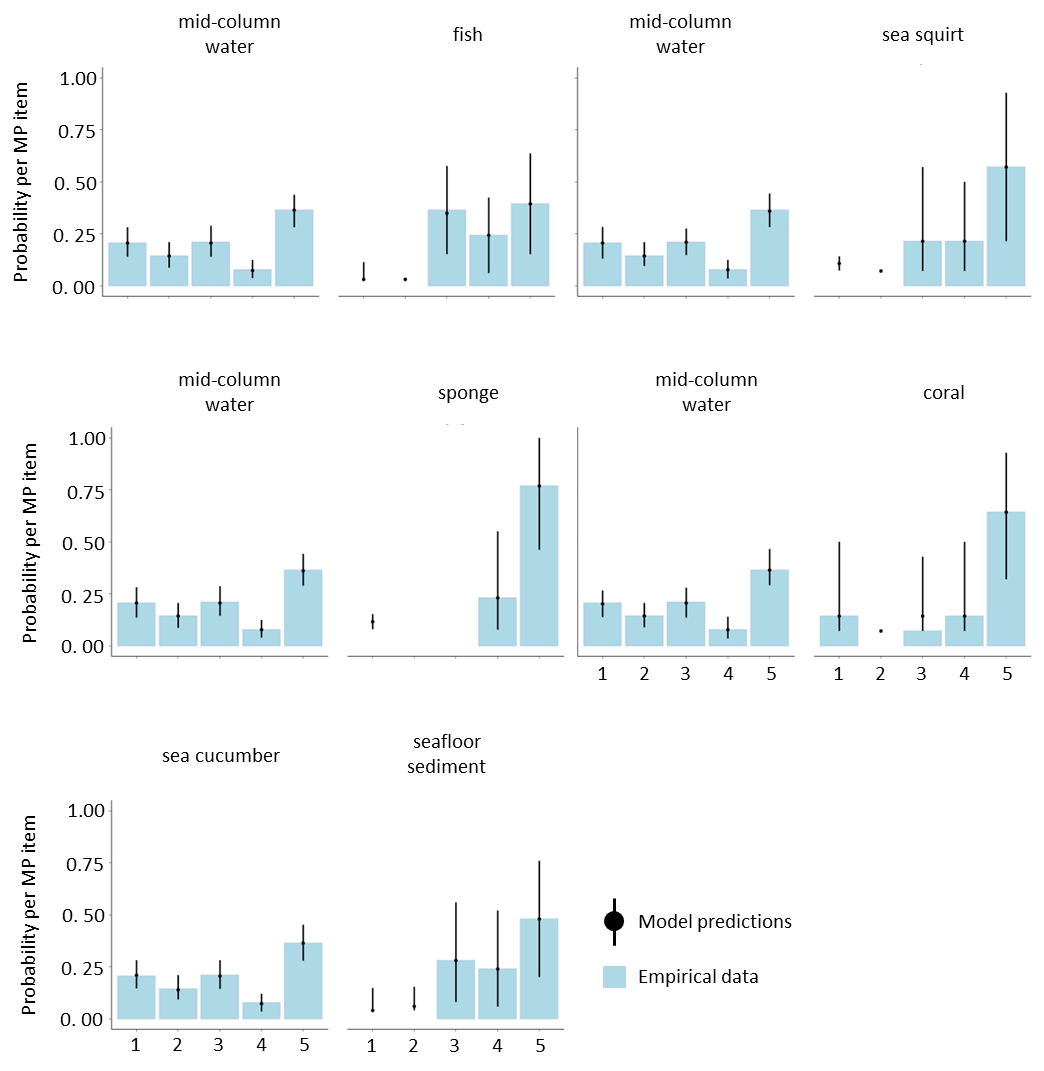


Figure S5: Bayesian regression model validation - Posterior predictive checks of proposed Eq. 3: $microplastic characteristic\sim0+Intercept+matrix$, with size class as microplastic characteristic. Relative frequency of occurrence of each microplastic colour in abiotic (left column) and biotic (right column) matrices. Blue bars represent the observed proportions, black point is the mean predicted proportion and horizontal error bars depicts the Bayesian 95% credible intervals. (A-D) illustrates posterior checks for mid-column water and (A) fish, (B) sea squirt, (C) sponge, (D) coral. (E) illustrates posterior checks for seafloor sediment and sea cucumber.


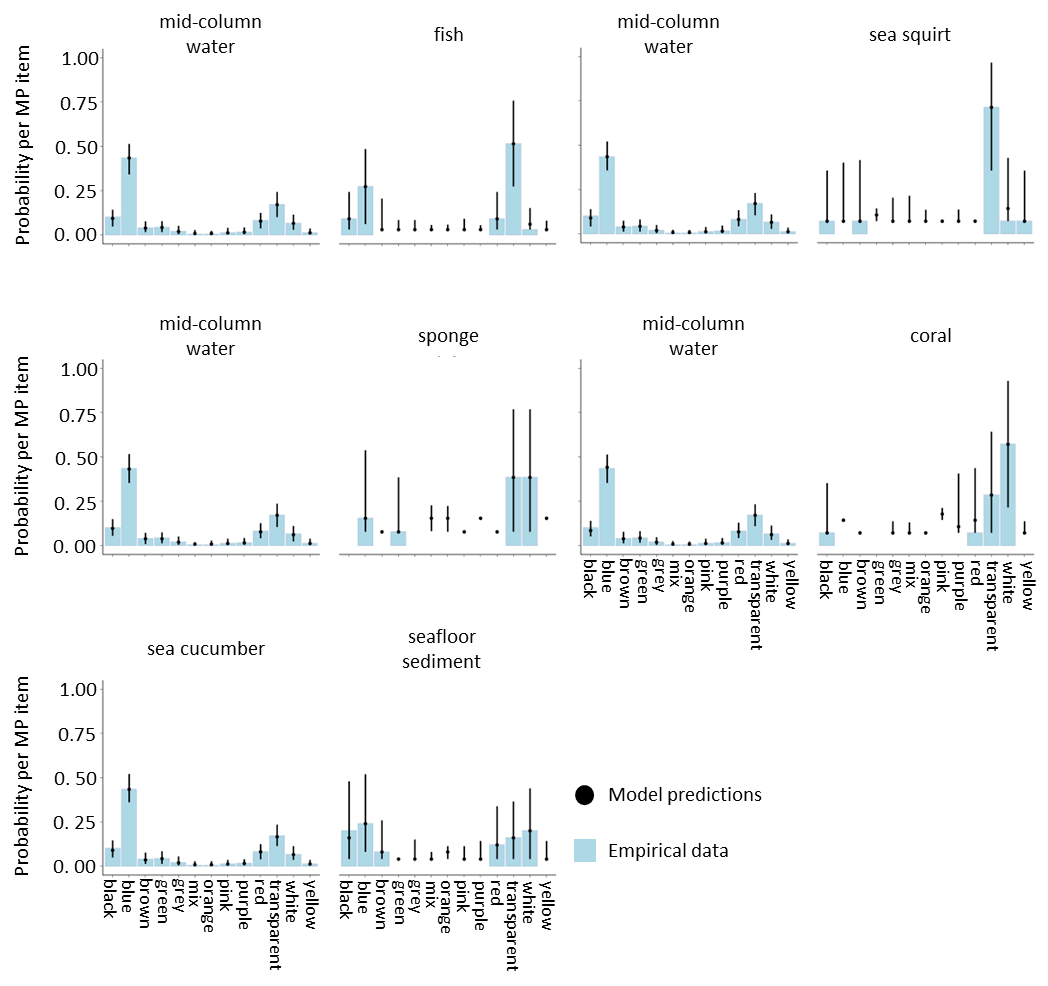


Figure S6: Bayesian regression model validation - Posterior predictive checks of proposed Eq. 3: $microplastic characteristic\sim0+Intercept+matrix$, with colour as microplastic characteristic. Relative frequency of occurrence of each microplastic colour in abiotic (left column) and biotic (right column) matrices. Blue bars represent the observed proportions, black point is the mean predicted proportion and horizontal error bars depicts the Bayesian 95% credible intervals. (A-D) illustrates posterior checks for mid-column water and (A) fish, (B) sea squirt, (C) sponge, (D) coral. (E) illustrates posterior checks for seafloor sediment and sea cucumber.


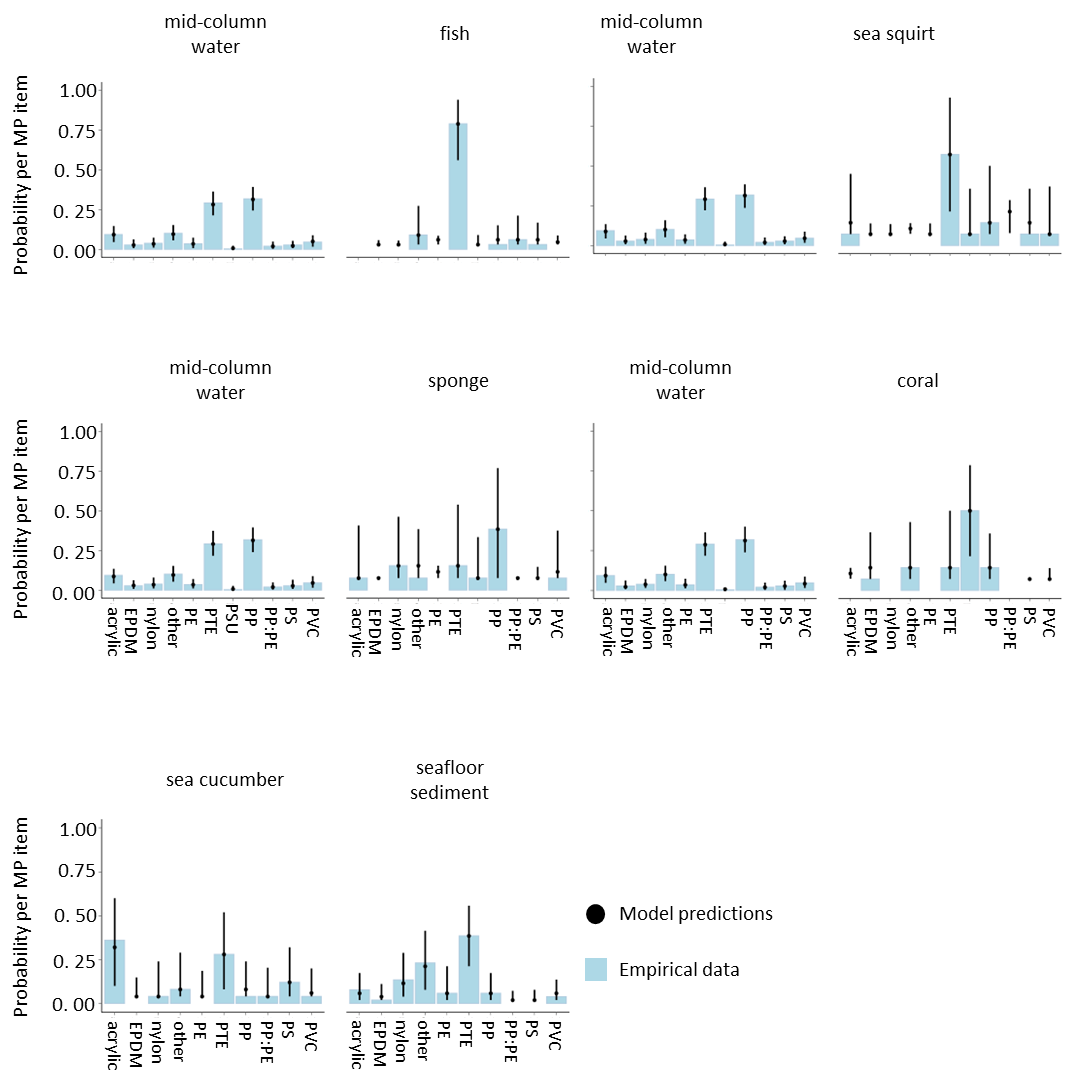


Figure S7: Bayesian regression model validation - Posterior predictive checks of proposed Eq. 3: $microplastic characteristic\sim0+Intercept+matrix$, with polymer type as microplastic characteristic. Relative frequency of occurrence of each microplastic colour in abiotic (left column) and biotic (right column) matrices. Blue bars represent the observed proportions, black point is the mean predicted proportion and horizontal error bars depicts the Bayesian 95% credible intervals. (A-D) illustrates posterior checks for mid-column water and (A) fish, (B) sea squirt, (C) sponge, (D) coral. (E) illustrates posterior checks for seafloor sediment and sea cucumber.

Supplementary Results

Environmental conditions

Table S6: Rainfall and air temperature during the period of field sample collection at Lizard Island. Parameters retrieved from the Australian Bureau of Meteorology (BOM) (Cape Flattery weather station, located 36.5 km from Lizard Island).

| Date (October 2018) | Rainfall (mm) | Temperature (˚C) |
| --- | --- | --- |
| 2 | 0 | 29.5 |
| 3 | 0 | 29.4 |
| 4 | 0 | 29.5 |
| 5 | 0 | 30.1 |
| 6 | 0 | 29.0 |
| 7 | 0 | 28.8 |
| 8 | 0 | 29.2 |
| 9 | 0 | 30.3 |
| 10 | 0 | 30.1 |
| 11 | 0 | 30.1 |
| 12 | 0 | 31.1 |


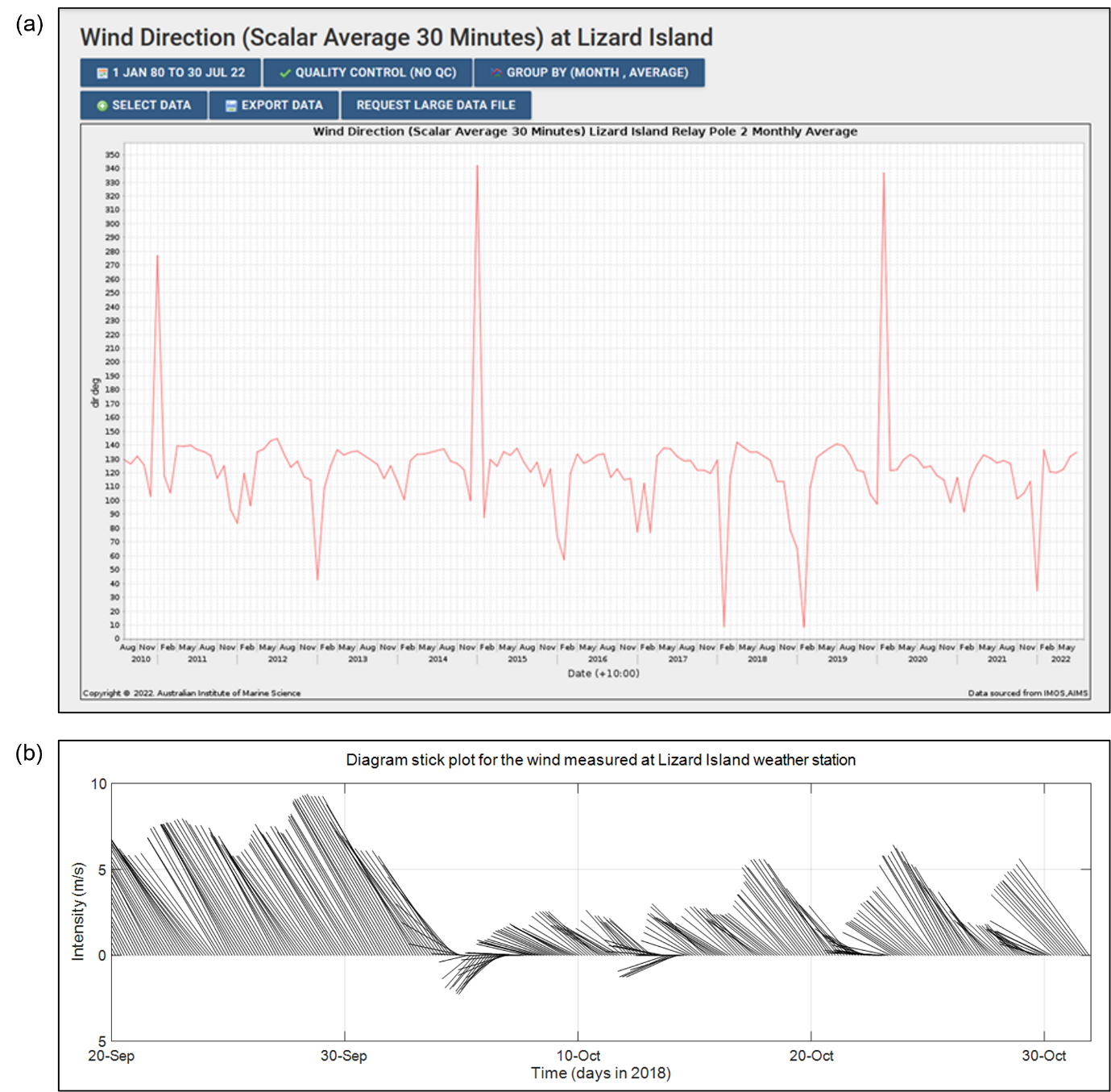


Figure S8: Diagram stick plot for the wind measured at Lizard Island weather station, including during the period of field sample collection (2^nd^ to 12^th^ October 2018), from the Australia’s Integrated Marine Observing System (IMOS). Original 10-minute interval data was smoothed by the moving mean (24 h) and plotted at regular 3 h intervals. Data accessed on 02-Apr-2022.

Table S5: Joint occurrence of intensity and direction of the wind at Lizard Island weather station, from the Australian Integrated Marine Observing System (IMOS), covering the months of September and October 2018 with 10-minute regular interval data. Analysis carried out from data reduced to 1 h regular intervals through moving mean. Direction is measured in degrees from geographical North, following Meteorological convention (“coming from”).


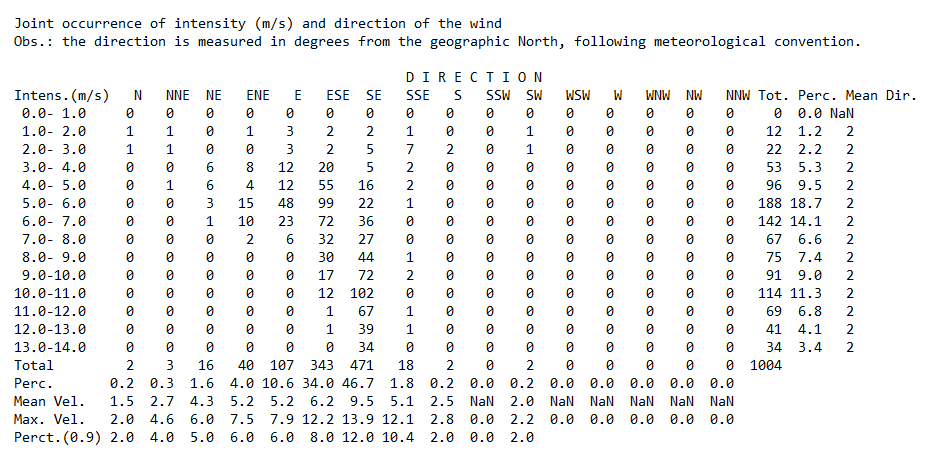


Data reliability

Table S7: Recovery rates (%, mean ± standard deviation, SD) from spike-recovery tests conducted for each matrix and respective microplastic separation method. Microplastics used were irregular particles (< 1.0 mm) of yellow polyethylene (PE) and of transparent polystyrene (PS), and monofilament fibres (approx. 2 mm) of black rayon (n = 5 per replicate, n = 3 replicates per spiked-recovery test). Sodium chloride = NaCl, potassium iodide = KI, nitric acid = HNO_3_.

| Sample type | Sample processing method | Spiked microplastic type | Recovery rate (%, mean ± SD) |
| --- | --- | --- | --- |
| Sea surface | Density separation  (1.2 g/cm^3^ NaCl) | PE | 100 ± 0 |
|  |  | PS | 93 ± 9 |
|  |  | Rayon | 73 ± 9 |
| Mid-column | Density separation  (1.7 g/cm^3^ KI) | PE | 100 ± 0 |
|  |  | PS | 93 ± 9 |
|  |  | Rayon | 93 ± 9 |
| Sediment | Density separation  (1.2 g/cm^3^ NaCl) | PE | 100 ± 0 |
|  |  | PS | 87 ± 9 |
|  |  | Rayon | 93 ± 9 |
| Fish | GIT visual sort | PE | 100 ± 0.00 |
|  |  | PS | 87 ± 9 |
|  |  | Rayon | 93 ± 9 |
| Sea squirt | Acid digestion  (70% HNO_3_) | PE | 100 ± 0 |
|  |  | PS | 87 ± 9 |
|  |  | Rayon | 73 ± 9 |
| Sponge | Acid digestion (70% HNO_3_) + Density separation  (1.7 g/cm^3^ KI) | PE | 93 ± 9 |
|  |  | PS | 93 ± 9 |
|  |  | Rayon | 67 ± 9 |
| Coral | Acid digestion  (70% HNO_3_) | PE | 100 ± 0 |
|  |  | PS | 87 ± 9 |
|  |  | Rayon | 80 ± 16 |
| Sea cucumber | Density separation  (1.7 g/cm^3^ KI) | PE | 100 ± 0 |
|  |  | PS | 80 ± 16 |
|  |  | Rayon | 100 ± 0 |

Table S8: Total number of putative microplastics isolated from all samples across the eight abiotic and biotic matrices, presented as: excluded items (i.e., items of poor spectra quality, flagged as contaminant, or of natural nature) and final microplastic count (i.e., semi-synthetic and synthetic items) for each individual abiotic and biotic matrix. GIT = gastrointestinal tract.

|  | | Abiotic matrix | | | Biotic matrix | | | | |
| --- | --- | --- | --- | --- | --- | --- | --- | --- | --- |
|  |  | Surface water | Mid-column water | Seafloor Sediment | Fish | Sea squirt | Sponge | Coral | Sea cucumber |
| Total putative microplastics | | 336 | 967 | 235 | 169 | 114 | 87 | 80 | 149 |
| Excluded items | poor spectra | 13 | 63 | 5 | 6 | 4 | 3 | 4 | 5 |
|  | contaminant | 34 | 106 | 14 | 16 | 11 | 12 | 7 | 21 |
|  | natural nature | 163 | 541 | 161 | 114 | 85 | 60 | 55 | 98 |
| Micro-plastics | semi-synthetic | 6 | 32 | 4 | 6 | 1 | 3 | 0 | 3 |
|  | synthetic | 121 | 226 | 48 | 27 | 13 | 10 | 14 | 22 |

Presence and abundance of microplastics at Lizard Island coral reefs

Table S9: Mean concentration (± standard deviation; SD) of microplastics per individual abiotic (microplastic m^-3^) and biotic (microplastic g^-1^) matrix from Granite Bluff and Blue Lagoon sampling sites (n = 5 replicates per sampling site). Concentration reported as number of microplastics m^-3^ (surface and mid-column waters, seafloor sediment) and number of microplastics g^-1^ of animal tissue processed (fish gastrointestinal tract (GIT), sea squirt innards, whole sponge, whole coral, and sea cucumber GIT).

| Sampling site | Abiotic | | | | Biotic | | | |
| --- | --- | --- | --- | --- | --- | --- | --- | --- |
|  | Surface water | Mid-column water | Seafloor Sediment | Fish GIT | Sea squirt | Sponge | Coral | Sea cucumber GIT |
| Granite Bluff | 0.21 ± 0.13 | 34.67 ± 17.97 | 794.57 ± 343.90 | 1.47 ± 1.04 | 0.95 ± 0.95 | 0.25 ± 0.13 | 0.03 ± 0.03 | 0.02 ± 0.02 |
| Blue Lagoon | 0.06 ± 0.03 | 22.67 ± 6.60 | 692.04 ± 376.70 | 0.44 ± 0.78 | 0.14 ± 0.20 | 0.08 ± 0.13 | 0.11 ± 0.07 | 0.01 ± 0.01 |

Table S10: Summary of general linear models (GLM) showing differences in microplastic concentration between (a) abiotic and (b) biotic matrices. Model for differences within abiotic matrices presented sampling site as a factor but not in interaction with sampling matrix. Intercept (i.e., reference) for this model is equal to seafloor sediment or Granite Bluff. Biotic matrices did not include sampling site as a factor and intercept is equal to fish. Significant differences were found when p < 0.05 and are marked with *.

(a) abiotic compartment: Adjusted R^2^ = 0.97, AIC = 74.19

|  | estimate | std. error | statistic | p-value |
| --- | --- | --- | --- | --- |
| Intercept | 6.6478 | 0.2436 | 27.2902 | < 2e^-16^ |
| mid-column | -3.0628 | 0.2983 | -10.2660 | 1.22e^-10*^ |
| surface | -8.6687 | 0.2983 | -29.0558 | < 2e^-16*^ |
| Blue Lagoon | -0.6884 | 0.2436 | -2.826 | 0.0089^*^ |

(b) biotic compartment: Adjusted R^2^ = 0.82, AIC = 58.39

| term | estimate | std. error | statistic | p-value |
| --- | --- | --- | --- | --- |
| Intercept | 0.7929 | 0.1258 | 6.304 | 1.1e-07 |
| coral | -0.5623 | 0.1779 | -3.161 | 0.0028^*^ |
| sea cucumber | -0.6981 | 0.1779 | -3.924 | 0.0003^*^ |
| sponge | -0.4635 | 0.1779 | -2.606 | 0.1238^*^ |
| sea squirt | -0.3042 | 0.1779 | -1.710 | 0.0941 |

Table S11: Total number of microplastics (i.e., counts) per matrix sampled. Columns report numbers by abiotic (surface and mid-column waters and seafloor sediment) and biotic (fish, sea squirt, sponge, coral and sea cucumber) matrices. Rows report total microplastic numbers per matrix sampled and per each physical and chemical characteristic examined: (a) shape, (b) size class, (c) colour, and (d) polymer type.

|  | surface water | mid-column water | Seafloor sediment | fish | sea squirt | sponge | coral | sea cucumber |
| --- | --- | --- | --- | --- | --- | --- | --- | --- |
| total | 139 | 258 | 52 | 33 | 14 | 13 | 14 | 25 |
| microplastic characteristic | | | | | | | | |
| (a) shape | | | | | | | | |
| fibre | 20 (16) | 115 (45) | 33 (63) | 25 (76) | 7 (50) | 4 (31) | 3 (21) | 12 (48) |
| fragment | 107 (84) | 143 (55) | 19 (37) | 8 (24) | 7 (50) | 9 (69) | 11 (79) | 13 (52) |
| (b) size |  | | | | | | | |
| class 1 (> 5 mm) | 4 (3) | 53 (21) | 0 | 0 | 0 | 0 | 2 (15) | 0 |
| class 2 (5 – 2.5 mm) | 15 (12) | 37 (14) | 2 (4) | 0 | 0 | 0 | 0 | 0 |
| class 3 (2.5 – 1 mm) | 43 (34) | 54 (21) | 9 (17) | 12 (36) | 3 (21) | 0 | 1 (7) | 7 (28) |
| class 4 (1 – 0.5 mm) | 16 (13) | 20 (8) | 17 (33) | 8 (24) | 3 (21) | 3 (23) | 2 (14) | 6 (24) |
| class 5 (< 0.5 mm) | 49 (38) | 94 (36) | 24 (46) | 13 (40) | 8 (58) | 10 (77) | 9 (64) | 12 (48) |
| (c) colour |  | | | | | | | |
| black | 9 (7) | 26 (10) | 5 (10) | 3 (9) | 1 (7) | 0 | 1 (7) | 5 (20) |
| blue | 33 (26) | 112 (43) | 19 (37) | 9 (27) | 0 | 2 (16) | 0 | 6 (24) |
| brown | 1 (< 1) | 10 (4) | 0 | 0 | 1 (7) | 0 | 0 | 2 (8) |
| green | 1 (< 1) | 11 (4) | 5 (10) | 0 | 0 | 1 (8) | 0 | 0 |
| grey | 1 (< 1) | 5 (2) | 0 | 0 | 0 | 0 | 0 | 0 |
| mix | 1 (< 1) | 1 (< 1) | 1 2) | 0 | 0 | 0 | 0 | 0 |
| orange | 0 | 1 (< 1) | 0 | 0 | 0 | 0 | 0 | 0 |
| pink | 32 (25) | 3 (1) | 2 (4) | 0 | 0 | 0 | 0 | 0 |
| purple | 0 | 4 (2) | 0 | 0 | 0 | 0 | 0 | 0 |
| red | 5 (4) | 21 (8) | 5 (10) | 3 (9) | 0 | 0 | 1 (7) | 3 (12) |
| transparent | 15 (12) | 44 (17) | 11 (21) | 17 (52) | 10 (72) | 5 (38) | 4 (29) | 4 (16) |
| white | 27 (21) | 17 (7) | 1 (2) | 1 (3) | 1 (7) | 5 (38) | 8 (57) | 5 (20) |
| yellow | 2 (2) | 3 (1) | 3(6) | 0 | 1 (7) | 0 | 0 | 0 |
| (d) polymer | | | | | | | | |
| acrylic | 20 (16) | 24 (9) | 4 (8) | 0 | 1 (7) | 1 (8) | 0 | 9 (36) |
| EPDM | 4 (3) | 7 (3) | 1 (2) | 0 | 0 | 0 | 1 (7) | 0 |
| nylon | 1 (1) | 10 (3) | 7 (13) | 0 | 0 | 2 (15) | 0 | 1 (4) |
| PE | 34 (27) | 9 (3) | 3 (6) | 0 | 0 | 0 | 0 | 0 |
| PET | 12 (9) | 75 (29) | 20 (38) | 26 (79) | 8 (57) | 2 (15) | 2 (14) | 7 (28) |
| PSU | 0 | 1 (<1) | 0 | 0 | 1 (7) | 1 (8) | 7 (50) | 0 |
| PP | 45 (35) | 82 (32) | 3 (6) | 1 (3) | 2 (15) | 5 (38) | 2 (14) | 1 (4) |
| PP:PE | 7 (6) | 5 (2) | 0 | 2 (6) | 0 | 0 | 0 | 1 (4) |
| PS | 0 | 7 (3) | 0 | 1 (3) | 1 (7) | 0 | 0 | 3 (12) |
| PVC | 1 (1) | 12 (5) | 2 (4) | 0 | 1 (7) | 2 (8) | 0 | 1 (4) |
| other polymers | 3 (2) | 26 (10) | 12 (23) | 3 (9) | 0 | 1 (8) | 2 (14) | 2 (8) |

Table S12: Summary data for the Bayesian regression model (a) and respective posterior distributions (b) analysing differences and associated uncertainty of microplastic shape between mid-column water and fish. Number of observations 291. CI = credible interval. Rhat = scale reduction factor. ESS = effective sample size.

(a) Bayesian model summary

| Effects | Estimate | Est.Error | l-95% CI | u-95% CI | Rhat | Bulk_ESS | Tail_ESS |
| --- | --- | --- | --- | --- | --- | --- | --- |
| intercept | 0.22 | 0.13 | -0.03 | 0.47 | 1.00 | 3667.05 | 2083.07 |
| fragment_fish | -1.39 | 0.44 | -2.30 | -0.58 | 1.00 | 3444.86 | 2795.31 |

(b) Bayesian posterior distribution in differences. Estimate represents the estimated average difference. Lower and upper CI represent the estimated 95% credible intervals.

| shape | mean_diff | lower_ci | upper_ci |
| --- | --- | --- | --- |
| fibre | -0.31 | -0.46 | -0.14 |
| fragment | 0.31 | 0.14 | 0.46 |

Table S13: Summary data for the Bayesian regression model (a) and respective posterior distributions (b) analysing differences and associated uncertainty of microplastic shape between mid-column water and sea squirt. Number of observations 272. CI = credible interval. Rhat = scale reduction factor. ESS = effective sample size.

(a) Bayesian model summary.

| Effects | Estimate | Est.Error | l-95% CI | u-95% CI | Rhat | Bulk_ESS | Tail_ESS |
| --- | --- | --- | --- | --- | --- | --- | --- |
| intercept | 0.22 | 0.13 | -0.04 | 0.46 | 1.00 | 3639.82 | 2712.83 |
| fragment_ssquirt | -0.21 | 0.56 | -1.28 | 0.88 | 1.00 | 3916.16 | 2851.51 |

(b) Bayesian posterior distribution in differences. Estimate represents the estimated average difference. Lower and upper CI represent the estimated 95% credible intervals.

| shape | mean_diff | lower_ci | upper_ci |
| --- | --- | --- | --- |
| fibre | 0.05 | -0.20 | 0.30 |
| fragment | -0.05 | -0.30 | 0.20 |

Table S14: Summary data for the Bayesian regression model (a) and respective posterior distributions (b) analysing differences and associated uncertainty of microplastic shape between mid-column water and sponge. Number of observations 271. CI = credible interval. Rhat = scale reduction factor. ESS = effective sample size.

(a) Bayesian model summary.

| Effects | Estimate | Est.Error | l-95% CI | u-95% CI | Rhat | Bulk_ESS | Tail_ESS |
| --- | --- | --- | --- | --- | --- | --- | --- |
| intercept | 0.22 | 0.13 | -0.03 | 0.46 | 1.00 | 3930.27 | 2864.24 |
| fragment_sponge | 0.67 | 0.64 | -0.55 | 2.00 | 1.00 | 3711.63 | 2355.48 |

(b) Bayesian posterior distribution in differences. Estimate represents the estimated average difference. Lower and upper CI represent the estimated 95% credible intervals.

| shape | mean_diff | lower_ci | upper_ci |
| --- | --- | --- | --- |
| fibre | -0.14 | -0.36 | 0.14 |
| fragment | 0.14 | -0.14 | 0.36 |

Table S15: Summary data for the Bayesian regression model (a) and respective posterior distributions (b) analysing differences and associated uncertainty of microplastic shape between mid-column water and coral. Number of observations 272. CI = credible interval. Rhat = scale reduction factor. ESS = effective sample size.

(a) Bayesian model summary.

| Effects | Estimate | Est.Error | l-95% CI | u-95% CI | Rhat | Bulk_ESS | Tail_ESS |
| --- | --- | --- | --- | --- | --- | --- | --- |
| intercept | 0.22 | 0.12 | -0.01 | 0.47 | 1.00 | 2975.23 | 2599.95 |
| fragment_coral | 1.24 | 0.73 | -0.03 | 2.75 | 1.00 | 2652.74 | 2182.27 |

(b) Bayesian posterior distribution in differences. Estimate represents the estimated average difference. Lower and upper CI represent the estimated 95% credible intervals.

| shape | mean_diff | lower_ci | upper_ci |
| --- | --- | --- | --- |
| fibre | -0.24 | -0.41 | 0.01 |
| fragment | 0.24 | -0.01 | 0.41 |

Table S16: Summary data for the Bayesian regression model (a) and respective posterior distributions (b) analysing differences and associated uncertainty of microplastic shape between seafloor sediment and sea cucumber. Number of observations 77. CI = credible interval. Rhat = scale reduction factor. ESS = effective sample size.

(a) Bayesian model summary.

| Effects | Estimate | Est.Error | l-95% CI | u-95% CI | Rhat | Bulk_ESS | Tail_ESS |
| --- | --- | --- | --- | --- | --- | --- | --- |
| intercept | 0.09 | 0.40 | -0.68 | 0.87 | 1.00 | 3653.37 | 2607.82 |
| fragment_sediment | -0.65 | 0.48 | -1.59 | 0.29 | 1.00 | 3636.28 | 2548.47 |

(b) Bayesian posterior distribution in differences. Estimate represents the estimated average difference. Lower and upper CI represent the estimated 95% credible intervals.

| shape | mean_diff | lower_ci | upper_ci |
| --- | --- | --- | --- |
| fibre | -0.15 | -0.37 | 0.07 |
| fragment | 0.15 | -0.07 | 0.37 |

Table S17: Summary data for the Bayesian regression model (a) and respective posterior distributions (b) analysing differences and associated uncertainty of microplastic size class between mid-column water and fish. Number of observations 291. CI = credible interval. Rhat = scale reduction factor. ESS = effective sample size.

(a) Bayesian model summary.

| Effects | Estimate | Est.Error | l-95% CI | u-95% CI | Rhat | Bulk_ESS | Tail_ESS |
| --- | --- | --- | --- | --- | --- | --- | --- |
| 2_Intercept | -0.36 | 0.21 | -0.77 | 0.05 | 1.00 | 3826.33 | 3025.04 |
| 3_Intercept | 0.01 | 0.19 | -0.37 | 0.38 | 1.00 | 3751.33 | 2955.40 |
| 4_Intercept | -1.00 | 0.27 | -1.55 | -0.49 | 1.00 | 3217.93 | 2517.81 |
| 5_Intercept | 0.57 | 0.17 | 0.23 | 0.91 | 1.00 | 4267.75 | 3175.44 |
| 2_fish | -21.35 | 38.92 | -125.16 | 15.19 | 1.02 | 268.71 | 183.49 |
| 3_fish | 13.55 | 8.93 | 2.83 | 36.93 | 1.01 | 351.64 | 363.03 |
| 4_fish | 14.14 | 8.93 | 3.46 | 37.47 | 1.01 | 352.54 | 355.21 |
| 5_fish | 13.07 | 8.93 | 2.35 | 36.58 | 1.01 | 352.55 | 359.17 |

(b) Bayesian posterior distribution in differences. Estimate represents the estimated average difference. Lower and upper CI represent the estimated 95% credible intervals.

| size_range | mean_diff | lower_ci | upper_ci |
| --- | --- | --- | --- |
| 1 | 0.20 | 0.15 | 0.26 |
| 2 | 0.14 | 0.10 | 0.19 |
| 3 | -0.15 | -0.33 | 0.01 |
| 4 | -0.17 | -0.32 | -0.03 |
| 5 | -0.03 | -0.21 | 0.14 |

Table S18: Summary data for the Bayesian regression model (a) and respective posterior distributions (b) analysing differences and associated uncertainty of microplastic size class between mid-column water and sea squirt. Number of observations 272. CI = credible interval. Rhat = scale reduction factor. ESS = effective sample size.

(a) Bayesian model summary.

| Effects | Estimate | Est.Error | l-95% CI | u-95% CI | Rhat | Bulk_ESS | Tail_ESS |
| --- | --- | --- | --- | --- | --- | --- | --- |
| 2_Intercept | -0.36 | 0.21 | -0.79 | 0.04 | 1.00 | 4061.47 | 3063.91 |
| 3_Intercept | 0.01 | 0.19 | -0.36 | 0.39 | 1.00 | 3959.51 | 2653.22 |
| 4_Intercept | -1.01 | 0.27 | -1.53 | -0.50 | 1.00 | 3173.45 | 2570.59 |
| 5_Intercept | 0.57 | 0.17 | 0.23 | 0.91 | 1.00 | 3495.01 | 2866.37 |
| 2_ssquirt | -35.08 | 55.14 | -189.91 | 35.32 | 1.00 | 380.21 | 376.76 |
| 3_ssquirt | 27.32 | 18.93 | 2.40 | 72.09 | 1.01 | 334.18 | 425.45 |
| 4_ssquirt | 28.34 | 18.95 | 3.11 | 73.60 | 1.01 | 333.93 | 432.97 |
| 5_ssquirt | 27.87 | 18.93 | 2.88 | 73.18 | 1.01 | 332.09 | 423.84 |

(b) Bayesian posterior distribution in differences. Estimate represents the estimated average difference. Lower and upper CI represent the estimated 95% credible intervals.

| size_range | mean_diff | lower_ci | upper_ci |
| --- | --- | --- | --- |
| 1 | 0.21 | 0.16 | 0.26 |
| 2 | 0.14 | 0.10 | 0.19 |
| 3 | 0.00 | -0.25 | 0.17 |
| 4 | -0.14 | -0.38 | 0.03 |
| 5 | -0.21 | -0.45 | 0.06 |

Table S19: Summary data for the Bayesian regression model (a) and respective posterior distributions (b) analysing differences and associated uncertainty of microplastic size class between mid-column water and sponge. Number of observations 271. CI = credible interval. Rhat = scale reduction factor. ESS = effective sample size.

(a) Bayesian model summary.

| Effects | Estimate | Est.Error | l-95% CI | u-95% CI | Rhat | Bulk_ESS | Tail_ESS |
| --- | --- | --- | --- | --- | --- | --- | --- |
| 2_Intercept | -0.36 | 0.22 | -0.78 | 0.07 | 1.00 | 3732.72 | 3040.71 |
| 3_Intercept | 0.02 | 0.19 | -0.36 | 0.39 | 1.00 | 4201.97 | 3358.46 |
| 4_Intercept | -1.01 | 0.26 | -1.52 | -0.50 | 1.00 | 3726.05 | 2532.92 |
| 5_Intercept | 0.57 | 0.17 | 0.23 | 0.91 | 1.00 | 3814.84 | 2844.58 |
| 2_sponge | -26.48 | 62.14 | -194.95 | 57.50 | 1.01 | 435.49 | 272.91 |
| 3_sponge | -29.80 | 56.23 | -162.76 | 52.96 | 1.01 | 477.07 | 502.83 |
| 4_sponge | 45.23 | 32.53 | 4.85 | 128.82 | 1.02 | 235.76 | 389.77 |
| 5_sponge | 44.99 | 32.53 | 4.40 | 128.40 | 1.02 | 236.83 | 399.90 |

(b) Bayesian posterior distribution in differences. Estimate represents the estimated average difference. Lower and upper CI represent the estimated 95% credible intervals.

| size_range | mean_diff | lower_ci | upper_ci |
| --- | --- | --- | --- |
| 1 | 0.21 | 0.16 | 0.26 |
| 2 | 0.14 | 0.10 | 0.19 |
| 3 | 0.21 | 0.16 | 0.26 |
| 4 | -0.15 | -0.41 | 0.03 |
| 5 | -0.41 | -0.60 | -0.14 |

Table S20: Summary data for the Bayesian regression model (a) and respective posterior distributions (b) analysing differences and associated uncertainty of microplastic size class between mid-column water and coral. Number of observations 272. CI = credible interval. Rhat = scale reduction factor. ESS = effective sample size.

(a) Bayesian model summary.

| Effects | Estimate | Est.Error | l-95% CI | u-95% CI | Rhat | Bulk_ESS | Tail_ESS |
| --- | --- | --- | --- | --- | --- | --- | --- |
| 2_Intercept | -0.34 | 0.22 | -0.78 | 0.09 | 1.00 | 2225.83 | 2512.49 |
| 3_Intercept | 0.03 | 0.20 | -0.37 | 0.42 | 1.00 | 1398.63 | 2087.10 |
| 4_Intercept | -0.97 | 0.27 | -1.52 | -0.47 | 1.00 | 1434.94 | 1770.19 |
| 5_Intercept | 0.59 | 0.17 | 0.25 | 0.94 | 1.00 | 1283.59 | 1478.48 |
| 2_coral | -46.83 | 48.61 | -175.75 | -1.81 | 1.01 | 799.14 | 544.67 |
| 3_coral | -1.01 | 1.48 | -4.25 | 1.63 | 1.00 | 1372.71 | 1270.15 |
| 4_coral | 1.01 | 1.15 | -1.21 | 3.22 | 1.00 | 1396.27 | 1932.53 |
| 5_coral | 1.15 | 0.88 | -0.35 | 3.06 | 1.00 | 1457.06 | 1702.94 |

(b) Bayesian posterior distribution in differences. Estimate represents the estimated average difference. Lower and upper CI represent the estimated 95% credible intervals.

| size_range | mean_diff | lower_ci | upper_ci |
| --- | --- | --- | --- |
| 1 | 0.06 | -0.15 | 0.20 |
| 2 | 0.14 | 0.10 | 0.19 |
| 3 | 0.14 | -0.03 | 0.23 |
| 4 | -0.07 | -0.28 | 0.06 |
| 5 | -0.28 | -0.51 | -0.02 |

Table S21: Summary data for the Bayesian regression model (a) and respective posterior distributions (b) analysing differences and associated uncertainty of microplastic size class between seafloor sediment and sea cucumber. Number of observations 77. CI = credible interval. Rhat = scale reduction factor. ESS = effective sample size.

(a) Bayesian model summary.

| Effects | Estimate | Est.Error | l-95% CI | u-95% CI | Rhat | Bulk_ESS | Tail_ESS |
| --- | --- | --- | --- | --- | --- | --- | --- |
| 3_Intercept | 4.27 | 2.44 | 0.90 | 10.55 | 1.01 | 535.01 | 397.38 |
| 4_Intercept | 4.09 | 2.44 | 0.79 | 10.31 | 1.01 | 553.54 | 430.35 |
| 5_Intercept | 4.84 | 2.42 | 1.60 | 11.13 | 1.01 | 543.32 | 406.01 |
| 3_sediment | -2.99 | 2.63 | -9.44 | 0.99 | 1.01 | 572.79 | 369.03 |
| 4_sediment | -2.14 | 2.61 | -8.68 | 1.76 | 1.01 | 567.51 | 391.31 |
| 5_sediment | -2.53 | 2.59 | -9.10 | 1.21 | 1.01 | 571.31 | 422.70 |

(b) Bayesian posterior distribution in differences. Estimate represents the estimated average difference. Lower and upper CI represent the estimated 95% credible intervals.

| size_range | mean_diff | lower_ci | upper_ci |
| --- | --- | --- | --- |
| 1 | 0.04 | -0.05 | 0.13 |
| 2 | -0.11 | -0.31 | 0.08 |
| 3 | 0.09 | -0.12 | 0.28 |
| 4 | -0.02 | -0.25 | 0.21 |

Table S22: Summary data for the Bayesian regression model (a) and respective posterior distributions (b) analysing differences and associated uncertainty of microplastic colour between mid-column water and fish. Number of observations 291. CI = credible interval. Rhat = scale reduction factor. ESS = effective sample size.

(a) Bayesian model summary.

| Effects | Estimate | Est.Error | l-95% CI | u-95% CI | Rhat | Bulk_ESS | Tail_ESS |
| --- | --- | --- | --- | --- | --- | --- | --- |
| blue_Intercept | 1.58 | 0.23 | 1.15 | 2.03 | 1.00 | 2048.15 | 2723.42 |
| brown_Intercept | -0.84 | 0.37 | -1.61 | -0.14 | 1.00 | 4399.01 | 5066.91 |
| green_Intercept | -0.75 | 0.37 | -1.51 | -0.08 | 1.00 | 4237.18 | 4779.04 |
| grey_Intercept | -1.55 | 0.50 | -2.60 | -0.66 | 1.00 | 5570.98 | 5443.79 |
| mix_Intercept | -3.21 | 1.04 | -5.62 | -1.56 | 1.00 | 5608.23 | 4266.70 |
| orange_Intercept | -3.25 | 1.07 | -5.84 | -1.58 | 1.00 | 5651.35 | 4219.06 |
| pink_Intercept | -2.08 | 0.62 | -3.44 | -1.00 | 1.00 | 5510.36 | 4141.52 |
| purple_Intercept | -1.79 | 0.55 | -2.96 | -0.79 | 1.00 | 5587.73 | 5165.28 |
| red_Intercept | -0.11 | 0.30 | -0.70 | 0.47 | 1.00 | 2567.45 | 3524.05 |
| transparent_Intercept | 0.64 | 0.26 | 0.14 | 1.14 | 1.00 | 2199.37 | 3301.24 |
| white_Intercept | -0.32 | 0.32 | -0.95 | 0.28 | 1.00 | 3047.92 | 4151.24 |
| yellow_Intercept | -2.08 | 0.63 | -3.45 | -0.99 | 1.00 | 5329.74 | 4499.71 |
| blue_fish | -0.24 | 0.79 | -1.66 | 1.51 | 1.00 | 1851.60 | 2054.61 |
| brown_fish | -24.79 | 33.33 | -98.33 | -0.60 | 1.00 | 806.24 | 351.62 |
| green_fish | -24.11 | 25.65 | -93.99 | -0.73 | 1.01 | 1199.34 | 734.98 |
| grey_fish | -22.79 | 27.23 | -95.02 | 0.32 | 1.00 | 1329.41 | 643.43 |
| mix_fish | -22.00 | 28.98 | -101.45 | 2.27 | 1.01 | 1910.78 | 1156.93 |
| orange_fish | -23.92 | 33.45 | -115.53 | 2.21 | 1.00 | 941.91 | 591.81 |
| pink_fish | -23.00 | 32.00 | -101.40 | 0.92 | 1.00 | 1030.27 | 577.21 |
| purple_fish | -21.20 | 26.44 | -85.13 | 0.56 | 1.00 | 1944.35 | 1197.75 |
| red_fish | 0.24 | 0.97 | -1.62 | 2.22 | 1.00 | 2162.77 | 2551.91 |
| transparent_fish | 1.38 | 0.77 | 0.03 | 3.10 | 1.00 | 1828.84 | 2038.87 |
| white_fish | -1.03 | 1.47 | -4.19 | 1.52 | 1.00 | 2306.77 | 3259.25 |
| yellow_fish | -23.57 | 31.71 | -115.45 | 0.86 | 1.01 | 749.59 | 272.06 |

(b) Bayesian posterior distribution in differences. Estimate represents the estimated average difference. Lower and upper CI represent the estimated 95% credible intervals.

| colour | mean_diff | lower_ci | upper_ci |
| --- | --- | --- | --- |
| black | 0.01 | -0.11 | 0.09 |
| blue | 0.16 | -0.01 | 0.31 |
| brown | 0.04 | 0.02 | 0.07 |
| green | 0.04 | 0.02 | 0.07 |
| grey | 0.02 | 0.00 | 0.04 |
| mix | 0.00 | -0.01 | 0.02 |
| orange | 0.00 | -0.01 | 0.02 |
| pink | 0.01 | -0.01 | 0.03 |
| purple | 0.02 | 0.00 | 0.04 |
| red | -0.01 | -0.13 | 0.07 |
| transparent | -0.35 | -0.52 | -0.17 |
| white | 0.04 | -0.05 | 0.09 |
| yellow | 0.01 | 0.00 | 0.03 |

Table S23: Summary data for the Bayesian regression model (a) and respective posterior distributions (b) analysing differences and associated uncertainty of microplastic colour between mid-column water and sea squirt. Number of observations 272. CI = credible interval. Rhat = scale reduction factor. ESS = effective sample size.

(a) Bayesian model summary.

| Effects | Estimate | Est.Error | l-95% CI | u-95% CI | Rhat | Bulk_ESS | Tail_ESS |
| --- | --- | --- | --- | --- | --- | --- | --- |
| blue_Intercept | 1.61 | 0.23 | 1.18 | 2.07 | 1.00 | 2082.32 | 2461.19 |
| brown_Intercept | -0.84 | 0.38 | -1.61 | -0.14 | 1.00 | 2386.25 | 2398.04 |
| green_Intercept | -0.72 | 0.37 | -1.45 | -0.01 | 1.00 | 3052.20 | 2978.59 |
| grey_Intercept | -1.53 | 0.49 | -2.56 | -0.63 | 1.00 | 3707.01 | 3271.52 |
| mix_Intercept | -3.19 | 1.07 | -5.67 | -1.54 | 1.00 | 3074.23 | 2302.68 |
| orange_Intercept | -3.23 | 1.10 | -5.77 | -1.55 | 1.00 | 3195.12 | 2095.77 |
| pink_Intercept | -2.05 | 0.60 | -3.33 | -0.98 | 1.00 | 3673.07 | 2520.49 |
| purple_Intercept | -1.75 | 0.55 | -2.95 | -0.76 | 1.00 | 3352.32 | 2853.75 |
| red_Intercept | -0.08 | 0.30 | -0.67 | 0.52 | 1.00 | 2619.04 | 2834.23 |
| transparent_Intercept | 0.66 | 0.25 | 0.17 | 1.17 | 1.00 | 2126.54 | 2287.66 |
| white_Intercept | -0.30 | 0.32 | -0.92 | 0.31 | 1.00 | 1986.08 | 2096.14 |
| yellow_Intercept | -2.07 | 0.61 | -3.38 | -0.97 | 1.00 | 2379.65 | 1970.22 |
| blue_ssquirt | -70.71 | 75.47 | -246.67 | -3.85 | 1.01 | 425.14 | 183.42 |
| brown_ssquirt | 1.28 | 2.10 | -2.58 | 5.70 | 1.00 | 880.36 | 692.45 |
| green_ssquirt | -49.71 | 55.60 | -197.10 | 0.25 | 1.01 | 614.18 | 396.00 |
| grey_ssquirt | -45.20 | 52.41 | -188.50 | 1.39 | 1.00 | 1143.18 | 907.33 |
| mix_ssquirt | -63.92 | 108.93 | -339.47 | 3.00 | 1.01 | 418.44 | 163.47 |
| orange_ssquirt | -55.39 | 75.68 | -256.52 | 3.21 | 1.00 | 704.78 | 360.26 |
| pink_ssquirt | -61.05 | 100.90 | -407.00 | 1.55 | 1.01 | 352.64 | 160.19 |
| purple_ssquirt | -43.30 | 48.21 | -169.17 | 1.45 | 1.00 | 1159.14 | 982.92 |
| red_ssquirt | -52.97 | 56.28 | -202.20 | -1.00 | 1.00 | 698.12 | 430.64 |
| transparent_ssquirt | 2.56 | 1.69 | 0.18 | 6.59 | 1.01 | 751.09 | 599.85 |
| white_ssquirt | 0.72 | 2.12 | -3.19 | 5.12 | 1.01 | 873.95 | 784.14 |
| yellow_ssquirt | 2.49 | 2.18 | -1.51 | 7.08 | 1.01 | 809.11 | 710.81 |

(b) Bayesian posterior distribution in differences. Estimate represents the estimated average difference. Lower and upper CI represent the estimated 95% credible intervals.

| colour | mean_diff | lower_ci | upper_ci |
| --- | --- | --- | --- |
| black | 0.03 | -0.14 | 0.11 |
| blue | 0.44 | 0.38 | 0.49 |
| brown | -0.03 | -0.21 | 0.05 |
| green | 0.04 | 0.02 | 0.07 |
| grey | 0.02 | 0.00 | 0.04 |
| mix | 0.00 | -0.01 | 0.02 |
| orange | 0.00 | -0.01 | 0.02 |
| pink | 0.01 | 0.00 | 0.03 |
| purple | 0.02 | 0.00 | 0.04 |
| red | 0.08 | 0.05 | 0.12 |
| transparent | -0.54 | -0.75 | -0.28 |
| white | -0.01 | -0.19 | 0.08 |
| yellow | -0.06 | -0.24 | 0.01 |

Table S24: Summary data for the Bayesian regression model (a) and respective posterior distributions (b) analysing differences and associated uncertainty of microplastic colour between mid-column water and sponge. Number of observations 271. CI = credible interval. Rhat = scale reduction factor. ESS = effective sample size.

(a) Bayesian model summary.

| Effects | Estimate | Est.Error | l-95% CI | u-95% CI | Rhat | Bulk_ESS | Tail_ESS |
| --- | --- | --- | --- | --- | --- | --- | --- |
| blue_Intercept | 1.47 | 0.22 | 1.06 | 1.93 | 1.00 | 4308.42 | 3522.08 |
| brown_Intercept | -0.98 | 0.38 | -1.74 | -0.27 | 1.00 | 3940.77 | 3602.58 |
| green_Intercept | -0.92 | 0.37 | -1.70 | -0.23 | 1.00 | 4680.17 | 3363.65 |
| grey_Intercept | -1.71 | 0.53 | -2.84 | -0.79 | 1.00 | 3889.76 | 2964.11 |
| mix_Intercept | -3.42 | 1.09 | -6.04 | -1.71 | 1.00 | 3363.03 | 2654.57 |
| orange_Intercept | -3.44 | 1.11 | -6.10 | -1.74 | 1.00 | 3903.57 | 2712.00 |
| pink_Intercept | -2.24 | 0.64 | -3.65 | -1.11 | 1.00 | 3965.37 | 2791.52 |
| purple_Intercept | -1.92 | 0.54 | -3.07 | -0.92 | 1.00 | 3878.80 | 3025.11 |
| red_Intercept | -0.22 | 0.29 | -0.79 | 0.35 | 1.00 | 4208.37 | 3333.48 |
| transparent_Intercept | 0.52 | 0.25 | 0.04 | 1.03 | 1.00 | 4282.36 | 3728.12 |
| white_Intercept | -0.46 | 0.32 | -1.09 | 0.16 | 1.00 | 4571.70 | 3964.02 |
| yellow_Intercept | -2.24 | 0.65 | -3.69 | -1.17 | 1.00 | 4036.78 | 2549.82 |
| blue_sponge | 65.34 | 26.88 | 16.18 | 125.00 | 1.01 | 712.82 | 914.32 |
| brown_sponge | -15.77 | 77.06 | -213.18 | 73.28 | 1.00 | 778.18 | 419.08 |
| green_sponge | 66.69 | 26.93 | 17.41 | 126.83 | 1.01 | 712.22 | 869.26 |
| grey_sponge | -7.47 | 87.35 | -171.98 | 77.44 | 1.00 | 939.45 | 507.30 |
| mix_sponge | -1.41 | 99.22 | -242.72 | 91.72 | 1.01 | 471.32 | 235.74 |
| orange_sponge | 2.02 | 72.68 | -199.52 | 91.87 | 1.01 | 685.27 | 355.28 |
| pink_sponge | 2.33 | 61.87 | -149.96 | 83.28 | 1.00 | 998.18 | 764.46 |
| purple_sponge | -0.16 | 61.76 | -161.16 | 79.74 | 1.01 | 454.73 | 464.23 |
| red_sponge | -21.76 | 73.00 | -175.89 | 67.97 | 1.01 | 989.74 | 515.61 |
| transparent_sponge | 67.39 | 26.87 | 18.22 | 127.34 | 1.01 | 714.47 | 828.34 |
| white_sponge | 68.36 | 26.88 | 19.44 | 127.93 | 1.01 | 713.88 | 830.25 |
| yellow_sponge | 4.71 | 56.67 | -143.41 | 81.99 | 1.00 | 1256.06 | 869.83 |

(b) Bayesian posterior distribution in differences. Estimate represents the estimated average difference. Lower and upper CI represent the estimated 95% credible intervals.

| colour | mean_diff | lower_ci | upper_ci |
| --- | --- | --- | --- |
| black | 0.10 | 0.07 | 0.14 |
| blue | 0.28 | 0.04 | 0.43 |
| brown | 0.04 | 0.02 | 0.07 |
| green | -0.03 | -0.23 | 0.05 |
| grey | 0.02 | 0.01 | 0.04 |
| mix | 0.00 | 0.00 | 0.02 |
| orange | 0.00 | -0.01 | 0.02 |
| pink | 0.01 | 0.00 | 0.03 |
| purple | 0.02 | 0.00 | 0.03 |
| red | 0.08 | 0.05 | 0.12 |
| transparent | -0.22 | -0.49 | 0.02 |
| white | -0.32 | -0.59 | -0.08 |
| yellow | 0.01 | 0.00 | 0.03 |

Table S25: Summary data for the Bayesian regression model (a) and respective posterior distributions (b) analysing differences and associated uncertainty of microplastic colour between mid-column water and coral. Number of observations 272. CI = credible interval. Rhat = scale reduction factor. ESS = effective sample size.

(a) Bayesian model summary.

| Effects | Estimate | Est.Error | l-95% CI | u-95% CI | Rhat | Bulk_ESS | Tail_ESS |
| --- | --- | --- | --- | --- | --- | --- | --- |
| blue_Intercept | 1.61 | 0.23 | 1.18 | 2.07 | 1.00 | 2282.33 | 2662.84 |
| brown_Intercept | -0.82 | 0.38 | -1.59 | -0.11 | 1.00 | 2916.30 | 2979.00 |
| green_Intercept | -0.73 | 0.36 | -1.46 | -0.05 | 1.00 | 3328.86 | 3183.93 |
| grey_Intercept | -1.54 | 0.49 | -2.56 | -0.66 | 1.00 | 3235.62 | 2966.56 |
| mix_Intercept | -3.18 | 1.01 | -5.56 | -1.58 | 1.00 | 3233.27 | 2279.52 |
| orange_Intercept | -3.20 | 1.04 | -5.63 | -1.59 | 1.00 | 3043.15 | 1933.94 |
| pink_Intercept | -2.04 | 0.63 | -3.40 | -0.95 | 1.00 | 4093.22 | 2451.41 |
| purple_Intercept | -1.75 | 0.54 | -2.88 | -0.77 | 1.00 | 3546.74 | 3122.32 |
| red_Intercept | -0.09 | 0.30 | -0.69 | 0.49 | 1.00 | 2434.55 | 2275.47 |
| transparent_Intercept | 0.66 | 0.25 | 0.16 | 1.15 | 1.00 | 2161.32 | 2325.50 |
| white_Intercept | -0.31 | 0.32 | -0.97 | 0.31 | 1.00 | 2173.33 | 2255.25 |
| yellow_Intercept | -2.04 | 0.61 | -3.32 | -0.96 | 1.00 | 3688.77 | 2701.46 |
| blue_coral | -68.16 | 65.35 | -225.37 | -3.90 | 1.01 | 518.29 | 257.81 |
| brown_coral | -51.14 | 54.84 | -204.31 | -0.07 | 1.00 | 822.80 | 699.49 |
| green_coral | -48.69 | 62.40 | -204.17 | 0.09 | 1.02 | 395.60 | 190.23 |
| grey_coral | -48.79 | 56.45 | -195.92 | 0.72 | 1.00 | 672.06 | 593.83 |
| mix_coral | -57.02 | 103.57 | -272.54 | 2.94 | 1.01 | 634.81 | 303.54 |
| orange_coral | -77.10 | 181.75 | -776.14 | 2.78 | 1.01 | 221.27 | 57.45 |
| pink_coral | -51.65 | 71.58 | -218.99 | 1.59 | 1.01 | 637.24 | 305.25 |
| purple_coral | -46.27 | 54.73 | -197.44 | 1.24 | 1.01 | 814.37 | 632.61 |
| red_coral | 0.50 | 1.98 | -3.37 | 4.77 | 1.00 | 1300.09 | 1354.10 |
| transparent_coral | 1.56 | 1.61 | -0.96 | 5.47 | 1.00 | 1279.15 | 1179.48 |
| white_coral | 3.30 | 1.60 | 0.82 | 7.17 | 1.00 | 1351.88 | 1182.28 |
| yellow_coral | -45.50 | 59.00 | -186.39 | 1.67 | 1.01 | 692.21 | 487.25 |

(b) Bayesian posterior distribution in differences. Estimate represents the estimated average difference. Lower and upper CI represent the estimated 95% credible intervals.

| colour | mean_diff | lower_ci | upper_ci |
| --- | --- | --- | --- |
| black | 0.03 | -0.14 | 0.11 |
| blue | 0.43 | 0.37 | 0.50 |
| brown | 0.04 | 0.02 | 0.07 |
| green | 0.04 | 0.02 | 0.07 |
| grey | 0.02 | 0.00 | 0.04 |
| mix | 0.00 | -0.01 | 0.02 |
| orange | 0.00 | -0.01 | 0.02 |
| pink | 0.01 | 0.00 | 0.03 |
| purple | 0.02 | 0.00 | 0.04 |
| red | 0.01 | -0.17 | 0.10 |
| transparent | -0.11 | -0.36 | 0.09 |
| white | -0.51 | -0.74 | -0.26 |
| yellow | 0.01 | -0.01 | 0.03 |

Table S26: Summary data for the Bayesian regression model (a) and respective posterior distributions (b) analysing differences and associated uncertainty of microplastic colour between seafloor sediment and sea cucumber. Number of observations 77. CI = credible interval. Rhat = scale reduction factor. ESS = effective sample size.

(a) Bayesian model summary.

| Effects | Estimate | Est.Error | l-95% CI | u-95% CI | Rhat | Bulk_ESS | Tail_ESS |
| --- | --- | --- | --- | --- | --- | --- | --- |
| blue_Intercept | 0.29 | 0.63 | -0.94 | 1.57 | 1.00 | 2172.83 | 2138.83 |
| brown_Intercept | -0.91 | 0.92 | -2.88 | 0.75 | 1.00 | 2607.72 | 2336.48 |
| green_Intercept | -9.67 | 7.71 | -30.80 | -1.37 | 1.00 | 1178.00 | 766.00 |
| mix_Intercept | -9.73 | 9.52 | -33.49 | -1.19 | 1.00 | 1460.84 | 844.46 |
| pink_Intercept | -9.37 | 7.90 | -29.54 | -1.23 | 1.00 | 1108.75 | 673.06 |
| red_Intercept | -0.49 | 0.80 | -2.16 | 1.00 | 1.00 | 2140.55 | 2554.99 |
| transparent_Intercept | -0.16 | 0.72 | -1.64 | 1.25 | 1.00 | 2159.54 | 2147.03 |
| white_Intercept | 0.11 | 0.67 | -1.18 | 1.46 | 1.00 | 2453.70 | 2836.31 |
| yellow_Intercept | -10.68 | 12.56 | -37.88 | -1.29 | 1.00 | 591.78 | 239.37 |
| blue_sediment | 1.37 | 0.86 | -0.32 | 3.04 | 1.00 | 1524.90 | 2004.81 |
| brown_sediment | -4.63 | 5.96 | -20.60 | 0.94 | 1.01 | 617.20 | 288.01 |
| green_sediment | 9.97 | 7.73 | 1.44 | 30.94 | 1.00 | 1136.68 | 756.28 |
| mix_sediment | 8.29 | 9.56 | -0.96 | 32.38 | 1.00 | 1428.53 | 860.18 |
| pink_sediment | 8.67 | 7.96 | 0.04 | 28.65 | 1.00 | 1064.91 | 709.72 |
| red_sediment | 0.76 | 1.08 | -1.30 | 2.89 | 1.00 | 1875.93 | 2591.80 |
| transparent_sediment | 1.26 | 0.95 | -0.56 | 3.14 | 1.00 | 1529.08 | 2402.82 |
| white_sediment | -1.70 | 1.38 | -4.57 | 0.70 | 1.01 | 1300.46 | 1454.98 |
| yellow_sediment | 10.44 | 12.61 | 0.70 | 38.03 | 1.00 | 591.88 | 240.45 |

(b) Bayesian posterior distribution in differences. Estimate represents the estimated average difference. Lower and upper CI represent the estimated 95% credible intervals.

| colour | mean_diff | lower_ci | upper_ci |
| --- | --- | --- | --- |
| black | -0.11 | -0.28 | 0.04 |
| blue | 0.13 | -0.09 | 0.33 |
| brown | -0.08 | -0.21 | -0.01 |
| green | 0.10 | 0.03 | 0.19 |
| mix | 0.02 | -0.03 | 0.08 |
| pink | 0.04 | -0.01 | 0.11 |
| red | -0.02 | -0.19 | 0.11 |
| transparent | 0.05 | -0.14 | 0.22 |
| white | -0.18 | -0.36 | -0.04 |
| yellow | 0.06 | 0.00 | 0.14 |

Table S27: Summary data for the Bayesian regression model (a) and respective posterior distributions (b) analysing differences and associated uncertainty of microplastic polymer between mid-column water and fish. Number of observations 291. CI = credible interval. Rhat = scale reduction factor. ESS = effective sample size.

(a) Bayesian model summary.

| Effects | Estimate | Est.Error | l-95% CI | u-95% CI | Rhat | Bulk_ESS | Tail_ESS |
| --- | --- | --- | --- | --- | --- | --- | --- |
| EPDM_Intercept | -1.25 | 0.43 | -2.13 | -0.43 | 1.00 | 3421.09 | 3116.85 |
| nylon_Intercept | -0.89 | 0.39 | -1.69 | -0.17 | 1.00 | 4214.14 | 3212.83 |
| other_Intercept | 0.06 | 0.28 | -0.50 | 0.62 | 1.00 | 4764.29 | 3030.57 |
| PE_Intercept | -1.00 | 0.39 | -1.83 | -0.27 | 1.00 | 4184.59 | 2600.52 |
| PET_Intercept | 1.14 | 0.24 | 0.69 | 1.62 | 1.00 | 5030.13 | 3028.44 |
| PSU_Intercept | -3.33 | 1.05 | -5.80 | -1.67 | 1.00 | 4003.35 | 2220.91 |
| PP_Intercept | 1.23 | 0.23 | 0.79 | 1.70 | 1.00 | 4731.34 | 3019.72 |
| PPPE_Intercept | -1.67 | 0.51 | -2.75 | -0.72 | 1.00 | 4357.41 | 2396.38 |
| PS_Intercept | -1.31 | 0.43 | -2.21 | -0.50 | 1.00 | 4917.65 | 3327.51 |
| PVC_Intercept | -0.69 | 0.36 | -1.43 | 0.00 | 1.00 | 4289.59 | 3582.24 |
| EPDM_fish | -9.83 | 28.66 | -83.73 | 22.41 | 1.01 | 681.80 | 463.26 |
| nylon_fish | -9.50 | 22.50 | -70.84 | 20.45 | 1.00 | 803.82 | 651.56 |
| other_fish | 16.88 | 8.49 | 2.80 | 35.95 | 1.01 | 596.02 | 834.49 |
| PE_fish | -12.84 | 38.69 | -83.66 | 20.51 | 1.01 | 535.01 | 464.42 |
| PET_fish | 18.13 | 8.48 | 4.22 | 37.11 | 1.01 | 591.77 | 845.52 |
| PSU_fish | -6.52 | 29.74 | -91.65 | 28.18 | 1.01 | 591.65 | 373.48 |
| PP_fish | 14.18 | 8.56 | 0.32 | 32.99 | 1.01 | 599.53 | 964.36 |
| PPPE_fish | 18.11 | 8.56 | 4.11 | 37.20 | 1.01 | 590.64 | 853.05 |
| PS_fish | 16.73 | 8.56 | 2.86 | 35.92 | 1.01 | 597.98 | 851.39 |
| PVC_fish | -11.78 | 26.20 | -86.12 | 20.37 | 1.00 | 874.16 | 528.54 |

(b) Bayesian posterior distribution in differences. Estimate represents the estimated average difference. Lower and upper CI represent the estimated 95% credible intervals.

| polymer_type | mean_diff | lower_ci | upper_ci |
| --- | --- | --- | --- |
| acrylic | 0.09 | 0.06 | 0.13 |
| EPDM | 0.03 | 0.01 | 0.05 |
| nylon | 0.04 | 0.02 | 0.07 |
| other | 0.01 | -0.11 | 0.09 |
| PE | 0.03 | 0.01 | 0.06 |
| PET | -0.50 | -0.63 | -0.34 |
| PSU | 0.00 | -0.01 | 0.02 |
| PP | 0.29 | 0.20 | 0.36 |
| PP:PE | -0.04 | -0.14 | 0.02 |
| PS | 0.00 | -0.08 | 0.04 |
| PVC | 0.05 | 0.02 | 0.08 |

Table S28: Summary data for the Bayesian regression model (a) and respective posterior distributions (b) analysing differences and associated uncertainty of microplastic polymer between mid-column water and sea squirt. Number of observations 272. CI = credible interval. Rhat = scale reduction factor. ESS = effective sample size.

(a) Bayesian model summary.

| Effects | Estimate | Est.Error | l-95% CI | u-95% CI | Rhat | Bulk_ESS | Tail_ESS |
| --- | --- | --- | --- | --- | --- | --- | --- |
| EPDM_Intercept | -1.16 | 0.44 | -2.06 | -0.33 | 1.00 | 2851.97 | 2380.78 |
| nylon_Intercept | -0.78 | 0.38 | -1.54 | -0.08 | 1.00 | 2461.89 | 2806.88 |
| other_Intercept | 0.18 | 0.29 | -0.38 | 0.74 | 1.00 | 1687.35 | 2478.28 |
| PE_Intercept | -0.89 | 0.39 | -1.68 | -0.15 | 1.00 | 2455.51 | 2705.44 |
| PET_Intercept | 1.24 | 0.23 | 0.80 | 1.70 | 1.00 | 1163.72 | 1833.79 |
| PSU_Intercept | -3.12 | 1.05 | -5.58 | -1.48 | 1.00 | 1335.41 | 958.91 |
| PP_Intercept | 1.33 | 0.23 | 0.88 | 1.81 | 1.00 | 1119.64 | 1462.95 |
| PPPE_Intercept | -1.47 | 0.49 | -2.50 | -0.57 | 1.00 | 3030.42 | 2592.96 |
| PS_Intercept | -1.15 | 0.42 | -2.02 | -0.35 | 1.00 | 1565.83 | 1790.63 |
| PVC_Intercept | -0.62 | 0.35 | -1.34 | 0.05 | 1.00 | 1434.82 | 2080.96 |
| EPDM_ssquirt | -48.88 | 52.60 | -200.43 | 0.19 | 1.02 | 434.14 | 281.13 |
| nylon_ssquirt | -50.32 | 55.59 | -211.15 | -0.07 | 1.01 | 568.54 | 338.34 |
| other_ssquirt | -55.82 | 61.85 | -223.33 | -1.52 | 1.01 | 571.02 | 276.43 |
| PE_ssquirt | -52.53 | 66.15 | -236.14 | 0.04 | 1.00 | 641.66 | 286.58 |
| PET_ssquirt | 1.52 | 1.41 | -0.65 | 4.88 | 1.01 | 826.92 | 721.34 |
| PSU_ssquirt | 3.27 | 2.17 | -0.71 | 7.82 | 1.01 | 900.67 | 1033.67 |
| PP_ssquirt | -0.16 | 1.59 | -2.91 | 3.41 | 1.00 | 851.99 | 790.17 |
| PPPE_ssquirt | -50.59 | 61.68 | -206.62 | 0.62 | 1.01 | 685.41 | 517.22 |
| PS_ssquirt | 1.38 | 1.87 | -2.35 | 5.34 | 1.00 | 935.41 | 832.31 |
| PVC_ssquirt | 0.79 | 1.88 | -2.80 | 4.59 | 1.00 | 886.66 | 814.73 |

(b) Bayesian posterior distribution in differences. Estimate represents the estimated average difference. Lower and upper CI represent the estimated 95% credible intervals.

| polymer_type | mean_diff | lower_ci | upper_ci |
| --- | --- | --- | --- |
| acrylic | 0.02 | -0.15 | 0.10 |
| EPDM | 0.03 | 0.01 | 0.05 |
| nylon | 0.04 | 0.01 | 0.07 |
| other | 0.10 | 0.07 | 0.14 |
| PE | 0.03 | 0.01 | 0.06 |
| PET | -0.28 | -0.53 | -0.02 |
| PSU | -0.07 | -0.24 | 0.00 |
| PP | 0.17 | -0.05 | 0.32 |
| PP:PE | 0.02 | 0.00 | 0.04 |
| PS | -0.04 | -0.22 | 0.03 |
| PVC | -0.03 | -0.20 | 0.06 |

Table S29: Summary data for the Bayesian regression model (a) and respective posterior distributions (b) analysing differences and associated uncertainty of microplastic polymer between mid-column water and sponge. Number of observations 271. CI = credible interval. Rhat = scale reduction factor. ESS = effective sample size.

(a) Bayesian model summary.

| Effects | Estimate | Est.Error | l-95% CI | u-95% CI | Rhat | Bulk_ESS | Tail_ESS |
| --- | --- | --- | --- | --- | --- | --- | --- |
| EPDM_Intercept | -1.16 | 0.43 | -2.05 | -0.36 | 1.00 | 2087.54 | 2543.70 |
| nylon_Intercept | -0.82 | 0.37 | -1.54 | -0.08 | 1.00 | 1334.21 | 1717.87 |
| other_Intercept | 0.16 | 0.28 | -0.39 | 0.71 | 1.01 | 1107.21 | 1797.97 |
| PE_Intercept | -0.91 | 0.39 | -1.69 | -0.16 | 1.00 | 2275.99 | 2561.96 |
| PET_Intercept | 1.23 | 0.24 | 0.77 | 1.69 | 1.01 | 973.84 | 1361.37 |
| PSU_Intercept | -3.12 | 1.00 | -5.49 | -1.59 | 1.00 | 1172.80 | 1079.15 |
| PP_Intercept | 1.32 | 0.23 | 0.87 | 1.78 | 1.01 | 959.82 | 1447.86 |
| PPPE_Intercept | -1.51 | 0.51 | -2.60 | -0.58 | 1.00 | 2607.75 | 2652.80 |
| PS_Intercept | -1.16 | 0.43 | -2.05 | -0.38 | 1.00 | 2263.40 | 2468.54 |
| PVC_Intercept | -0.64 | 0.35 | -1.34 | 0.00 | 1.01 | 1185.70 | 1506.07 |
| EPDM_sponge | -48.93 | 52.91 | -203.26 | 0.11 | 1.01 | 750.65 | 462.43 |
| nylon_sponge | 1.97 | 1.57 | -0.83 | 5.45 | 1.01 | 737.01 | 829.17 |
| other_sponge | -0.01 | 1.84 | -3.65 | 3.70 | 1.00 | 832.95 | 1048.09 |
| PE_sponge | -57.87 | 72.46 | -251.73 | -0.47 | 1.01 | 606.52 | 401.85 |
| PET_sponge | -0.10 | 1.56 | -2.80 | 3.26 | 1.01 | 741.64 | 957.32 |
| PSU_sponge | 3.30 | 2.11 | -0.94 | 7.43 | 1.01 | 801.77 | 1239.99 |
| PP_sponge | 0.91 | 1.39 | -1.34 | 4.12 | 1.01 | 689.15 | 800.10 |
| PPPE_sponge | -53.22 | 62.05 | -226.99 | 0.71 | 1.03 | 151.77 | 57.01 |
| PS_sponge | -46.59 | 48.87 | -185.60 | 0.26 | 1.01 | 492.02 | 382.42 |
| PVC_sponge | 0.82 | 1.84 | -2.75 | 4.51 | 1.01 | 825.28 | 1055.48 |

(b) Bayesian posterior distribution in differences. Estimate represents the estimated average difference. Lower and upper CI represent the estimated 95% credible intervals.

| polymer_type | mean_diff | lower_ci | upper_ci |
| --- | --- | --- | --- |
| acrylic | 0.02 | -0.16 | 0.10 |
| EPDM | 0.03 | 0.01 | 0.05 |
| nylon | -0.12 | -0.34 | 0.02 |
| other | 0.02 | -0.17 | 0.12 |
| PE | 0.03 | 0.01 | 0.06 |
| PET | 0.14 | -0.10 | 0.29 |
| PSU | -0.07 | -0.26 | 0.00 |
| PP | -0.07 | -0.33 | 0.18 |
| PP:PE | 0.02 | 0.00 | 0.04 |
| PS | 0.03 | 0.01 | 0.05 |
| PVC | -0.03 | -0.22 | 0.05 |

Table S30: Summary data for the Bayesian regression model (a) and respective posterior distributions (b) analysing differences and associated uncertainty of microplastic polymer between mid-column water and coral. Number of observations 272. CI = credible interval. Rhat = scale reduction factor. ESS = effective sample size.

(a) Bayesian model summary.

| Effects | Estimate | Est.Error | l-95% CI | u-95% CI | Rhat | Bulk_ESS | Tail_ESS |
| --- | --- | --- | --- | --- | --- | --- | --- |
| EPDM_Intercept | -1.30 | 0.43 | -2.20 | -0.49 | 1.00 | 5144.84 | 2565.50 |
| nylon_Intercept | -0.90 | 0.38 | -1.70 | -0.18 | 1.00 | 4171.39 | 2755.36 |
| other_Intercept | 0.07 | 0.28 | -0.50 | 0.60 | 1.00 | 5121.25 | 3291.05 |
| PE_Intercept | -0.99 | 0.38 | -1.79 | -0.26 | 1.00 | 4224.91 | 3125.38 |
| PET_Intercept | 1.14 | 0.23 | 0.71 | 1.61 | 1.00 | 4459.68 | 3412.25 |
| PSU_Intercept | -3.39 | 1.05 | -5.79 | -1.71 | 1.00 | 2597.67 | 1957.90 |
| PP_Intercept | 1.24 | 0.23 | 0.79 | 1.69 | 1.00 | 4264.65 | 3511.11 |
| PPPE_Intercept | -1.61 | 0.50 | -2.68 | -0.71 | 1.00 | 4017.17 | 2815.41 |
| PS_Intercept | -1.26 | 0.43 | -2.13 | -0.47 | 1.00 | 4181.40 | 2698.61 |
| PVC_Intercept | -0.71 | 0.35 | -1.42 | -0.04 | 1.00 | 4097.45 | 2795.43 |
| EPDM_coral | 39.31 | 20.23 | 6.75 | 83.37 | 1.00 | 394.27 | 633.62 |
| nylon_coral | -24.63 | 64.32 | -190.05 | 50.07 | 1.01 | 519.86 | 323.21 |
| other_coral | 38.93 | 20.19 | 6.69 | 82.43 | 1.00 | 391.45 | 645.31 |
| PE_coral | -19.40 | 52.03 | -146.33 | 51.71 | 1.01 | 803.53 | 680.62 |
| PET_coral | 37.86 | 20.15 | 5.71 | 81.44 | 1.00 | 391.34 | 605.22 |
| PSU_coral | 43.85 | 20.33 | 11.20 | 87.87 | 1.00 | 396.15 | 653.46 |
| PP_coral | 37.77 | 20.20 | 5.39 | 81.26 | 1.00 | 392.25 | 616.00 |
| PPPE_coral | -16.36 | 58.25 | -173.65 | 54.29 | 1.00 | 729.75 | 514.49 |
| PS_coral | -16.33 | 50.93 | -152.09 | 50.64 | 1.00 | 707.24 | 609.55 |
| PVC_coral | -24.15 | 57.05 | -171.82 | 48.37 | 1.00 | 699.34 | 521.60 |

(b) Bayesian posterior distribution in differences. Estimate represents the estimated average difference. Lower and upper CI represent the estimated 95% credible intervals.

| polymer_type | mean_diff | lower_ci | upper_ci |
| --- | --- | --- | --- |
| acrylic | 0.09 | 0.06 | 0.13 |
| EPDM | -0.05 | -0.23 | 0.03 |
| nylon | 0.04 | 0.02 | 0.07 |
| other | -0.04 | -0.27 | 0.09 |
| PE | 0.04 | 0.02 | 0.06 |
| PET | 0.15 | -0.08 | 0.29 |
| PSU | -0.49 | -0.74 | -0.25 |
| PP | 0.18 | -0.05 | 0.32 |
| PP:PE | 0.02 | 0.01 | 0.04 |
| PS | 0.03 | 0.01 | 0.05 |
| PVC | 0.05 | 0.02 | 0.08 |

Table S31: Summary data for the Bayesian regression model (a) and respective posterior distributions (b) analysing differences and associated uncertainty of microplastic polymer between seafloor sediment and sea cucumber. Number of observations 77. CI = credible interval. Rhat = scale reduction factor. ESS = effective sample size.

(a) Bayesian model summary.

| Effects | Estimate | Est.Error | l-95% CI | u-95% CI | Rhat | Bulk_ESS | Tail_ESS |
| --- | --- | --- | --- | --- | --- | --- | --- |
| EPDM_Intercept | -10.39 | 8.75 | -34.94 | -1.86 | 1.00 | 1319.57 | 893.76 |
| nylon_Intercept | -2.63 | 1.25 | -5.51 | -0.66 | 1.00 | 2320.56 | 1841.16 |
| other_Intercept | -1.67 | 0.85 | -3.54 | -0.16 | 1.00 | 2762.05 | 2304.32 |
| PE_Intercept | -10.69 | 9.83 | -38.23 | -2.07 | 1.01 | 577.01 | 313.26 |
| PET_Intercept | -0.24 | 0.52 | -1.25 | 0.79 | 1.00 | 3836.99 | 3194.13 |
| PP_Intercept | -2.56 | 1.23 | -5.37 | -0.58 | 1.00 | 2384.05 | 1954.28 |
| PPPE_Intercept | -2.46 | 1.20 | -5.29 | -0.60 | 1.00 | 3245.69 | 2127.47 |
| PS_Intercept | -1.12 | 0.73 | -2.70 | 0.22 | 1.00 | 3187.33 | 1960.76 |
| PVC_Intercept | -2.54 | 1.27 | -5.44 | -0.57 | 1.00 | 2920.00 | 1873.01 |
| EPDM_sediment | 9.27 | 8.84 | 0.04 | 33.96 | 1.00 | 1302.99 | 916.43 |
| nylon_sediment | 3.60 | 1.45 | 1.07 | 6.83 | 1.00 | 1885.44 | 1808.73 |
| other_sediment | 3.19 | 1.09 | 1.24 | 5.53 | 1.00 | 1715.29 | 1791.10 |
| PE_sediment | 10.80 | 9.85 | 1.77 | 38.44 | 1.01 | 568.25 | 310.36 |
| PET_sediment | 2.29 | 0.81 | 0.73 | 3.98 | 1.00 | 1601.60 | 1716.07 |
| PP_sediment | 2.62 | 1.49 | 0.01 | 5.97 | 1.00 | 1932.58 | 2110.36 |
| PPPE_sediment | -2.66 | 5.34 | -17.88 | 3.32 | 1.00 | 833.39 | 432.61 |
| PS_sediment | -3.69 | 4.04 | -14.50 | 1.28 | 1.00 | 1192.02 | 857.63 |
| PVC_sediment | 2.15 | 1.59 | -0.68 | 5.44 | 1.00 | 2092.15 | 2371.48 |

(b) Bayesian posterior distribution in differences. Estimate represents the estimated average difference. Lower and upper CI represent the estimated 95% credible intervals.

| polymer_type | mean_diff | lower_ci | upper_ci |
| --- | --- | --- | --- |
| acrylic | -0.29 | -0.49 | -0.10 |
| EPDM | 0.02 | -0.03 | 0.08 |
| nylon | 0.10 | -0.03 | 0.21 |
| other | 0.15 | -0.02 | 0.30 |
| PE | 0.06 | 0.00 | 0.14 |
| PET | 0.11 | -0.12 | 0.31 |
| PP | 0.02 | -0.10 | 0.11 |
| PP:PE | -0.04 | -0.14 | 0.01 |
| PS | -0.12 | -0.28 | -0.02 |
| PVC | 0.00 | -0.12 | 0.08 |


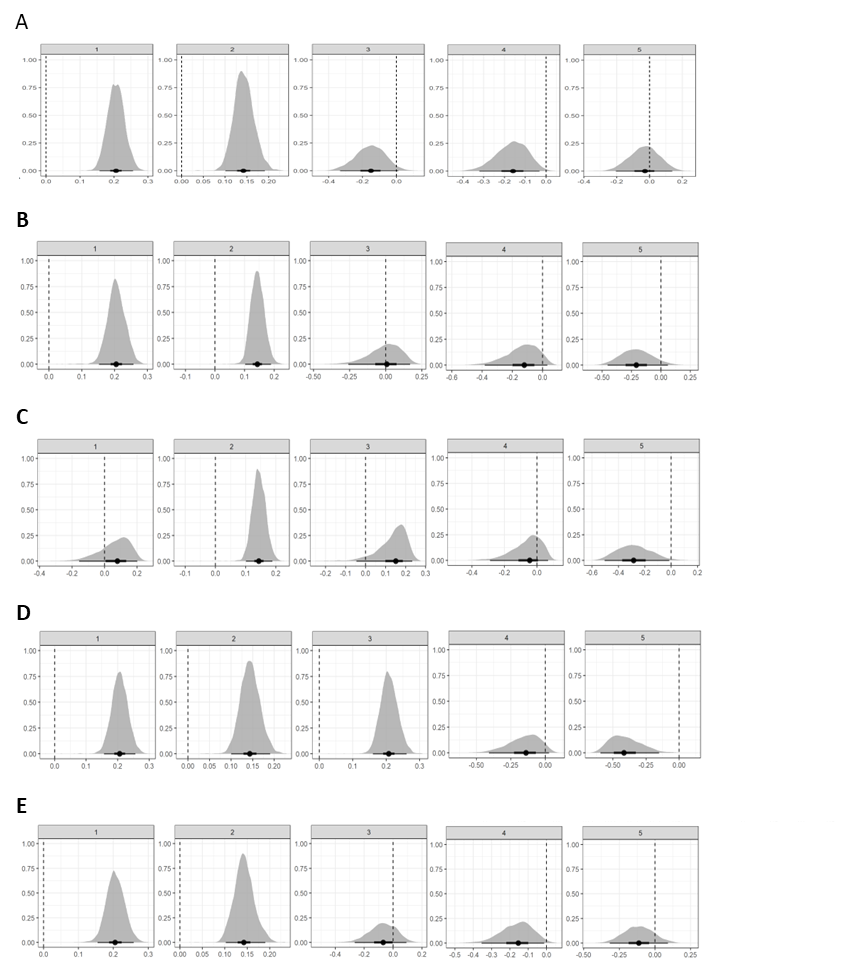


Figure S9: Bayesian posterior distribution as the difference in microplastic size classes between mid-column water and fish (A), sea squirt (B), sponge (C) and coral (D), and between seafloor sediment and sea cucumber (E). Black point represents the mean difference. Horizontal error bars are Bayesian 50% (thick) and 90% (thin) credible intervals. Absolute differences between both treatments were expected to be centred around 0, which is indicated by the dashed black line. Size classes included class 1: ≥ 5 mm, class 2: < 5 mm and ≥ 2.5, class 3: < 2.5 mm and ≥ 1, class 4: < 1mm and ≤ 500 µm, and class 5: < 500 µm.


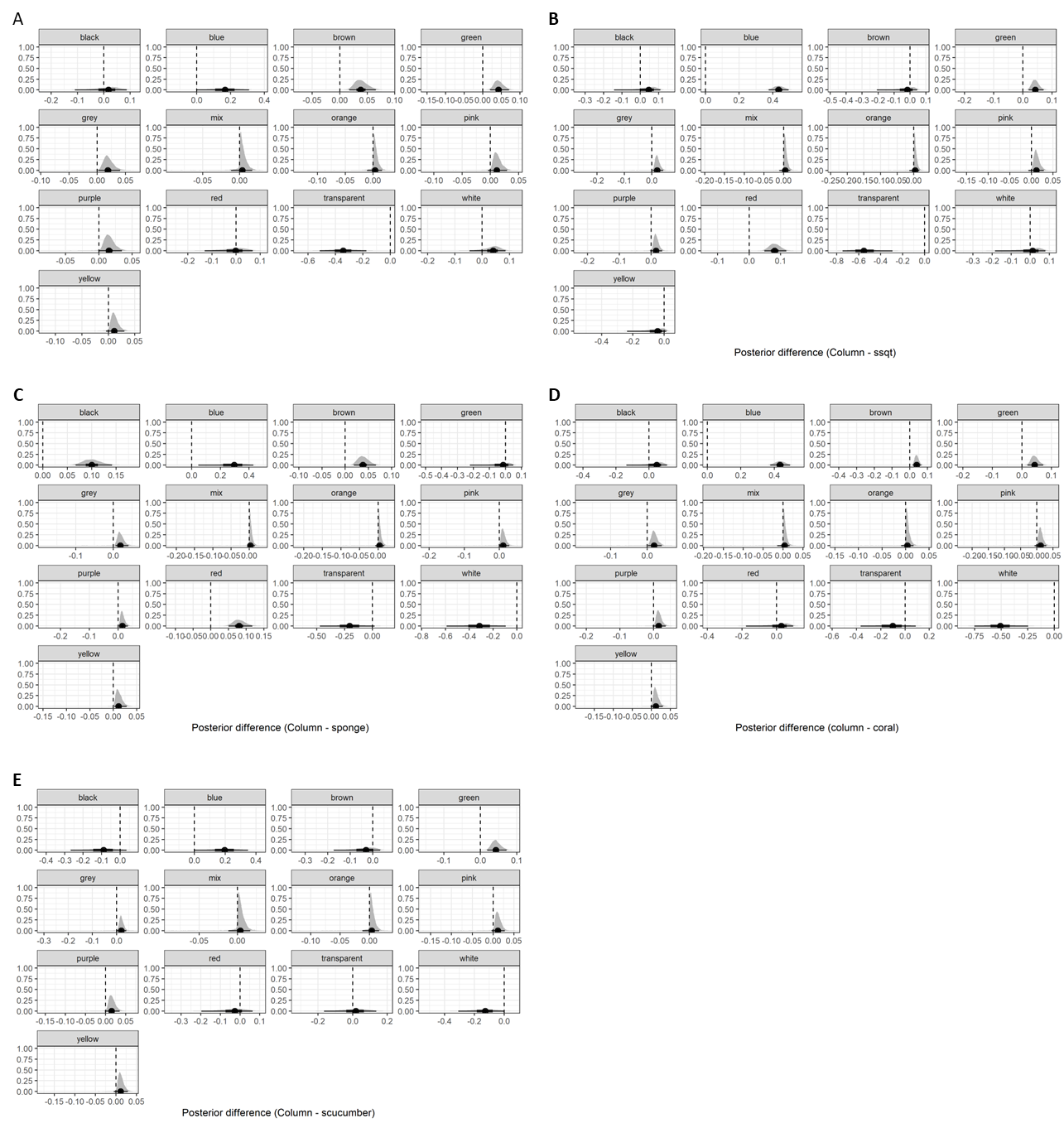


Figure S10: Bayesian posterior distribution as the difference in microplastic colours between mid-column water and fish (A), sea squirt (B), sponge (C) and coral (D), and between seafloor sediment and sea cucumber (E). Black point represents the mean difference. Horizontal error bars are Bayesian 50% (thick) and 90% (thin) credible intervals. Absolute differences between both treatments were expected to be centred around 0, which is indicated by the dashed black line. Colours included black, blue, brown, green, grey, mix (i.e., mixture of more than one colour), orange, pink, purple, red, transparent, white, and yellow.


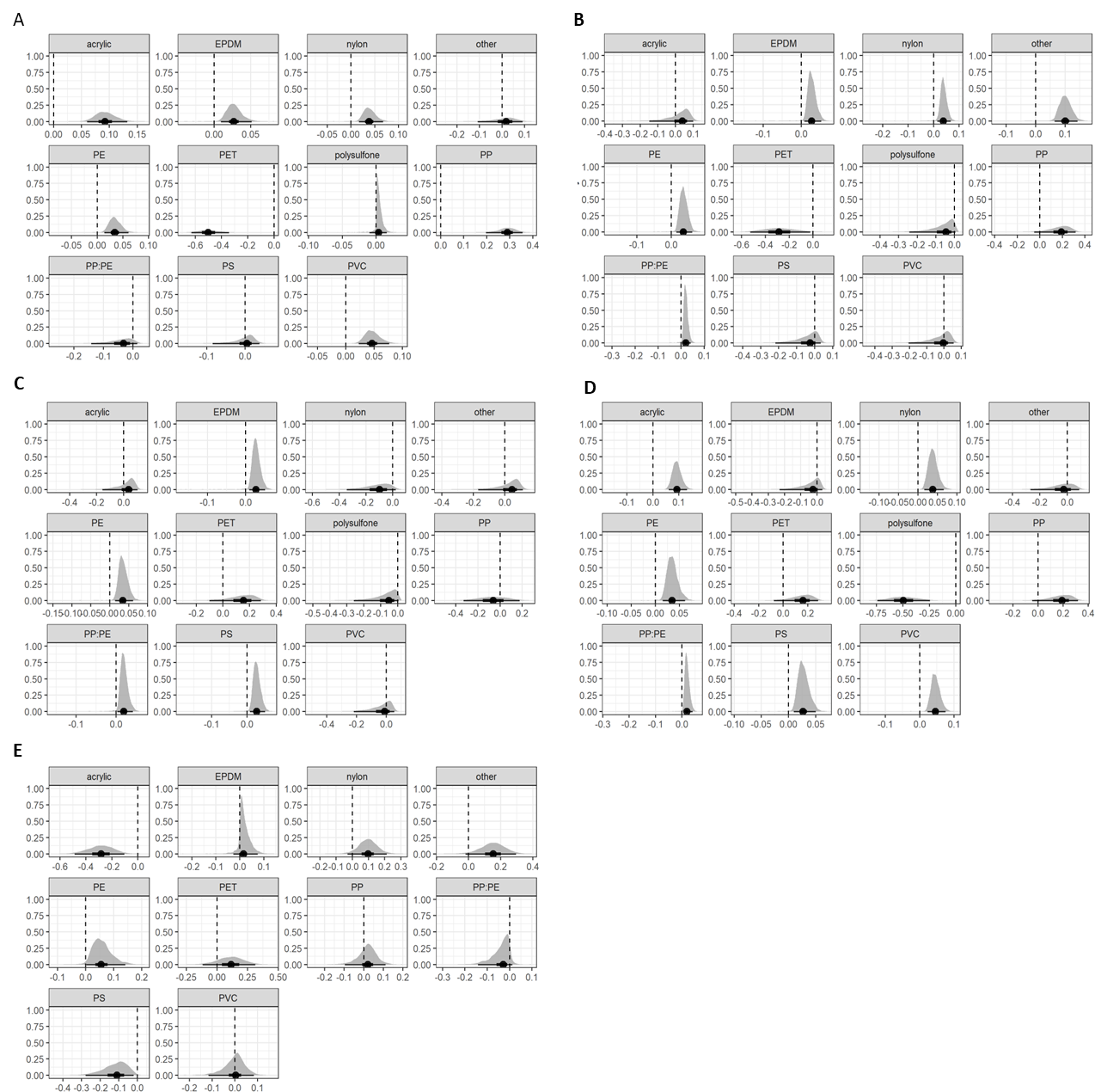


Figure S11: Bayesian posterior distribution as the difference in microplastic polymers between mid-column water and fish (A), sea squirt (B), sponge (C) and coral (D), and between seafloor sediment and sea cucumber (E). Black point represents the mean difference. Horizontal error bars are Bayesian 50% (thick) and 90% (thin) credible intervals. Absolute differences between both treatments were expected to be centred around 0, which is indicated by the dashed black line. Polymer types included acrylic, ethylene propylene diene (EPDM), nylon, other (i.e., all polymer types with less than 10% of representativeness in the data set), polyethylene (PE), mix (i.e., mixture of more than one colour), polyethyleneterephthalate and polyester (PET), polysulfone (PSU), polypropylene (PP), polypropylene:polyethylene blend (PP:PE), polystyrene (PS), and polyvinylchloride (PVC).


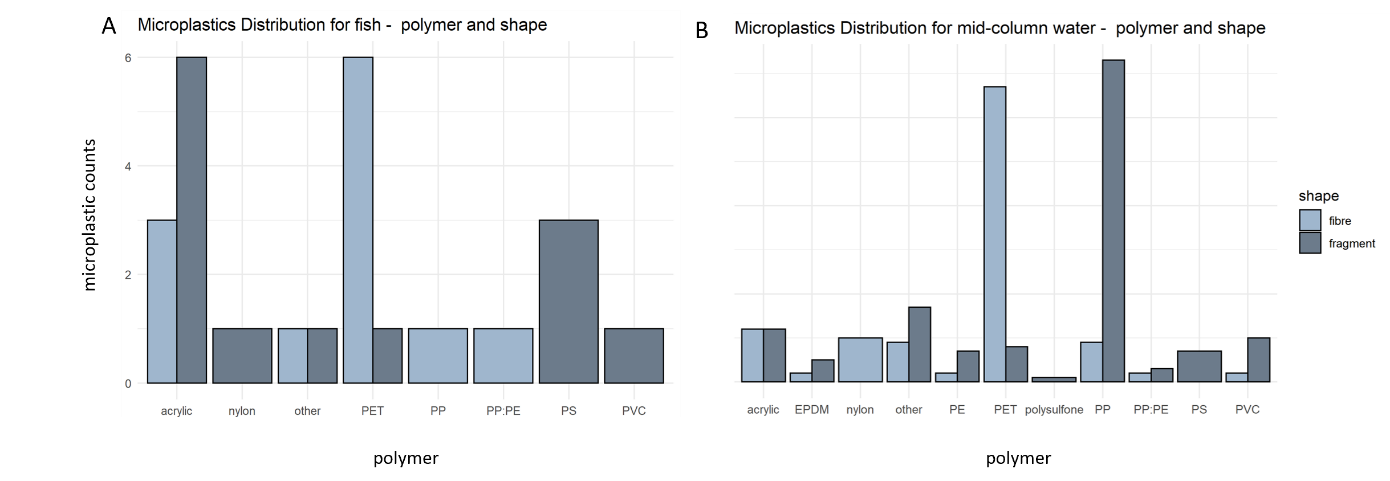


Figure S12: Microplastic distribution (counts) per combination of polymer and shape for (A) fish and (B) mid-column water.

Table S32: Summary of microplastic (mp) exposure and bioavailability field studies conducted in the Great Barrier Reef World Heritage Area (GBRWHA). (*) Studies that assessed microplastic contamination in more than one matrix type.

| **Area of study** | **Matrix** | **Concentration of microplastics found** | **Reference** |
| --- | --- | --- | --- |
| Northern GBRWHA | surface water | 956.41 to 13518.32 mp km^-2^ | (Reisser et al. 2013) |
| Central GBRWHA | surface water | 0.04 to 0.48 mp m^−3^ | (Jensen et al. 2019)* |
| Central GBRWHA (SS Yongala shipwreck) | surface water | 0.13 ± 0.17 (average ± standard deviation) mp m^−3^ and up to 0.92 | (Miller et al. 2022b) |
| Central GBRWHA (Orpheus Island) | subsurface water | 2 x 10^-7^ mp m^−3^ | (Hall et al. 2015) |
| Central GBRWHA (Davies and Backnumbers reefs) | mid-column water | 4.66 ± 4.24 (average ± standard error) mp m^−3^ | (Miller et al. 2022a)* |
| Central GBRWHA (Davies and Backnumbers reefs) | sediment | 3.22 ± 3.41 (average ± standard error) mp kg^−1^ | (Miller et al. 2022a)* |
| Northern GBRWHA (Cairns region) | turtle (*Chelonia mydas*) | 3.5 mp individual ^−1^ | (Caron et al. 2018) |
| Northern, central and southern GBRWHA (Lizard Island, Orpheus Island, Heron Island and One Tree Island) | fish (*Plectropomus spp*.) | 5.8 ± 0.8 (average ± standard error) mp individual^−1^ | (Kroon et al. 2018b) |
| Central GBRWHA | fish (*Pomacentrus amboinensis*) | 0 to 131 mp individual^−1^ | (Jensen et al. 2019)* |
| Northern GBRWHA  (Lizard Island) | fish (*P. amboinensis*) | Average of 2.25 mp individual^−1^ | (Santana et al. 2021) |
| Commercial fishing areas in Queensland | fish (common coral trout, paddletail, sea mullet, and other species from Family Mullidae) | 1.58 ± 0.23 (average ± standard deviation) mp individual^−1^ | (Wootton et al. 2021) |
| Commercial fishing areas in the GBRWHA | fish (*Plectropomus leopardus*), scallop (*Ylistrum balloti*), and prawn (*Melicertus latisulcatus*) | Only two coral trouts contained mps (two fibres each) | (Dawson et al. 2022) |
| Central GBRWHA (Pallarenda beach) | fish (Australian sharpnose, *Rhizoprionodon taylori*) | 14 ± 4 (average ± standard error) mp individual^−1^ | (Schlawinsky et al. 2022) |
| Central GBRWHA (Davies and Backnumbers reefs) | fish (moon wrasse, *Thalassoma lunare*), copepod (no species id), and benthic crustaceans (no species id) | 0.16 ± 0.22, 3.72 ± 3.14 and 3.56 ± 5.12 (average ± standard error) mp g^−1^ per respective taxon | (Miller et al. 2022a)* |

References

Bakir A, Doran D, Silburn B, Russell J, Archer-Rand S, Barry J, Maes T, Limpenny C, Mason C, Barber J, Nicolaus EEM (2023): A spatial and temporal assessment of microplastics in seafloor sediments: A case study for the UK. Frontiers in Marine Science 9

Caron AGM, Thomas CR, Berry KLE, Motti CA, Ariel E, Brodie JE (2018): Ingestion of microplastic debris by green sea turtles (Chelonia mydas) in the Great Barrier Reef: Validation of a sequential extraction protocol. Mar Pollut Bull 127, 743-751

Chubarenko I, Bagaev A, Zobkov M, Esiukova E (2016): On some physical and dynamical properties of microplastic particles in marine environment. Mar Pollut Bull 108, 105-12

Dawson AL, Li JYQ, Kroon FJ (2022): Plastics for dinner: Store-bought seafood, but not wild-caught from the Great Barrier Reef, as a source of microplastics to human consumers. Environmental Advances 8

Graham ER, Thompson JT (2009): Deposit- and suspension-feeding sea cucumbers (Echinodermata) ingest plastic fragments. Journal of Experimental Marine Biology and Ecology 368, 22-29

Hall NM, Berry KLE, Rintoul L, Hoogenboom MO (2015): Microplastic ingestion by scleractinian corals. Marine Biology 162, 725-732

Hidalgo-Ruz V, Gutow L, Thompson RC, Thiel M (2012): Microplastics in the marine environment: a review of the methods used for identification and quantification. Environ Sci Technol 46, 3060-75

Hrycik JM, Chassé J, Ruddick BR, Taggart CT (2013): Dispersal kernel estimation: A comparison of empirical and modelled particle dispersion in a coastal marine system. Estuarine, Coastal and Shelf Science 133, 11-22

Jensen LH, Motti CA, Garm AL, Tonin H, Kroon FJ (2019): Sources, distribution and fate of microfibres on the Great Barrier Reef, Australia. Sci Rep 9, 9021

Kroon F, Motti C, Talbot S, Sobral P, Puotinen M (2018a): A workflow for improving estimates of microplastic contamination in marine waters: A case study from North-Western Australia. Environ Pollut 238, 26-38

Kroon FJ (2015): The efficacy of clove oil for anaesthesia of eight species of Australian tropical freshwater teleosts. Limnology and Oceanography: Methods 13, 463-475

Kroon FJ, Motti CE, Jensen LH, Berry KLE (2018b): Classification of marine microdebris: A review and case study on fish from the Great Barrier Reef, Australia. Sci Rep 8, 16422

Liu K, Courtene-Jones W, Wang X, Song Z, Wei N, Li D (2020): Elucidating the vertical transport of microplastics in the water column: A review of sampling methodologies and distributions. Water Res 186, 116403

Miller ME, Motti CA, Hamann M, Kroon FJ (2022a): Assessment of microplastic bioconcentration, bioaccumulation and biomagnification in a simple coral reef food web. Sci Total Environ 858, 159615

Miller ME, Santana MFM, Carsique M, Motti CA, Hamann M, Kroon FJ (2022b): Temporal patterns of plastic contamination in surface waters at the SS Yongala shipwreck, Great Barrier Reef, Australia. Environ Pollut 307, 119545

Reisser J, Shaw J, Wilcox C, Hardesty BD, Proietti M, Thums M, Pattiaratchi C (2013): Marine plastic pollution in waters around Australia: characteristics, concentrations, and pathways. PLoS One 8, e80466

Santana MFM, Dawson AL, Motti CA, van Herwerden L, Lefevre C, Kroon FJ (2021): Ingestion and Depuration of Microplastics by a Planktivorous Coral Reef Fish, Pomacentrus amboinensis. Frontiers in Environmental Science 9

Schlawinsky M, Santana MFM, Motti CA, Martins AB, Thomas‐Hall P, Miller ME, Lefèvre C, Kroon FJ (2022): Improved microplastic processing from complex biological samples using a customized vacuum filtration apparatus. Limnology and Oceanography: Methods 20, 553-567

Uthicke S (1999): Sediment Bioturbation and Impact of Feeding Activity of Holothuria (Halodemia) atra and Stichopus cloronotus, Two Sediment Feeding Holothurians, at Lizard Island, Great Barrier Reef. Bulletin of Marine Science 64, 129 - 141

Wootton N, Ferreira M, Reis-Santos P, Gillanders BM (2021): A Comparison of Microplastic in Fish From Australia and Fiji. Frontiers in Marine Science 8

Wootton N, Reis-Santos P, Adyel T, Blewitt M, Clarke B, Crutchett T, Hamann M, Hardesty D, Lavers J, Leterme S, Leusch F, Lynch S, Motti C, O’Brien A, Okoffo E, Perera K, Puskic P, Razzell HJ, Roman L, Santana MFM, Snigirova A, Tuuri EM, Wilson S, Ziajahromi S, Gillanders BM (2024): Marine sampling field manual for microplastics. In: Przeslawski R FS (Editor), Field Manuals to Monitor Australian Waters. National Environmental Science Program

Zhang X, Liu Z, Zhao Y, Ma P, Colin C, Lin AT-S (2022): Distribution and controlling factors of microplastics in surface sediments of typical deep-sea geomorphological units in the northern South China Sea. Frontiers in Marine Science 9
